# Rapid Forensic DNA Profiling via Real-Time Recombinase Polymerase Amplification of InDel Markers

**DOI:** 10.3390/bios16020106

**Published:** 2026-02-06

**Authors:** Liesl De Keyzer, Sonja Škevin, Koen Deserranno, Dieter Deforce, Filip Van Nieuwerburgh

**Affiliations:** Laboratory of Pharmaceutical Biotechnology, Ghent University, Ottergemsesteenweg 460, 9000 Ghent, Belgium; liesl.dekeyzer@ugent.be (L.D.K.); sonja.skevin@ugent.be (S.Š.); koen.deserranno@ugent.be (K.D.); dieter.deforce@ugent.be (D.D.)

**Keywords:** recombinase polymerase amplification, InDel genotyping, forensic genetics, DNA probes, lab-on-a-chip

## Abstract

Forensic DNA profiling commonly relies on polymerase chain reaction (PCR) amplification followed by capillary electrophoresis (CE) or massively parallel sequencing (MPS), which requires expensive, laboratory-based equipment that depends on a stable power supply and is unsuitable for field applications. Here, we present a proof-of-concept assay that uses recombinase polymerase amplification (RPA) combined with exo probe detection for rapid, isothermal genotyping of insertion–deletion (InDel) markers. To the best of our knowledge, this study represents the first demonstration of forensic DNA typing using RPA coupled with exo probes. The reaction proceeds at 39 °C and combines amplification and detection in a single 20 min step. Thirteen DNA samples were genotyped in triplicate across eight InDel loci using allele-specific fluorescent probes. Genotypes were derived from differential endpoint fluorescence between matched and mismatched probes. Compared with benchmark genotyping, 97.07% of genotypes (*n* = 307) were correct at 1 ng DNA input. Accurate profiles were reliably obtained for DNA inputs as low as 250 pg, and partial profiles were still detectable at 31 pg. The results demonstrate that RPA-based InDel genotyping is fast, sensitive, and reproducible. With further optimization, such as refined probe design and selection of robust loci, the assay has clear potential to achieve complete accuracy and to be integrated into portable lab-on-a-chip platforms for rapid, field-deployable forensic identification.

## 1. Introduction

Current forensic DNA profiling typically relies on genotyping of short tandem repeats (STRs), which are genomic loci with repetitive units of one to six base pairs. The number of repeats at each locus varies among individuals, making STRs highly informative markers for person identification and kinship analysis [1,2]. The gold standard for forensic STR genotyping involves polymerase chain reaction (PCR) amplification, followed by either capillary electrophoresis (CE) or massively parallel sequencing (MPS). While these methods provide high accuracy and throughput, they require a large capital investment and a connection to the power grid, limiting their application to dedicated lab facilities [3].

There is an increasing demand for portable and rapid DNA profiling devices, suitable for use in field conditions such as disaster zones, conflict areas, remote clinics, or mobile forensic units, where access to centralized laboratory infrastructure is limited or unavailable. Current commercially available portable DNA devices include ANDE-6C (ANDE Corporation, Longmont, CO, USA), RapidHIT™ ID (Applied Biosystems, Thermo Fisher, Waltham, MA, USA) and Quick TargSeq™ (CapitalBio Technology, Beijing, China). However, these devices remain relatively large, weighing over 29 kg, and rely on an external power supply, limiting their suitability for widespread use in low-resource settings [4,5,6].

Lab-on-a-chip (LoC) technology offers a promising portable alternative to conventional genotyping methods, as it enables cost-effective analysis by using small reaction volumes. Additionally, LoC systems are known for streamlined workflows and minimal operation steps, making them user-friendly for field applications [7,8]. However, integrating gold-standard DNA genotyping into LoC platforms presents two key challenges. First, microchip CE often suffers from reduced sensitivity and peak resolution [9,10]. Second, PCR requires precise thermal cycling, demanding highly calibrated and bulky heating systems, as well as high power consumption, which is not favourable for low-resource settings [11].

To overcome these limitations, researchers have increasingly explored isothermal amplification methods that eliminate the need for thermal cycling. Among these methods, recombinase polymerase amplification (RPA) has gained special attention for its simple primer design, speed and sensitivity [12]. RPA relies on T4 UvsX recombinase, T4 UvsY helper proteins, single-stranded DNA binding proteins (GP32), and a strand-displacing Bsu polymerase to amplify target DNA under isothermal conditions [13,14]. Since its introduction by Piepenburg et al. in 2006 [15], RPA has been used for different applications, including real-time pathogen detection, molecular diagnostics, and food safety testing. The technique typically completes within 5 to 20 min, and operates optimally at low temperatures (37–42 °C). Additionally, RPA tolerates common PCR inhibitors, including hemoglobin, heparin and ethanol, allowing direct testing of biofluids like serum, urine, blood or saliva with minimal sample preparation [12,13]. Our previous work demonstrated that RPA could be incorporated into CE and MPS workflows for STR genotyping [16], providing early evidence that it may be suitable for forensic DNA profiling.

Probe hybridization-based assays are a straightforward and microfluidics-compatible alternative to CE profiling. Although not impossible, the application of probes to STRs is challenging due to their length and repetitive nature, which makes it difficult to design allele-specific probes. As a result, only a few probe-based STR genotyping assays have been reported [17], including our previously published STRide probes [5] and QueSTR probes [18,19], which are limited to relatively short loci such as TH01 (6–10 repeats) and D16S539 (9–13 repeats). InDel markers, by contrast, consist of small insertions or deletions of one or more base pairs, and are biallelic and non-repetitive, which eliminates stutter artefact formation and permits shorter probe design [20,21]. These features make InDels well-suited for hybridization-based assays and for analysis of degraded DNA. Moreover, their binary nature simplifies data interpretation, which could reduce reliance on highly trained personnel in low-resource settings [17]. The relevance of InDels in forensic genotyping is further demonstrated by their inclusion in commercial kits such as the InnoTyper^®^ 21 kit (InnoGenomics, New Orleans, LA, USA).

Here, we present a proof-of-concept forensic genotyping assay that combines RPA and exo probe detection for rapid genotyping of seven InDel loci for human identification and the amelogenin sex marker within a 20 min reaction. To our knowledge, all previously reported probe-based genotyping approaches for either InDels or STRs have relied on PCR amplification [5,17,18,21,22]. This study evaluates three critical aspects of forensic DNA profiling: accuracy, robustness and sensitivity. To assess accuracy and robustness, thirteen samples were analyzed in triplicate. Sensitivity was assessed using DNA inputs ranging from 500 to 31 pg, with the latter equivalent to the amount of DNA in 6 cells. As a proof-of-concept study, extensive validation and optimization were limited to key reaction parameters, including temperature, and dNTP, probe, and primer concentrations.

## 2. Materials and Methods

### 2.1. Sample Collection and Purification

Genotyping was performed on four commercially available reference DNA samples, HG001 (NA12878), HG002 (NA24385), HG005 (NA24631) and HG00339 (Coriell Institute for Medical Research, Camden, NJ, USA), as well as nine blood samples collected from anonymous, healthy volunteers. All participants provided informed consent, and ethical approval was obtained from the ethical review board of Ghent University Hospital (approval No. BC-05557). No sample preparation was required for the DNA reference samples. Blood samples were collected in a K3E K3EDTA Minicollect^®^ collection tube (Greiner Bio-One, Kremsmünster, Austria), using a 21 G Minicollect^®^ Lancelino safety lancet (Greiner Bio-One, Kremsmünster, Austria) with a penetration depth of 2.4 mm for finger puncture. DNA extraction was performed using the DNeasy^®^ Blood and Tissue kit (Qiagen, Helden, Germany) according to the manufacturer’s protocol. Both the DNA standards and the DNA extracts from blood were diluted to a final concentration of 1 ng/µL before further use.

### 2.2. Panel of InDel Markers

A panel of seven InDel loci, along with Amelogenin, was selected from three published forensic InDel panels for human identification [21,22,23]. The selection followed five criteria: (1) only biallelic markers were included to ensure assay simplicity; (2) the minor allele frequency (MAF) ranged from 0.25 to 0.5, corresponding to an approximate discrimination power (DP) > 0.35; (3) all selected InDels were intron variants (4) insertion sequences were limited to 5–15 bp; (5) each insertion contained two thymine residues positioned within 2–5 nucleotides of each other. The final criterion is essential for exo probe design, since both the fluorophore and quencher are thymine-bound and must lie within five nucleotides for efficient quenching. With the exception of amelogenin, all loci were assigned ID codes (ID01–ID07) corresponding to dbSNP RefSNP numbers rs57943214, rs67320356, rs11281372, rs34159280, rs58092599, rs66739142, and rs3067397, respectively. Genomic location, alleles, MAF and DP for each locus are provided in Supplementary Table S1.

### 2.3. Benchmark Genotyping Using PCR Amplification

Benchmark genotyping of the purified blood samples was performed for all InDel loci by Sanger sequencing (Eurofins Genomics, Ebersberg, Germany). Extracted DNA (2 ng) was first amplified by PCR in a 50 µL reaction containing 1 × PCR buffer (Qiagen, Helden, Germany), 0.2 mM dNTPs (Thermo Fisher Scientific, Waltham, MA, USA), 0.5 mM MgCl_2_ (Qiagen, Helden, Germany), 0.5 mM BSA (Thermo Fisher Scientific, Waltham, MA, USA) and 5 U Taq polymerase (Qiagen, Helden, Germany). PCR primers were designed according to the manufacturer’s recommendations and used at a final concentration of 0.5 µM (Supplementary Table S2). Thermal cycling conditions included an activation step at 95 °C for 15 min, followed by 33 cycles of denaturation (94 °C, 1 min), annealing (57 °C, 1 min), and elongation (72 °C, 1.2 min), with a final extension at 72 °C for 10 min. PCR products were purified using AMPure XP Beads (Beckman Coulter Life Sciences, Indianapolis, IN, USA) following the manufacturer’s instructions, and quantified with a Qubit 2.0 fluorometer (Thermo Fisher Scientific, Waltham, MA, USA). Each purified sample was diluted to 1 ng/µL and sent to Eurofins Genomics (Ebersberg, Germany) together with a specific sequencing primer for each InDel locus. Sequence data were analyzed with the SnapGene 8.0 software (Dotmatics, Boston, MA, USA). For homozygous samples, genotypes were assigned based on the presence or absence of the insertion sequence, whereas heterozygous samples showed overlapping fluorescence peaks at the InDel site, consistent with simultaneous detection of both alleles.

### 2.4. RPA Primer Design

Primers were designed according to the guidelines described by Piepenburg et al. (2006) [15] and the TwistDx Assay Design Manual. The following criteria were applied: (1) amplicon size was limited to 1 kb; (2) primer length ranged from 30 to 35 nucleotides; (3) GC content was between 30 and 70%; (4) primers contained no more than three consecutive identical nucleotides; (5) there were ≤2 complementary bases at the 3′ ends of primers within a pair; (6) although a primer’s melting temperature is not a critical determinant for its RPA efficiency, primer pairs were selected to have melting temperatures within 5 °C of each other. The complete list of primer sequences is provided in Supplementary Table S2.

### 2.5. InDel Probe Design

**Figure 1:**
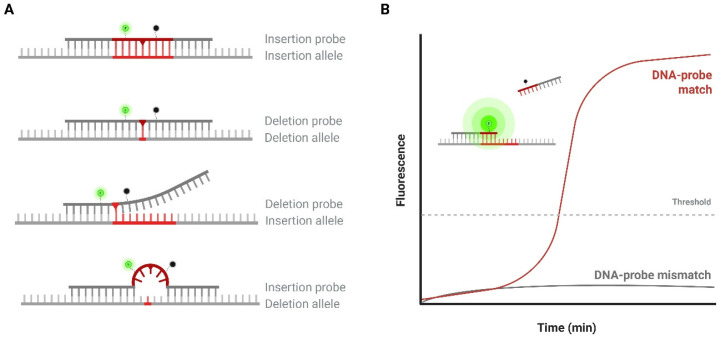
(**A**) Design of the InDel probes. InDel markers are highlighted in red. Each probe contains a tetrahydrofuran (THF) residue (red triangle), and is labelled with a FAM fluorophore (green) and a BHQ-1 quencher (black). Hybridization to the complementary allele in the presence of exonuclease III results in probe cleavage and fluorescence. Mismatched probes show reduced duplex stability and fluorescence intensity. (**B**) Illustration of a real-time RPA plot. Fluorescence is measured over time. Perfectly matched probes yield sigmoidal curves with higher endpoint values than mismatched probes.

TwistAmp^®^ exo probes (TwistDx, Maidenhead, UK) were incorporated into the RPA reaction mix for real-time genotyping. These oligonucleotide probes contain a tetrahydrofuran (THF) residue, which separates a 6-carboxyfluorescein (FAM) fluorophore from a Black Hole Quencher (BHQ) (acquired from LGC Biosearch Technologies, Hoddesdon, UK). Two probes were designed for each InDel locus (Supplementary Table S2), either perfectly complementary to the insertion or to the deletion allele, hereafter referred to as the insertion probe and deletion probe, respectively (Figure 1A). The target DNA samples were analyzed by dividing them into two separate reactions, each containing a different probe, resulting in two amplification curves. When a probe hybridizes to its complementary InDel allele in the presence of exonuclease III, the enzyme cleaves the probe at the THF moiety, separating the fluorophore and quencher, which results in fluorescence (Figure 1B). In contrast, a mismatched probe-target duplex is less stable, resulting in less cleavage and lower fluorescence values. This difference in endpoint fluorescence between matched and mismatched probes was used to determine a sample’s genotype for a given InDel locus.

### 2.6. RPA-Based Genotyping

RPA was performed using the TwistAmp^®^ Liquid Exo kit (TwistDx, Maidenhead, UK), following the manufacturer’s instructions. A master mix was prepared to obtain final concentrations of 1 × Reaction Buffer, 1 × Basic E-mix, 1 × Core Reaction Mix and 1 × Exo Mix, with dNTPs (Thermo Fisher Scientific, Waltham, MA, USA) at a final concentration of 1.8 mM. For loci ID02 and ID07, the dNTP concentration was increased to 2.4 mM to improve amplification efficiency. The master mix was added to the primers and probe with a final concentration of 420 and 120 nM, respectively, after which 1 ng of DNA template was added. The reaction was performed in a final reaction volume of 15 µL and was initiated by adding MgOAc at a final concentration of 14 mM. The reaction mix was inverted 6 times before being placed in the AriaDx Real-Time PCR system (Agilent, Santa Clara, CA, USA). Amplification proceeded at 39 °C with fluorescence readings taken every 30 s. After 4 min of incubation, samples were briefly mixed by another 6 full inversions, before continuing real-time detection for the remainder of the 20 min run.

## 3. Results

### 3.1. Benchmark Genotyping

Sanger sequencing results are shown in Supplementary Figure S1. To determine the genetic diversity of the sample cohort, the minor genotype frequency (MGF) was calculated as the number of participants with the least frequent genotype divided by the total number of participants. According to Mendelian inheritance expectations, an ideal MGF approaches 0.25 for a balanced representation of all genotypes [3]. However, for this proof-of-concept study, the presence of at least one sample carrying the minor genotype (MGF = 0.08) was considered sufficient to evaluate assay performance. This criterion was met for all eight loci.

### 3.2. Genotype Calling Strategy and Threshold Determination

**Figure 2:**
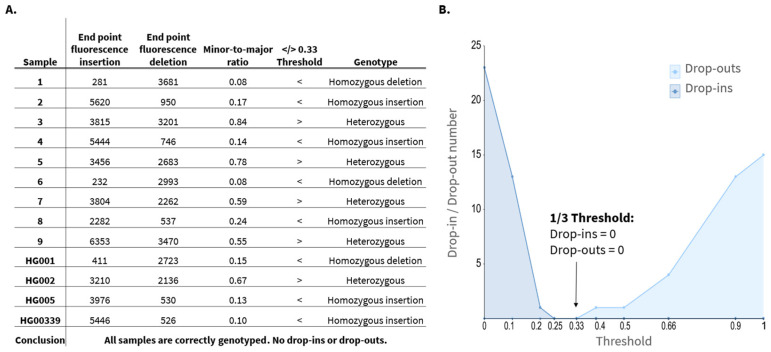
(**A**) Calculation of the minor-to-major end point fluorescence ratio for a representative locus (ID03), given for the first replicate of each sample. Ratios below the 0.33 threshold indicate homozygosity, while higher ratios indicate heterozygosity. (**B**) ID03 drop-in and drop-out rates as a function of potential thresholds, as calculated for all samples in triplicate. A 0.33 cutoff yields the lowest combined error rate. Therefore, a sample is genotyped as heterozygous for ID03 in case the endpoint fluorescence of the minor curve exceeds 1/3 of the endpoint fluorescence of the major curve.

After obtaining the raw real-time RPA fluorescence curves, baseline normalization was applied by subtracting the signal at T = 0 min from all subsequent readings. Based on these results, the minor and major curves were defined as the curve with the lowest and highest endpoint fluorescence, respectively. Samples were excluded for a given locus if the major-allele end point fluorescence did not exceed 1000 units after normalization. This cutoff was determined from the no template control (NTC) samples (Supplementary Figure S2), in which the highest background signal reached 486 units for locus ID04. To account for variability, a safety factor of 2 was applied, yielding a conservative threshold of 1000 units. Signals below this threshold were considered insufficient for reliable genotyping. Based on this criterion, only 1 replicate (sample 8, replicate 3, locus 7) was excluded from analysis (*n* = 311/312). Next, the time at which fluorescence first reached the 1000-unit threshold was determined. In some cases, this value occurred before 5 min, producing noisy, fluctuating curves likely caused by air bubble formation or condensation in the reaction wells, interfering with fluorescence readings. Four such cases were excluded from further analysis (*n* = 307/312). To establish a heterozygosity cutoff, the minor-to-major end point fluorescence ratio was calculated for all remaining samples. For each locus, these ratios were evaluated across multiple potential thresholds: 0, 0.1, 0.2, 0.25, 0.33, 0.4, 0.5, 0.66, 0.9, and 1. Figure 2A illustrates an example for locus ID03, where endpoint fluorescence ratios are compared to potential threshold of 0.33 to classify homozygous or heterozygous genotypes. For each threshold, drop-in and drop-out error rates were determined by comparing the calculated genotype with the true genotype. A drop-in was defined as a homozygous sample in which the minor curve exceeded the threshold, falsely indicating heterozygosity. A drop-out occurred when a heterozygous sample’s minor curve fell below the threshold, resulting in a false homozygous call. An overview of the drop-in and drop-out rates for locus ID03 is shown in Figure 2B, demonstrating that the lowest combined error rate was observed at a threshold of 0.33. Accordingly, samples were classified as heterozygous for this locus if the endpoint fluorescence of the minor curve exceeded 1/3 (33%) of the major curve’s endpoint value. Otherwise, the sample was considered homozygous. 0.33 was the optimal threshold for all loci except for ID06, where a threshold of 0.4 yielded the lowest combined error rate (Supplementary Figure S3).

### 3.3. Genotyping Accuracy and Assay Robustness

**Figure 3:**
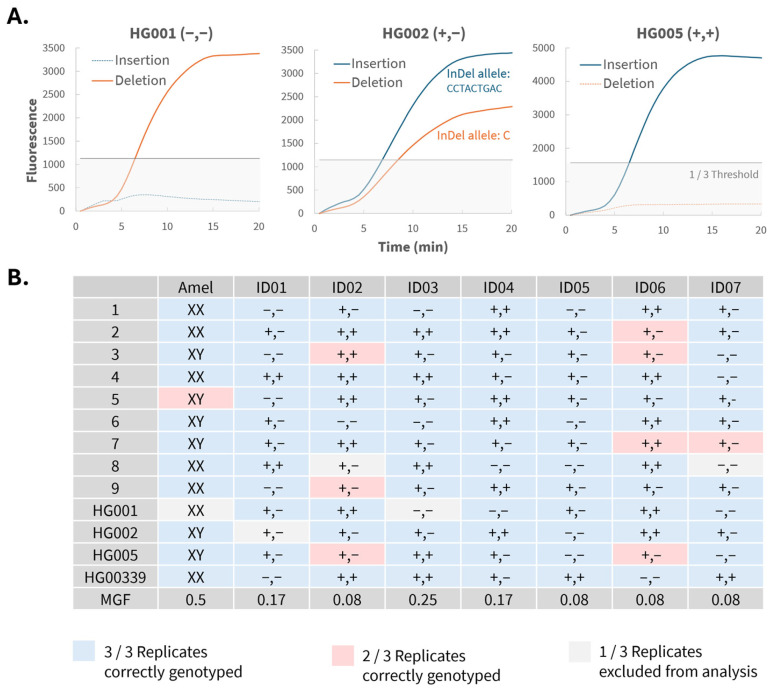
(**A**) Real-time RPA graphs of a representative locus (ID03) for samples HG001(−,−), HG002 (+,−) and HG005 (+,+), using 1 ng of input DNA. Curves of true alleles are shown as solid lines, curves of mismatched probes are shown as dashed lines. The grey line marks the heterozygosity threshold of 1/3 of major curve endpoint fluorescence. (**B**) True genotypes and overview of the results for all samples and loci. Each cell represents a sample tested in triplicate: blue = all replicates are correctly genotyped, pink = one replicate is incorrectly genotyped, grey = one replicate was excluded. True genotypes are given in each cell: +,+ = homozygous insertion; −,− = homozygous deletion; +,− = heterozygous.

Figure 3A presents an example of real-time RPA curves for the three possible InDel genotypes at a representative locus, ID03 (rs11281372). Equivalent analyses were performed for the other loci included in the panel. In this example, HG001 was correctly genotyped as homozygous deletion, HG002 as heterozygous and HG005 as homozygous insertion. Curves corresponding to true alleles show a sharp increase in fluorescence after approximately 5 min, reaching endpoint values above 1000 units. Mismatched probes show a small increase in fluorescence after about 7 min, with endpoint values below 1/3 of the endpoint value of the major curve. Figure 3B summarizes the true genotypes, MGFs, and allgenotyping results across all samples and loci. The corresponding real-time RPA graphs are given in Supplementary Figures S4–S11. Each sample was analyzed in triplicate on different days. Genotyping errors were infrequent and largely locus-specific rather than stochastic, indicating stable and robust assay performance rather than random failure. In total, 9 out of 307 RPA curves led to incorrect genotypes (2 allelic drop-ins and 7 allelic drop-outs), resulting in an accuracy percentage of 97.07%. The Scientific Working Group on DNA Analysis Methods (SWGDAM) does not specify a fixed accuracy threshold for forensic genotyping methods, but instead recommends a holistic approach that includes replicate testing, statistical validation and careful design [24,25]. Therefore, this accuracy percentage was deemed sufficient for this proof-of-concept study.

### 3.4. Genotyping Errors and Locus-Specific Optimization

**Figure 4:**
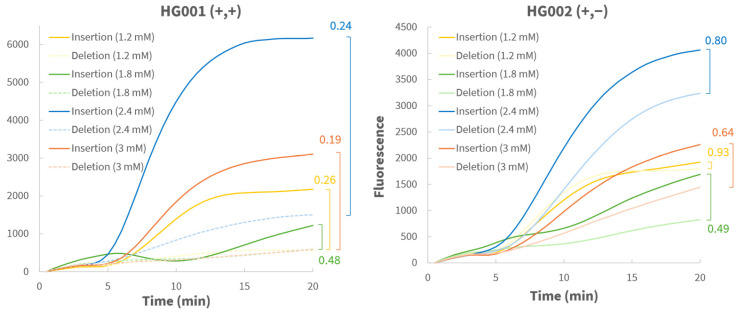
Real-time RPA curves of locus ID02 using HG001 (+,+) and HG002 (+,−) at different dNTP concentrations (1.2, 1.8, 2.4, and 3.0 mM) with 1 ng of input DNA. The optimal reaction with 2.4 mM dNTPs showed the best efficiency and specificity, as reflected in the end point fluorescence and minor-to-major fluorescence ratios shown to the right of each graph.

Locus ID02 and ID07 initially showed low amplification efficiency, with endpoint fluorescence frequently below the 1000-unit threshold required for confident genotype calling. To improve performance, a stepwise pilot optimization was conducted for locus ID02 (rs67320356), using one representative homozygous sample (HG001) and heterozygous sample (HG002). First, the dNTP concentration was systematically increased from 1.2 to 3.0 mM (Figure 4). For each concentration, the minor-to-major end point fluorescence ratio was calculated to assess reaction specificity. At 2.4 mM, both specificity and efficiency were optimal: homozygous samples showed strong major-curve fluorescence and low ratios, while heterozygous samples showed both high endpoint fluorescence and high ratios. Although increasing the dNTP concentration also resulted in higher fluorescence from mismatched probes, potentially due to non-specific amplification or primer dimer formation, fluorescence from true alleles increased proportionally. As a result, minor-to-major fluorescence ratios of homozygous samples remained stable across all tested dNTP concentrations. Subsequently, varying primer (420, 500, 600 nM) and probe (120, 150, 200 nM) concentrations were tested, which confirmed that the standard conditions (420 nM primers and 120 nM probe) provided the best balance between efficiency and specificity (Supplementary Figure S12). Finally, reaction temperatures between 37 and 42 °C were evaluated (Supplementary Figure S13). Overall, correct genotyping was achieved across all temperatures. Specificity was highest at 37 °C for the homozygous sample and at 38 °C for the heterozygous sample. Although higher temperatures increased overall fluorescence, this did not improve specificity. Therefore, a compromise temperature of 39 °C was selected, as it yielded reliable results for both genotypes. The optimized conditions identified for locus ID02 (i.e., an increased dNTP concentration to 2.4 mM) were subsequently applied to locus ID07, improving its overall performance, though occasional allelic drop-outs remained for both loci (Figure 3B).

**Figure 5:**
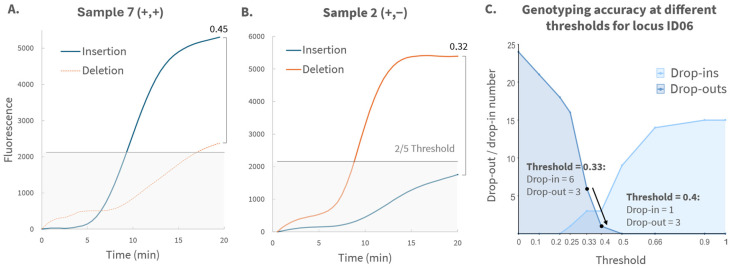
(**A**) Example of a false heterozygous call for homozygous insertion Sample 7 (+,+) at locus ID06, where elevated deletion probe fluorescence causes the minor-to-major end point ratio (given above the bracket on the right) to exceed the 0.4 threshold. (**B**) Example of a false homozygous classification in heterozygous Sample 2 (+,−), where reduced insertion probe fluorescence prevents the minor-to-major ratio from crossing the 0.4 threshold. (**C**) Threshold study for locus ID06, showing a decreased error rate for a genotyping threshold of 0.4 compared to 0.33.

Locus ID06 (rs66739142) showed the highest genotyping error rate, characterized by two recurrent types of misclassification. First, homozygous insertion samples frequently displayed elevated fluorescence for the deletion probe (Figure 5A), causing false heterozygous calls. Second, heterozygous samples showed reduced fluorescence for the insertion probe, leading to incorrect homozygous deletion classification (Figure 5B). Increasing the ID06 heterozygosity threshold from 0.33 to 0.40 reduced the drop-in rate for homozygous samples without affecting the drop-out rate for heterozygous samples, as shown in Figure 5C. We therefore adopted a locus-specific threshold for ID06, improving overall allele classification. When applied consistently within the locus and without genotype bias, such an independent threshold does not compromise objective genotype calling. To further explore this elevated error rate, both insertion and deletion probes of all loci were aligned to the human genome using the NCBI Nucleotide Blast^®^ tool (https://blast.ncbi.nlm.nih.gov/Blast.cgi, accessed on 7 January 2026), which confirmed that cross-reactivity was not responsible for the elevated deletion-probe fluorescence for ID06. Instead, four sequence features likely contributed to performance issues for this locus (Table S3): low GC content (<30%), low melt temperature (>70 °C), the presence of poly-A stretches, and repetitive motifs, all of which can affect hybridization kinetics [26,27]. Alternative probe designs could potentially improve performance at this locus, though such optimization was not pursued in this proof-of-concept study.

### 3.5. Sensitivity Assessment

**Figure 6:**
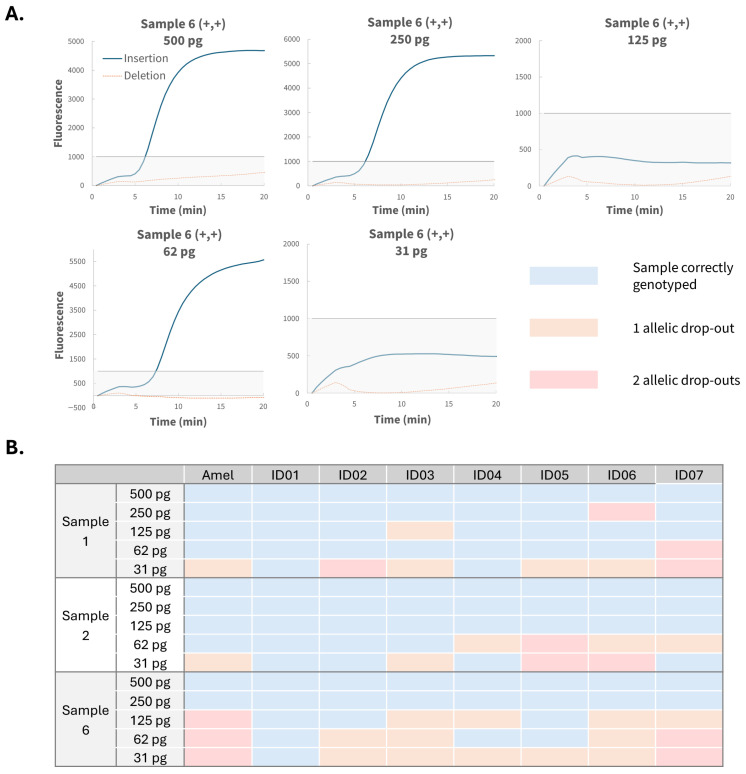
(**A**) Example of the sensitivity results for a representative sample (Sample 6; +,+) at locus ID04 with DNA inputs ranging from 500 to 31 pg. True alleles are indicated with solid lines, mismatched probes with dashed lines. The grey line marks the 1000 unit threshold. (**B**) Overview of sensitivity results for samples 1, 2 and 6 across all loci and DNA inputs. Each cell represents one tested sample for a specific DNA input, with colour indicating accuracy: blue = correctly genotyped, orange = one allelic drop-out, pink = two allelic drop-outs.

To evaluate assay sensitivity, samples 1, 2 and 6 were tested at DNA inputs of 500, 250, 125, 62 and 31 pg, using the same protocol as for 1 ng input. Figure 6A shows the real-time RPA curves of a representative sample (Sample 6, homozygous insertion) at locus ID04 (rs34159280), where a single drop-out was observed at 125 and 31 pg, but not at 62 pg. A summary of all sensitivity results is shown in Figure 6B. The corresponding real-time RPA graphs are given in Supplementary Figures S14–S21. With the exception of one double drop-out at 250 pg for locus ID06, all drop-outs occurred at input levels of 125 pg or lower. These results indicate that complete genetic profiles were obtained for all but one sample at a total DNA input of 4 ng (250 pg per reaction × 2 reactions per locus × 8 loci). Of the 34 curves in which allelic drop-outs were observed, only two cases (6%) were attributable to the minor curve falling below the heterozygosity threshold. In all other cases, samples were excluded because the major-allele end point fluorescence did not exceed the 1000-unit threshold after normalization, demonstrating the robustness of the applied genotyping criteria even at low DNA inputs. Furthermore, no differences were observed for loci requiring higher dNTP concentrations (ID02 and ID07) compared with other loci at DNA inputs down to 31 pg, providing strong evidence that increased dNTP levels does not influence genotype calling through amplification artefact formation, even in low-template samples.

## 4. Discussion

Conventional forensic DNA profiling is typically performed through PCR amplification, followed by capillary electrophoresis (CE) or massively parallel sequencing (MPS) [3,28]. While these methods deliver high sensitivity, accuracy and throughput, they require expensive instrumentation, trained personnel and complex multi-step workflows, limiting their applicability in rapid or field-based forensic analysis. In contrast, the real-time RPA reaction developed in this study is a fast and straightforward alternative. The assay achieves reproducible results within 20 min, demonstrating correct genotypes in 97.07% of cases (*n* = 307), and complete profiles for DNA inputs as low as 250 pg, with partial success at 31 pg. These findings confirm the assay’s technical feasibility as a proof-of-concept. The current InDel panel provides a discrimination power ranging from 1/30, as obtained by multiplying the highest allele frequencies of all loci, to 1/1000 for the minor allele frequencies (MAF). To further enhance both discrimination power and accuracy, future efforts should focus on expanding the number of loci, refining the probe design, and carefully selecting robust markers to minimize locus-specific errors as observed for ID06 (rs66739142). This locus consistently exhibited the highest error rate, both at high (1 ng) and low DNA inputs. Based on the characteristics of the ID06 probe sequences, we recommend probe designs with a GC content above 30%, a melting temperature above 70 °C, homopolymer stretches shorter than five nucleotides, and the absence of repetitive motifs longer than 6 nucleotides.

In addition, the optimization of reaction chemistry, including the adjustment of the RPA protein concentrations (UvsX, UvsY and gp32) or buffer composition, could further improve amplification fidelity and overall assay performance. For example, Cordoba-Andrade et al. (2025) demonstrated that especially the gp32 concentration plays a critical role in RPA efficiency and primer-dimer formation [29]. Such adjustments are currently constrained by the nature of the TwistAmp^®^ Liquid Exo kit used here, which provides pre-assembled reagent mixes with undisclosed enzyme formulations and concentrations, thereby limiting the ability to fine-tune protein or buffer ratios. A potential solution lies in newer formulations such as the Lyo-ready RPA kit by Invitrogen (Thermo Fisher, Waltham, MA, USA), which provides individual RPA components at defined concentrations, enabling more precise fine-tuning of reaction conditions for future assay optimization. Additionally, implementing a multiplex set-up will be essential to streamline assay design and improve scalability. Multiplex RPA assays with probe detection have been successfully demonstrated for up to five targets [30,31,32,33], with the main constraints being a cumulative primer and probe concentration of 2000 nM and spectral overlap between fluorophores [12]. Continued research into higher-order multiplexing strategies is expected to enable further expansion of RPA-based genotyping panels, while reducing the sample preparation workload.

Another important consideration for future validation is the performance of the assay on complex or compromised samples. Forensic casework often contains degraded, mixed, or low-template DNA, and the robustness of RPA-based genotyping under such challenging conditions should be systematically evaluated for integration into routine workflows. InDel markers are inherently well-suited for degraded DNA due to their short amplicon sizes (<200 bp) and simple, biallelic structure. For instance, Li et al. (2021) demonstrated successful recovery of full profiles across 18 InDel loci from artificially degraded DNA following 80 min of boiling-water treatment [34]. In theory, the real-time RPA approach described here could also be applied to mixed samples containing DNA from multiple contributors, especially when two templates are present at different input ratios. In such cases, the amplification curves of the lower-abundance contributor are expected to exhibit delayed sigmoidal fluorescence increases, analogous to the ΔCt relationship observed in qPCR. A forensic mixed-sample real-time RPA assay has not been tested yet and falls outside the scope of this proof-of-concept study. However, this notion is supported by a study performed by Li et al. (2025), where rapid quantification through real-time RPA was reported with a detection limit of 50 copies/µL for the tetracycline resistance gene [35]. Although quantitative resolution would not be required for rapid on-site genotyping, these findings suggest that RPA-based detection could discriminate between contributors of markedly different template quantities, for example, in mixed forensic samples. We expect that differentiation between a 1:1 homozygous mixture and a true heterozygote is not possible with the current assay. However, when using all 8 InDel loci, the assay can be useful for cases where one or both profiles are already known or accounted for on a crime scene.

The sensitivity assessment showed complete profiles for all but one sample at a singleplex DNA input of 250 pg. Direct comparison of the assay’s sensitivity to gold standard genotyping would not be appropriate, as PCR-CE workflows are extensively optimized and automated, typically generating full profiles at DNA inputs of 100 pg–1 ng [28]. Nonetheless, the observed sensitivity is comparable to existing rapid DNA technologies. For instance, the RapidHIT system typically requires 5–10 ng of input DNA to generate full profiles [36] and has been successfully used for high-yield forensic samples such as disaster victim identification [37] and reference buccal swabs [38]. In such cases, mobility and turnaround time outweigh concerns about sensitivity limitations [39,40]. While the RapidHIT platform performs multiplex genotyping in a single reaction, our current assay requires parallel reactions per locus and has inherently lower discrimination power due to the binary nature of InDels compared to STRs, which have at least 8 alleles per locus. As indicated above, future studies should investigate panel expansion and multiplexing to address these problems.

In addition, our assay offers key advantages in terms of reaction speed and portability, as it can be completed in a significantly shorter time frame and has the potential to be integrated into lab-on-a-chip platforms, which could dramatically improve on-site genotyping in resource-limited or remote settings. Transitioning from CE to probe-based detection and from PCR to RPA significantly simplifies integration into such platforms by eliminating the need for complex thermal cycling equipment. Although RPA optimally works at temperatures between 37 and 42 °C, the technique is known to tolerate a much broader temperature range from 22 to 45 °C, without stringent thermal control [12]. This wide operational window is a key advantage for use in uncontrolled environments compared to PCR. For this proof-of-concept study, a benchtop AriaDx real-time system (Agilent, Santa Clara, CA, USA) was used to enable controlled evaluation of the RPA reaction chemistry through real-time fluorescence detection, as it allows for systematic optimization and validation of genotype calling criteria. Future studies should evaluate the assay’s compatibility with hand-held, battery-powered heating devices, as well as its integration into microfluidic platforms. Such systems would likely employ a multi-cavity chip design enabling spatial separation of insertion and deletion probes, combined with small reaction volumes, preloaded lyophilized RPA reagents, and a simple endpoint fluorescence readout. As RPA is compatible with various biofluids, including blood, saliva, semen and stool, without the need for extensive purification beforehand, it supports straightforward sample-in-answer-out workflows [12].

To the best of our knowledge, this study is the first to apply RPA combined with exo probes for forensic DNA genotyping. Previous RPA-based approaches have been limited to confirming the presence of human or male DNA [41,42,43], while our method enables both amelogenin sex typing and individual identification through InDel markers. It should be noted that state-of-the-art forensic DNA profiling continues to rely primarily on STR analysis. However, probe-based detection under low-temperature, isothermal conditions of an RPA reaction might be challenging in repetitive or highly similar sequence contexts, due to their large amplicon sizes and highly repetitive sequences. InDel polymorphisms are a simpler and robust alternative. They are biallelic, generate short amplicons, and exhibit low mutation rates, making them well-suited for degraded or low-quality DNA samples, or when stutter products complicate STR profiling [21]. Transitioning from STRs to InDel markers poses certain challenges, including reduced discrimination power and the lack of compatibility with existing DNA databases, such as the Combined DNA Index System (CODIS) and the European Standard Set (ESS) [44,45]. Nevertheless, database compatibility and maximal discrimination power are not essential for the intended applications of this assay, which include prolonging custody terms while awaiting more extensive lab results, excluding suspects, and comparing samples within a case or within a crime scene (e.g., to assess whether different blood spots on a crime scene are from one or multiple contributors). As such, our approach is not intended to replace gold-standard STR genotyping, but could serve as a complementary tool for early triage of samples, reducing the load or sample backlog on centralized laboratories, and improving turnaround times in time-critical forensic contexts [36,46,47].

## 5. Conclusions

This proof-of-concept study demonstrates that recombinase polymerase amplification (RPA) combined with exonuclease probe detection enables rapid, isothermal genotyping of InDel markers for forensic DNA profiling. The assay achieved 97.07% accuracy at 1 ng DNA input, with reliable genotyping down to 250 pg and partial profiles detectable at 31 pg. These results confirm that RPA-based InDel genotyping is a fast, sensitive, and reproducible alternative to conventional PCR-based approaches. Further optimization, such as refined probe design, selection of robust loci, and multiplexing, could enable complete accuracy and integration into portable lab-on-a-chip devices, providing a practical solution for rapid, field-deployable forensic identification in resource-limited environments.

## Data Availability

The original contributions presented in this study are included in the article/Supplementary Material. Further inquiries can be directed to the corresponding author.

## Supplementary Materials

The following supporting information can be downloaded at: https://www.mdpi.com/article/10.3390/bios16020106/s1, Table S1: Panel code, dbSNP number, genomic location, alleles, MA, MAF and DP of the eight InDel markers; Table S2: Primer and probe sequences used in the RPA, PCR and Sanger sequencing experiments; Table S3: Error rates as a function of probe sequence features; Figure S1: Sanger sequencing electropherograms showing the true genotype of sample 1–9 for all eight loci; Figure S2: Real-time RPA graphs of all loci obtained for 3 NTC samples in triplicate; Figure S3: Drop-in and drop-out rates as a function of potential thresholds; Figures S4–S11: Real-time RPA graphs of all samples in triplicate for locus Amel, ID01, ID02, ID03, ID04, ID05, ID06, and ID07, respectively; Figure S12: Primer and probe concentration optimization for locus ID02; Figure S13: Temperature optimization for locus ID02; Figures S14–S21: Sensitivity assessment for locus Amel, ID01, ID02, ID03, ID04, ID05, ID06, and ID07, respectively.

## Abbreviations

The following abbreviations are used in this manuscript:

BHQ	Black hole quencher
CE	Capillary electrophoresis
CODIS	Combined DNA Index System
DP	Discrimination power
ESS	European Standard Set
FAM	6-carboxyfluorescein
InDel	Insertion-deletion
LoC	Lab-on-a-chip
MAF	Minor allele frequency
MGF	Minor genotype frequency
MPS	Massively parallel sequencing
NTC	No template control
PCR	Polymerase chain reaction
RPA	Recombinase polymerase amplification
STR	Short tandem repeat
SWGDAM	Scientific Working Group on DNA Analysis Methods
THF	Tetrahydrofuran
