# Supplementary material for: Rapid Forensic DNA Profiling via Real-Time Recombinase Polymerase Amplification of InDel Markers
Source: Biosensors (Basel). 2026 Feb 6;16(2):106. doi: 10.3390/bios16020106 (PMC12938080; doi:10.3390/bios16020106)
Supplement: Supplementary file 1 [file biosensors-16-00106-s001.zip › biosensors-4114732-supplementary.pdf]

Rapid Forensic DNA Profiling via Real-Time Recombinase Polymerase Amplification of InDel Markers

Supplementary file

*Liesl De Keyzer[a], Sonja Škevin[a], Koen Deserranno[a], Dieter Deforce[a], Filip Van Nieuwerburgh[a]**

[a]Laboratory of Pharmaceutical Biotechnology, Ghent University, Ottergemsesteenweg 460, Gent, 9000, Belgium

*To whom correspondence should be addressed. Tel: +32 (0)9 264 80 48; Email: Filip.VanNieuwerburgh@UGent.be

Table S1: Panel code, dbSNP number, genomic location, alleles, minor allele (MA), minor allele frequency (MAF) and discrimination power (DP) of the eight InDel markers included. Genomic locations are given on the latest assembly (GRCh38.p14). MA and MAFs are specified as retrieved from the Global 1000 Genomes Project.

Code	dbSNP	Genomic location	Alleles	MA	MAF	DP
Amel	/	Xp22.1-p22.3 or Yp11.2	AAAGTG / -	/	/	/
ID01	rs57943214	chr7:97136105-97136106	GTATTTGGT / GT	GT	0.4230	0.49
ID02	rs67320356	chr15:66633252-66633258	GAGTTTG / G	GAGTTTG	0.3887	0.48
ID03	rs11281372	chr5:156235246	CTACTGAC / -	-	0.3854	0.47
ID04	rs34159280	chr5:13705834-13705844	CTTCACAA GAC / C	C	0.3860	0.47
ID05	rs58092599	chr12:130458730-130458738	TGTCGTGTC / TGTC	TGTCGTG TC	0.3912	0.48
ID06	rs66739142	chr15:67214196-67214201	TCTTTT / T	T	0.2525	0.38
ID07	rs3067397	chr1:61320635-61320641	TATCTTA / TA	TATCTTA	0.4550	0.50
Combined DP						0.9919

Table S2: Primer and probe sequences used in the RPA, PCR and Sanger sequencing experimental set-up. Fwd = forward. Rev = reverse. S = sanger. [T(FAM)] = 6-Carboxyfluorescein (FAM) fluorophore covalently bound to a thymine residue. [THF] = tetrahydrofuran (THF) residue. [T(BHQ-1)] = Black Hole Quencher™ 1 (BHQ-1) covalently bound to a thymine residue.

**Name**	**Function**	**Sequence**
Amel PCR Fwd	Primer	CCCTGGTTATATCAACTTCAGCTA
Amel PCR Rev	Primer	ATCAGAGCTTAAACTGGGAAGCTG
Amel S	Primer	CCCTGGTTATATCAACTTCAGCTA
Amel RPA Fwd	Primer	CCCTGGGCTCTGTAAAGAATAGTG
Amel RPA Rev	Primer	CCATCAGAGCTTAAACTGGGAAGCTG
Amel Insertion	Probe	GAATAGTGGGTGGATTCTTCATCCCAAA[T(FAM)]AA[THF]G[T(BHQ-1)]GGTTTCTCAAGTG
Amel Deletion	Probe	GAATAGTGTGTTGATTCTTTATCCCAGA[T(FAM)]G[THF]T[T(BHQ-1)]CTCAAGTGGTCCT
ID01 PCR Fwd	Primer	CGGGCTAGTCCCTGTATATATT
ID01 PCR Rev	Primer	GAGTATCAAGCTTCAGAAACTCCTA
ID01 S	Primer	CGGGCTAGTCCCTGTATATATT
ID01 RPA Fwd	Primer	ATTAGGATAGATATAAATGTATTTGTCAGAG
ID01 RPA Rev	Primer	CACTCAAGGGTATTAAATTAGTTATAGTTAGT
ID01 Insertion	Probe	GCATTTCTATGCCATTTTCAGTTAATGTAT[T(FAM)]T[THF]G[T(BHQ-1)]TTCACCTGCAATAT
ID01 Deletion	Probe	AATATGCATTTCTATGCCATTTTCAGTTAA[T(FAM)]G[THF]T[T(BHQ-1)]CACCTGCAATATTT
ID02 PCR Fwd	Primer	TGCATACATCGCAAGGCAG
ID02 PCR Rev	Primer	GCACCAGGCTCATCCTATTC

ID02 S	Primer	GGTGCATACATCGCAAGGCAGTG
ID02 RPA Fwd	Primer	GCCTGTCTGTCTCCTTCTTGGGACTGAGTATTC
ID02 RPA Rev	Primer	GATGTCCCTGACGTCCCTGCACTCAGCACCAGG
ID02 Insertion	Probe	TGGGAAATGGCCAGGAACTCAGTAAGAG[T(FAM)]T[THF]G[T(BHQ-1)]CAAATGGGTAAT
ID02 Deletion	Probe	TCTCCTTGGGAAATGGCCAGGAACTCAG[T(FAM)]A[THF]G[T(BHQ-1)]CAAATGGGTAATA
ID03 PCR Fwd	Primer	CCATCAATGGCAGAGACTGAAG
ID03 PCR Rev	Primer	TCCCAAAGTCACATGATTCAACA
ID03 S	Primer	CCATCAATGGCAGAGACTG
ID03 RPA Fwd	Primer	CCATCAATGGCAGAGACTGAAGGATGAAGTT
ID03 RPA Rev	Primer	CTGATATAAATTATCCCAAAGTCACATGATTCA
ID03 Insertion	Probe	ATGAGATTTGGAGGACTGTGCATGTGGCC[T(FAM)][THF]C[T(BHQ-1)]GACTTGGGACTCA
ID03 Deletion	Probe	GCAAATGAGATTTGGAGGACTGTGCATG[T(FAM)]GG[THF]T[T(BHQ-1)]GGGACTCATCATT
ID04 PCR Fwd	Primer	GACCCTAATCCAGTATGTTGCTG
ID04 PCR Rev	Primer	CCCTTCAGAGTGTCTAGAAGAGC
ID04 S	Primer	GACCCTAATCCAGTATGTTGCTG
ID04 RPA Fwd	Primer	GGAAATTTGGACACAGAGACAGATGTGCATA
ID04 RPA Rev	Primer	CCCTTCAGAGTGTCTAGAAGAGCATCCTTC
ID04 Insertion	Probe	GCAGTCCTAGTCCAGTCTCTCCCTTTG[T(FAM)]CT[THF]G[T(BHQ-1)]GAAGATATGATTTTC
ID04 Deletion	Probe	TTGTGGCAGTCCTAGTCCAGTCTCTCCC[T(FAM)]T[THF]GA[T(BHQ-1)]ATGATTTTCTTTGC
ID05 Fwd	Primer	TGGGATGACGACTTGGCTTC
ID05 Rev	Primer	ACCTCTGTCCAAGGGAGACA
ID05 S	Primer	TGGGATGACGACTTGGCTTC
ID05 RPA Fwd	Primer	TGTGGAGAAACAGGAGACATTTAATAGTCATGC
ID05 RPA Rev	Primer	TCTCAAAGTGCTGGGATTGCAGGCGTGAGC
ID05 Insertion	Probe	GTAACTGGCACACCCATTCCCTAATGTCG[T(FAM)][THF][T(BHQ-1)]CTATGAAAATGACC
ID05 Deletion	Probe	ACATGTAACTGGCACACCCATTCCCTAA[T(FAM)]G[THF]C[T(BHQ-1)]ATGAAAATGACCT
ID06 Fwd	Primer	CATGTAAAGAGTTGAGACATGGAC
ID06 Rev	Primer	GGCTGTGAACAGTTAGTAAGTG
ID06 S	Primer	CATGTAAAGAGTTGAGACATGGAC
ID06 RPA Fwd	Primer	CATGTAAAGAGTTGAGACATGGACAATGTT
ID06 RPA Rev	Primer	GGCTGTGAACAGTTAGTAAGTGGCATATGAT
ID06 Insertion	Probe	AGATGATGTTTCATTCATATATAGTTCTC[T(FAM)][THF]T[T(BHQ1)]GTCAGTAATCCTT
ID06 Deletion	Probe	CAAAAAGATGATGTTTCATTCATATATAG[T(FAM)]TC[THF]G[T(BHQ-1)]CAGTAATCCTTTA
ID07 PCR Fwd	Primer	TTTGTGGTTCTAAACATTAGCTAGT
ID07 PCR Rev	Primer	GAGAATCCTGCTTGCTAAACCA
ID07 S	Primer	TTTGTGGTTCTAAACATTAGCTAGT
ID07 RPA Fwd	Primer	GTATATAATAGTACATTTAATATTTGGAGTC
ID07 RPA Rev	Primer	TAAACCACCTGAGGACACATGCAATGATAATGA
ID07 Insertion	Probe	GCTTGGTGAACCAAAATCAGTGTTGAGTA[T(FAM)][THF]T[T(BHQ-1)]AAAATTAGCACAT
ID07 Deletion	Probe	GCTTGGTGAACCAAAATCAGTGTTGAG[T(FAM)]AA[THF]AT[T(BHQ-1)]AGCACATGGCCC

Table S3: Error rates as a function of probe sequence features. Error rates were calculated as the percentage incorrectly genotyped samples per locus. The included probe sequence features are GC content (%), melting temperature (Tm, °C), longest homopolymer stretch (poly-A/C/T/G), repetitive sequences, and insertion sequence.

Probe	Sequence	% Error	%GC	Tm (°C)	Poly-A/C/T/G	Repetitive sequences	Insertion length
Amel-I	GAATAGTGGGTGGATTCTTCATCCCAAA[T(FAM)]AA[THF]G[T(BHQ-1)]GGTTTCTCAAGTG	2.6	40.4	72.7	3	/	AAAGTG
Amel-D	GAATAGTGTGTTGATTCTTTATCCCAGA[T(FAM)]G[THF]T[T(BHQ-1)]CTCAAGTGGTCCT	2.6	39.1	72.4	3	TGTG	/
ID01-I	GCATTTCTATGCCATTTTCAGTTAATGTAT[T(FAM)]T[THF]G[T(BHQ-1)]TTCACCTGCAATAT	0	32.7	72.4	4	/	ATTTGGT
ID01-D	AATATGCATTTCTATGCCATTTTCAGTTAA[T(FAM)]G[THF]T[T(BHQ-1)]CACCTGCAATATTT	0	28.6	72.0	4	/	/
ID02-I	TGGGAAATGGCCAGGAACTCAGTAAGAG[T(FAM)]T[THF]G[T(BHQ-1)]CAAATGGGTAAT	7.9	41.3	74.2	3	AGAG	AGTTTG
ID02-D	TCTCCTTGGGAAATGGCCAGGAACTCAG[T(FAM)]A[THF]G[T(BHQ-1)]CAAATGGGTAATA	7.9	43.5	74.6	3	TCTC<br>GTAGTC	/
ID03-I	ATGAGATTTGGAGGACTGTGCATGTGGCC[T(FAM)][THF]C[T(BHQ-1)]GACTTGGGACTCA	0	50.0	76.8	3	GGAGGA<br>GAGA<br>TGTG	CTACTGAC
ID03-D	GCAAATGAGATTTGGAGGACTGTGCATG[T(FAM)]GG[THF]T[T(BHQ-1)]GGGACTCATCATT	0	46.8	76.1	3	GGAGGA<br>GAGA<br>TGTG	/
ID04-I	GCAGTCCTAGTCCAGTCTCTCCCTTTG[T(FAM)]CT[THF]G[T(BHQ-1)]GAAGATATGATTTTC	0	43.8	73.3	4	TCTCTC<br>GATATGAT	TTCACAA<br>GAC
ID04-D	TTGTGGCAGTCCTAGTCCAGTCTCTCCC[T(FAM)]T[THF]GA[T(BHQ-1)]ATGATTTTCTTTGC	0	43.8	74.5	4	TCTCTC<br>GATATGAT<br>TTTTCTTT	/

Probe	Sequence	% Error	%GC	Tm (°C)	Poly-A/C/T/G	Repetitive sequences	Insertion length
ID05-I	GTAACTGGCACACCCATTCCCTAATGTCG[T(FAM)][THF][T(BHQ-1)]CTATGAAAATGACC	0	45.7	74.1	4	CACA<br>GTGT	GTGTC
ID05-D	ACATGTAACTGGCACACCCATTCCCTAA[T(FAM)]G[THF]C[T(BHQ-1)]ATGAAAATGACCT	0	41.3	74.2	4	CACA	/
ID06-I	AGATGATGTTTCATTCATATATAGTTCTC[T(FAM)][THF]T[T(BHQ1)]GTCAGTAATCCTT	10.3	28.3	69.3	4	GATGATGTT<br>CATTCAT<br>ATATATAG<br>TCTCTT	CTTTT
ID06-D	CAAAAAGATGATGTTTCATTCATATATAG[T(FAM)]TC[THF]G[T(BHQ-1)]CAGTAATCCTTTA	10.3	27.1	69	5	GATGATGTT<br>CATTCAT<br>ATATATAG<br>TCTGTC	/
ID07-I	GCTTGGTGAACCAAAATCAGTGTTGAGTA[T(FAM)][THF]T[T(BHQ-1)]AAAATTAGCACAT	2.6	34.8	72	4	GTGT<br>CACA	TCTTA
ID07-D	GCTTGGTGAACCAAAATCAGTGTTGAG[T(FAM)]AA[THF]AT[T(BHQ-1)]AGCACATGGCCC	2.6	43.5	74.4	4	GTGT<br>CACA	/

Figure S1: Sanger sequencing electropherograms showing the true genotype of Sample 1-9 for all eight loci included in the InDel panel. Locus name and insertion sequence are given above each table, with locus names given as 'ID code (dbSNP RefSNP number)'. +,+ = homozygous insertion; -,- = homozygous deletion; +/- = heterozygous. The InDel allele is indicated with a black box.

Amelogenin
Insertion: AAAGTG

Sample	True genotype (Amel)	Sanger sequencing chromatogram
Sample 1	XX	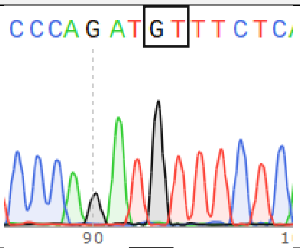
Sample 2	XX	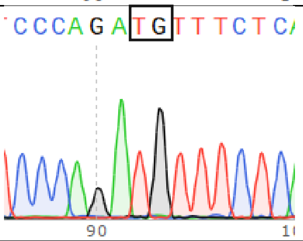
Sample 3	XY	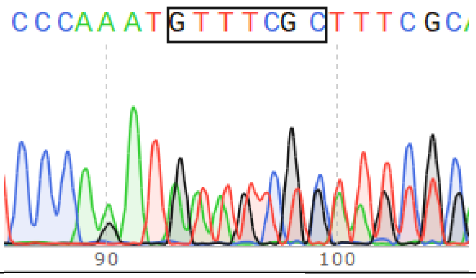
Sample 4	XX	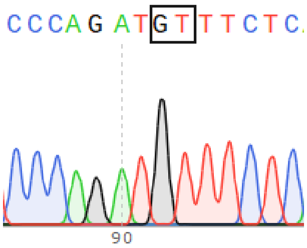
Sample 5	XY	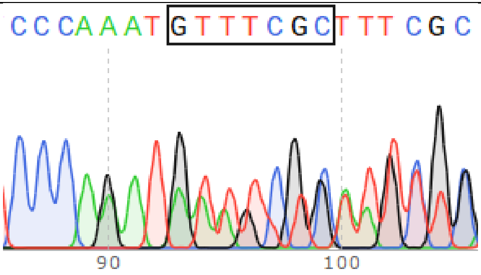

Sample 6	XY	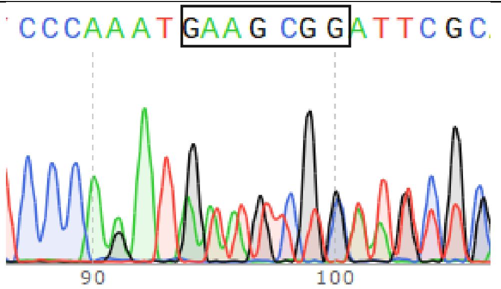
Sample 7	XY	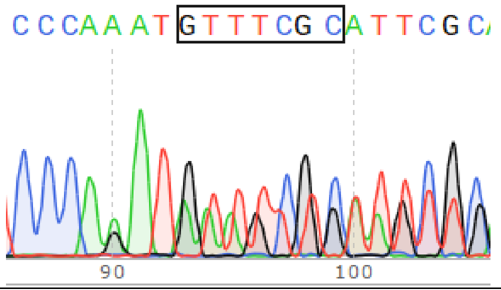
Sample 8	XX	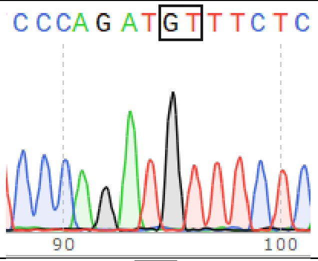
Sample 9	XX	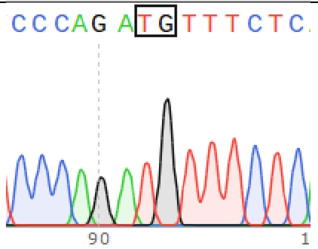

Locus ID01 (rs57943214)
Insertion: ATTTGGT

Sample	True genotype (ID01)	Sanger sequencing chromatogram
Sample 1	-,-	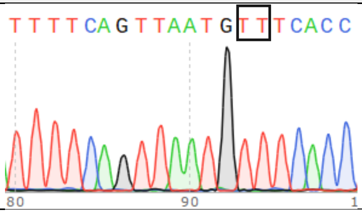
Sample 2	+,-	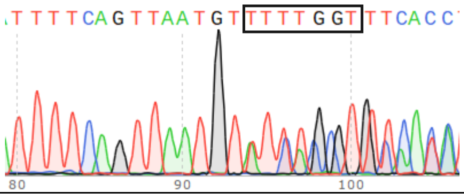

Sample 3	-,-	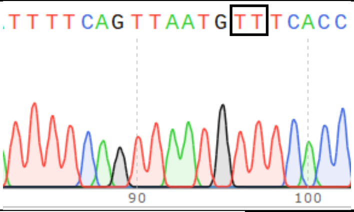
Sample 4	+,+	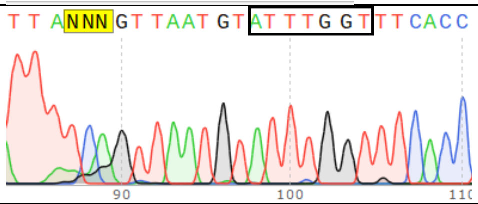
Sample 5	-,-	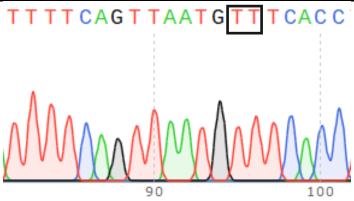
Sample 6	+,-	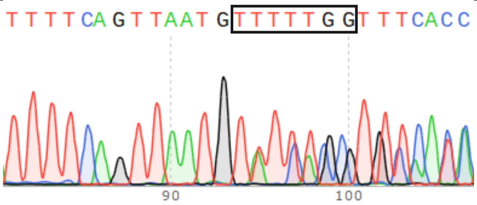
Sample 7	+,-	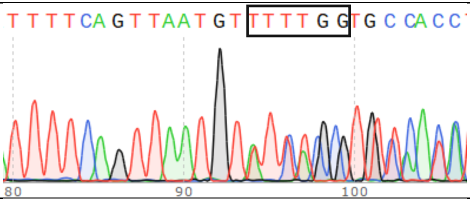
Sample 8	+,+	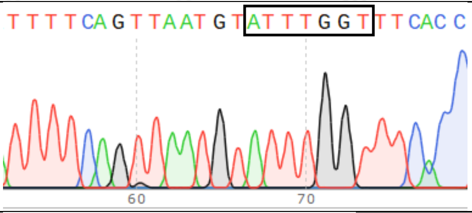
Sample 9	-,-	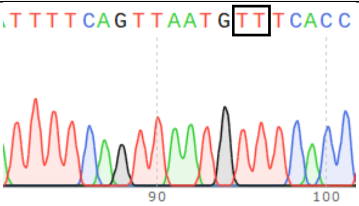

Locus ID02 (rs67320356)
Insertion: AGTTTG

Sample	True genotype (ID02)	Sanger sequencing chromatogram
Sample 1	+,-	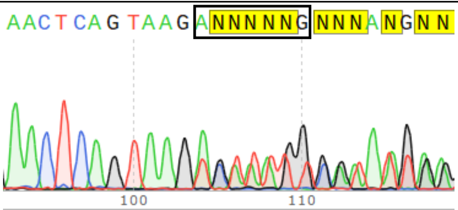
Sample 2	+,+	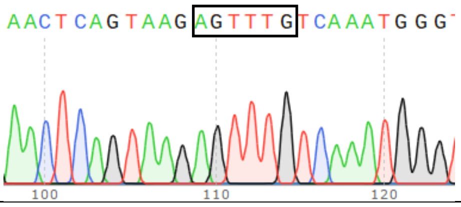
Sample 3	+,+	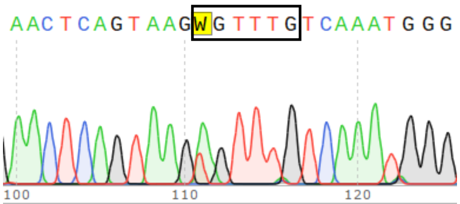
Sample 4	+,+	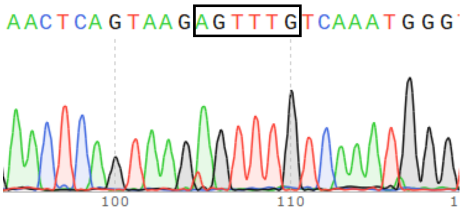
Sample 5	+,+	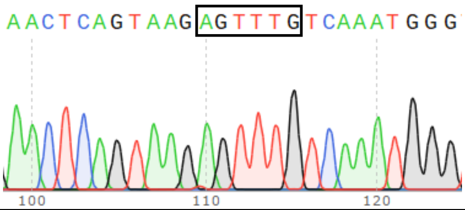
Sample 6	-,-	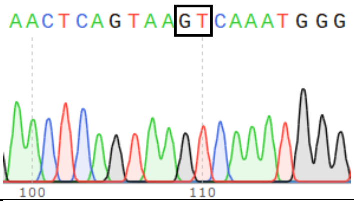
Sample 7	+,+	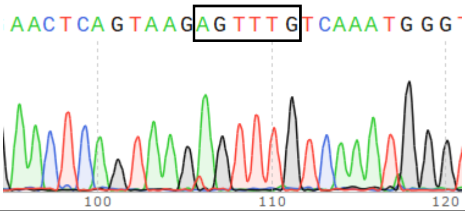

Sample 8	+,-	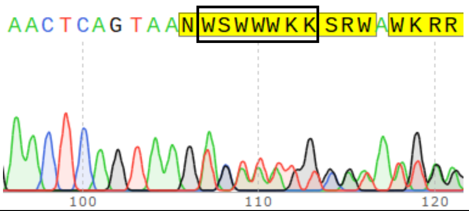
Sample 9	+,-	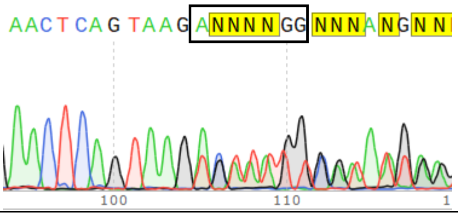

Locus ID03 (rs11281372)
Insertion: CCTACTGA

Sample	True genotype (ID03)	Sanger sequencing chromatogram
Sample 1	-,-	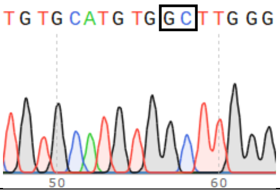
Sample 2	+,+	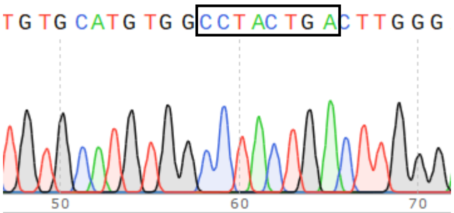
Sample 3	+,-	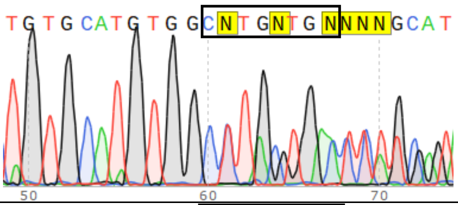
Sample 4	+,+	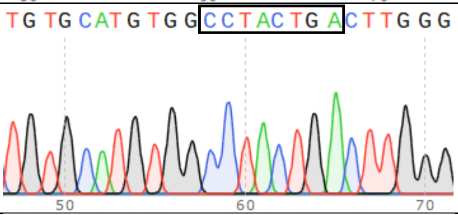
Sample 5	+,-	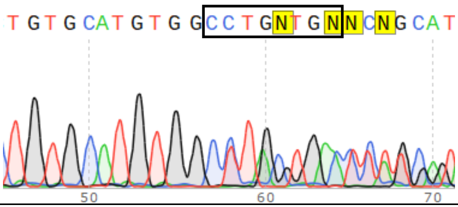

Sample 6	-,-	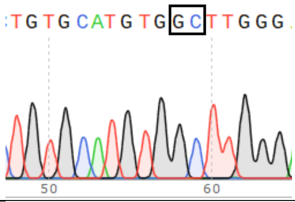
Sample 7	+,-	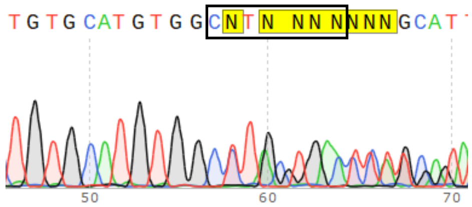
Sample 8	+,+	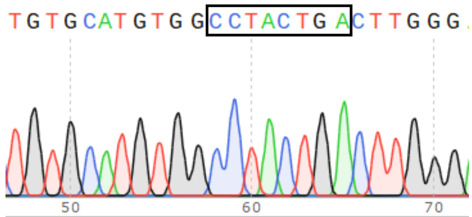
Sample 9	+,-	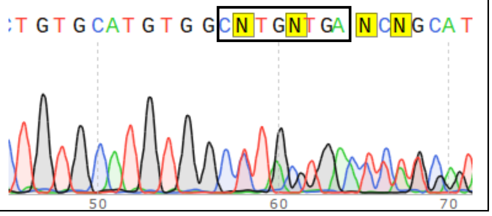

Locus ID04 (rs34159280)
Insertion: TTCACAAGAC

Sample	True genotype (ID04)	Sanger sequencing chromatogram
Sample 1	+,+	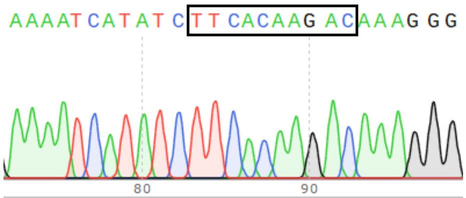
Sample 2	+,+	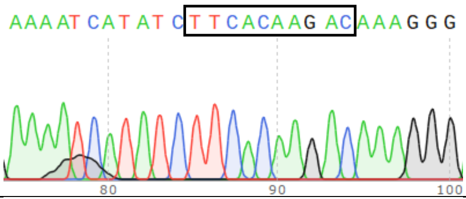
Sample 3	+,-	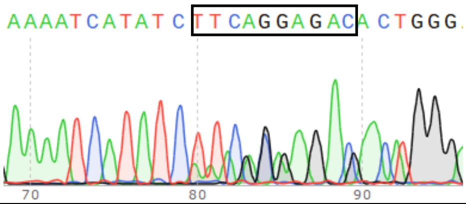

Sample 4	+,-	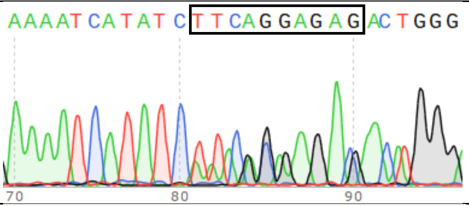
Sample 5	+,+	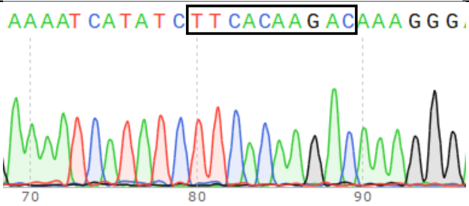
Sample 6	+,+	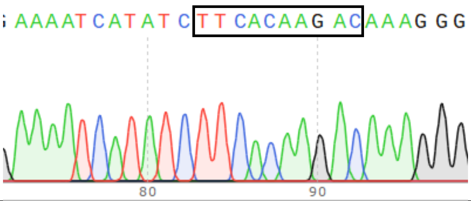
Sample 7	+,-	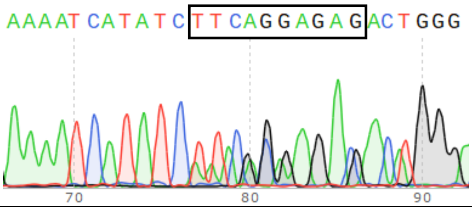
Sample 8	-,-	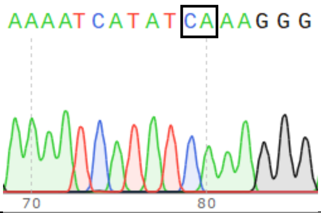
Sample 9	+,+	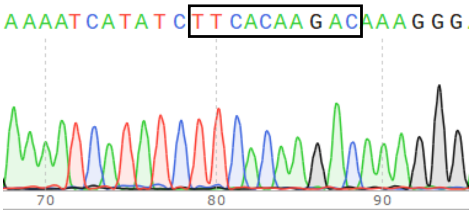

Locus ID05 (rs58092599)
Insertion: GTGTC

Sample	True genotype (ID05)	Sanger sequencing chromatogram
Sample 1	-,-	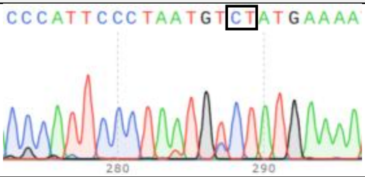
Sample 2	+,-	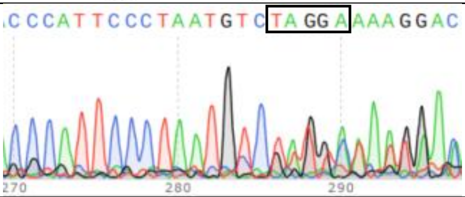

Sample 3	+,-	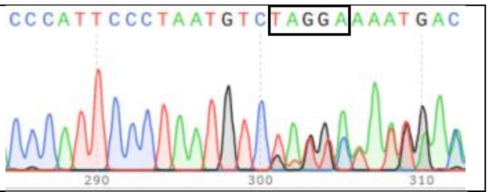
Sample 4	+,-	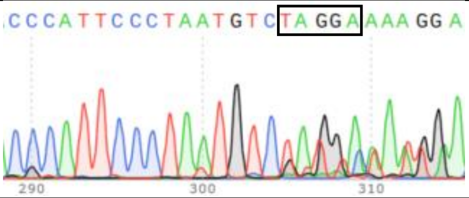
Sample 5	+,-	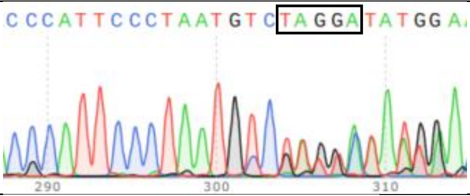
Sample 6	-,-	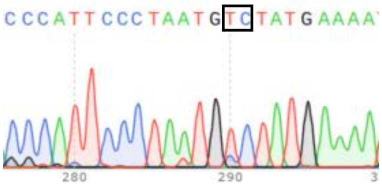
Sample 7	+,-	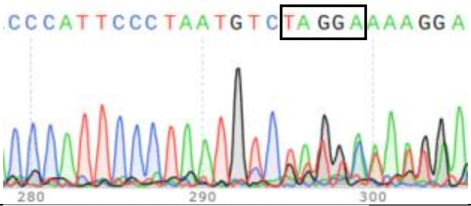
Sample 8	-,-	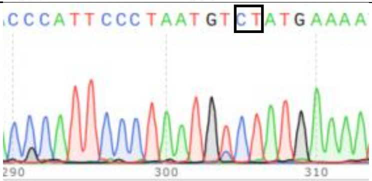
Sample 9	+,-	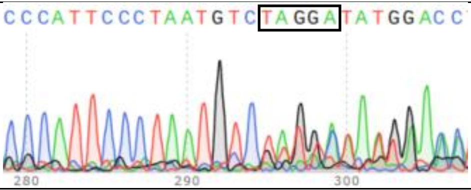

Locus ID06 (rs66739142)
Insertion: CTTTT

Sample	True genotype (ID06)	Sanger sequencing chromatogram
Sample 1	+,+	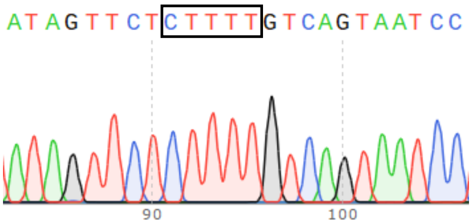

Sample 2	+,-	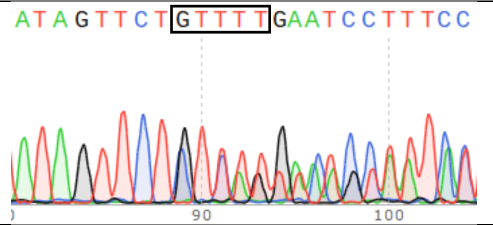
Sample 3	+,-	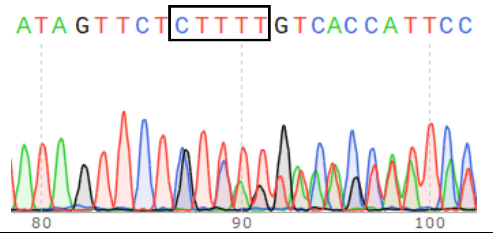
Sample 4	+,+	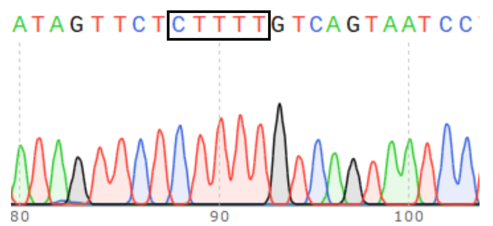
Sample 5	+,-	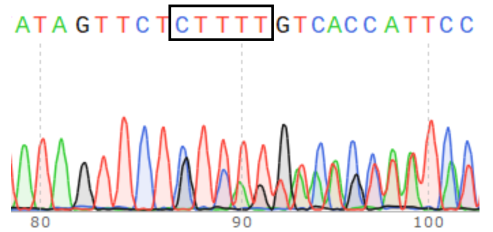
Sample 6	+,+	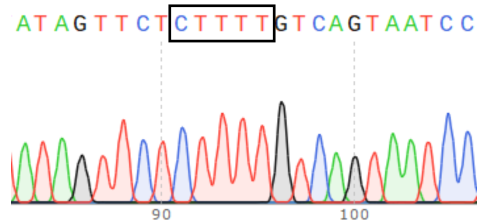
Sample 7	+,+	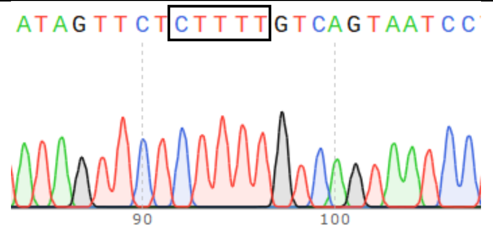
Sample 8	+,+	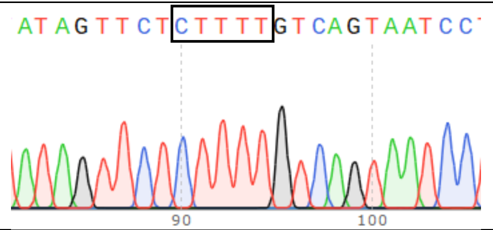
Sample 9	+,-	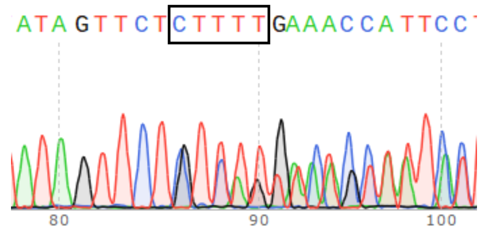

Locus ID07 (rs3067397)
Insertion: TCTTA

Sample	True genotype (ID07)	Sanger sequencing chromatogram
Sample 1	+,-	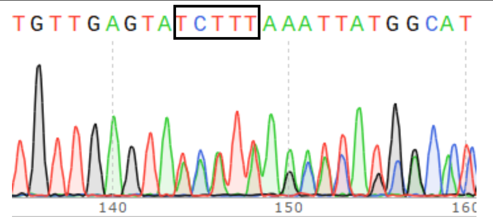
Sample 2	+,-	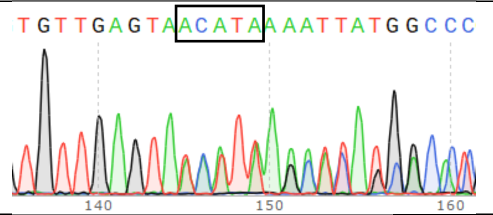
Sample 3	-,-	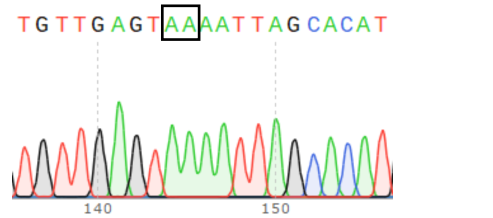
Sample 4	-,-	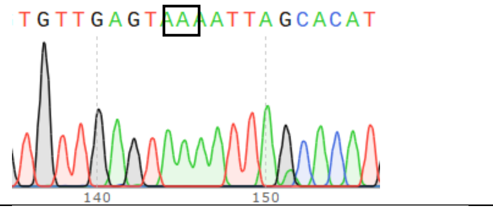
Sample 5	+,-	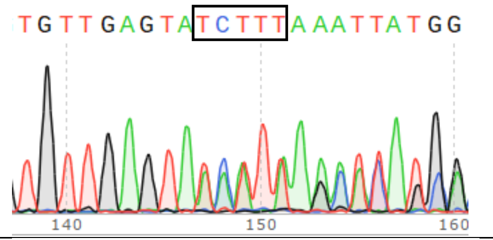
Sample 6	+,-	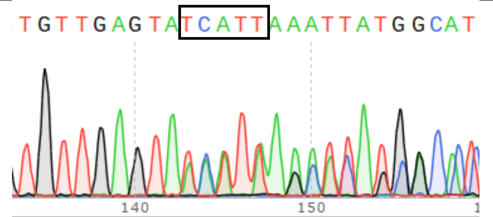
Sample 7	+,-	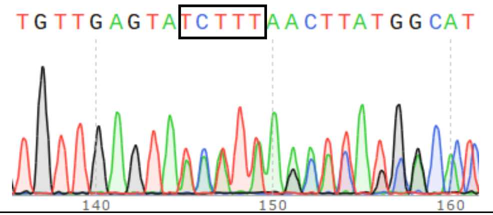

Sample 8	-,-	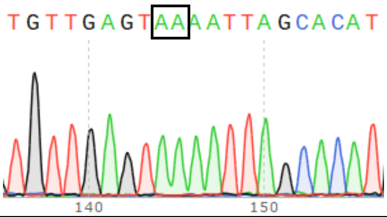
Sample 9	+,-	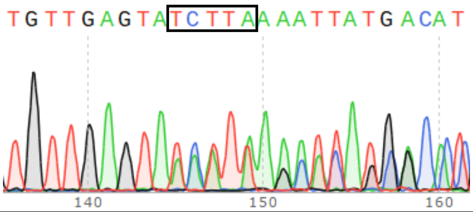

Figure S2: Real-time RPA graphs of all loci obtained for 3 NTC samples in triplicate. Locus number is indicated above each graph. All curves are indicated with dashed lines, as none of the curves reached the 1000 unit threshold for sample inclusion.

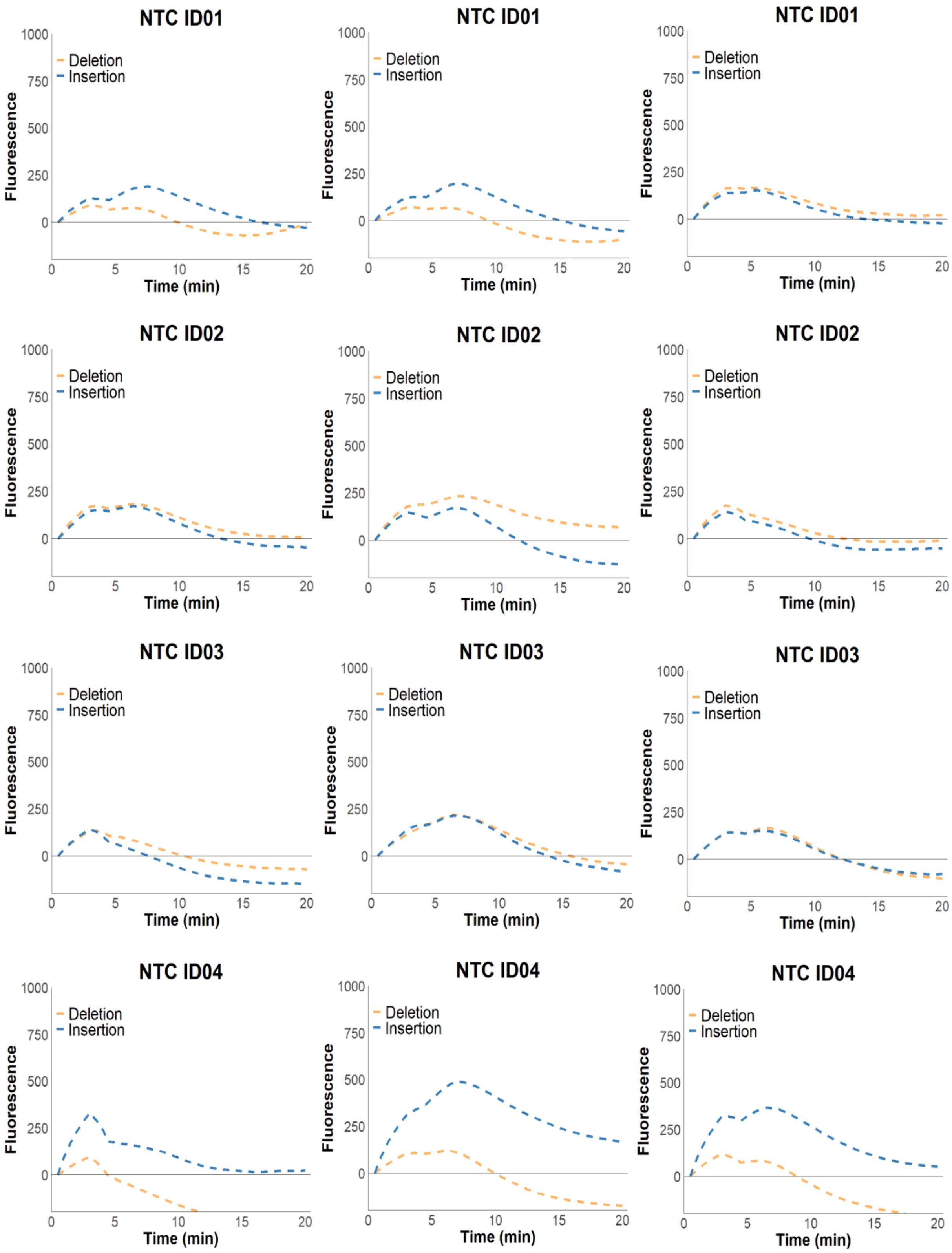

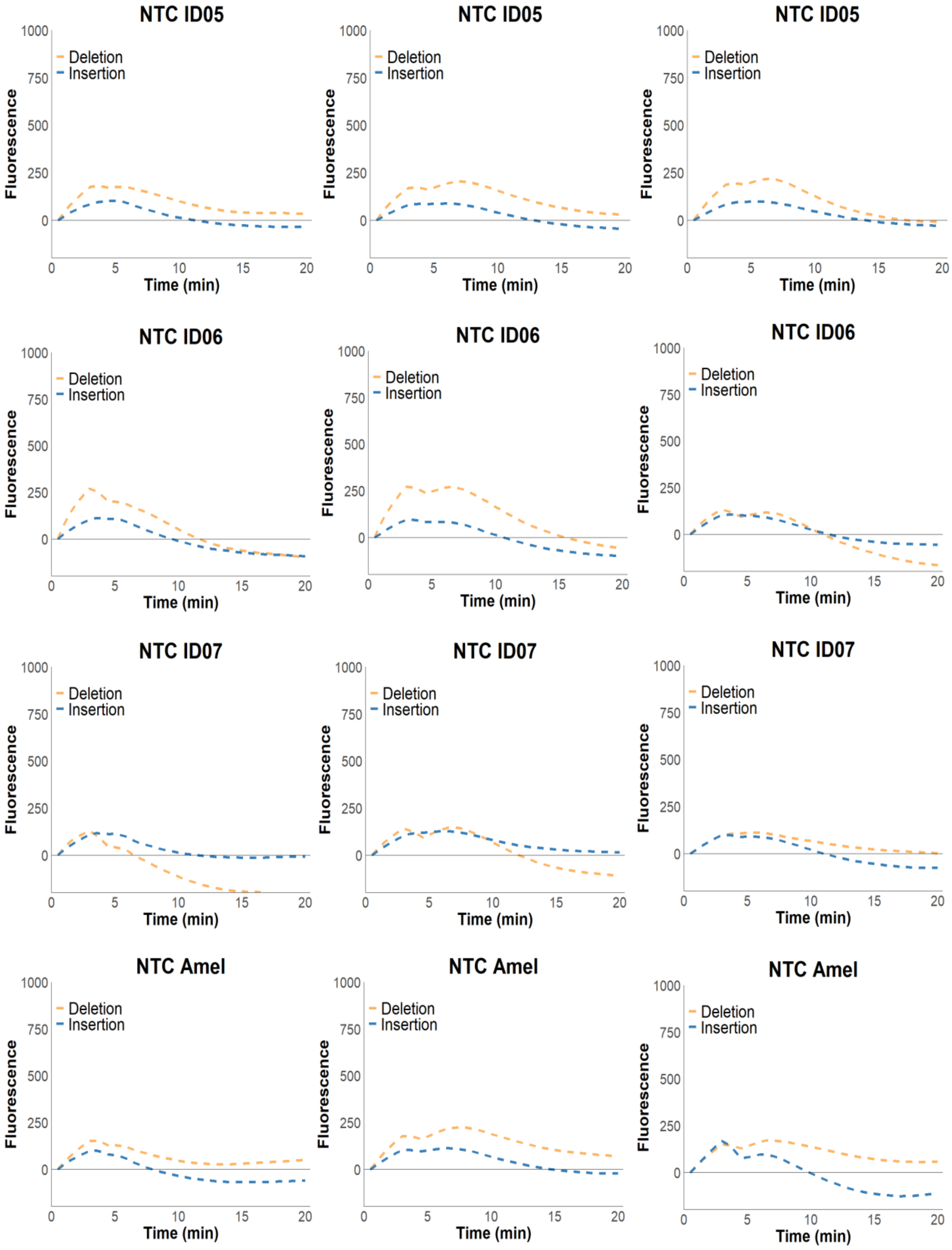
NTC ID05
Deletion
Insertion
Fluorescence
Time (min)
NTC ID05
Deletion
Insertion
Fluorescence
Time (min)
NTC ID05
Deletion
Insertion
Fluorescence
Time (min)
NTC ID06
Deletion
Insertion
Fluorescence
Time (min)
NTC ID06
Deletion
Insertion
Fluorescence
Time (min)
NTC ID06
Deletion
Insertion
Fluorescence
Time (min)
NTC ID07
Deletion
Insertion
Fluorescence
Time (min)
NTC ID07
Deletion
Insertion
Fluorescence
Time (min)
NTC ID07
Deletion
Insertion
Fluorescence
Time (min)
NTC Amel
Deletion
Insertion
Fluorescence
Time (min)
NTC Amel
Deletion
Insertion
Fluorescence
Time (min)
NTC Amel
Deletion
Insertion
Fluorescence
Time (min)

Figure S3: Drop-in and drop-out rates for as a function of potential thresholds, as calculated at each locus for all included samples in triplicate.

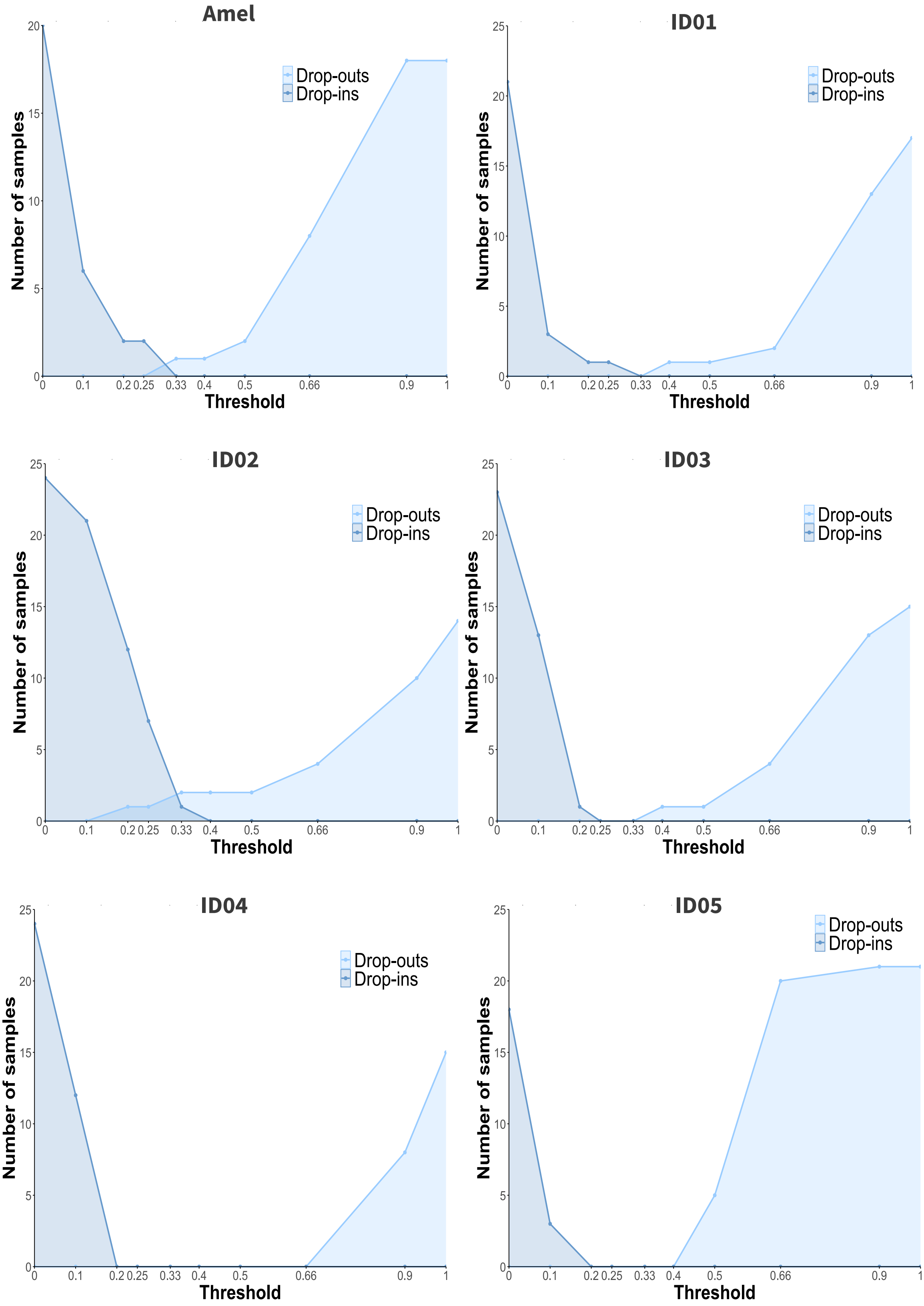

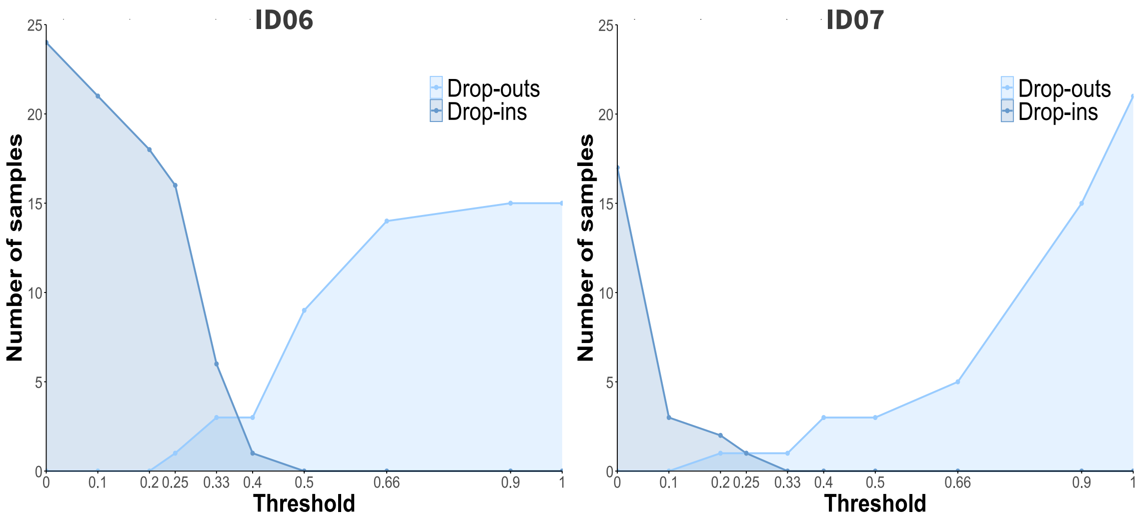
ID06
Drop-outs
Drop-ins
Number of samples
0
5
10
15
20
25
0
0.1
0.2
0.25
0.33
0.4
0.5
0.66
0.9
1
Threshold
ID07
Drop-outs
Drop-ins
Number of samples
Threshold

Figure S4: Real-time RPA graphs of locus Amelogenin obtained for all 12 samples in triplicate. Sample number and true genotype are indicated above each graph. Curves of alleles called as present are shown as full lines, while curves of not called alleles are shown as dashed lines. The horizontal grey line represents the genotyping threshold to call heterozygous samples at 1/3 of the endpoint value of the major curve.

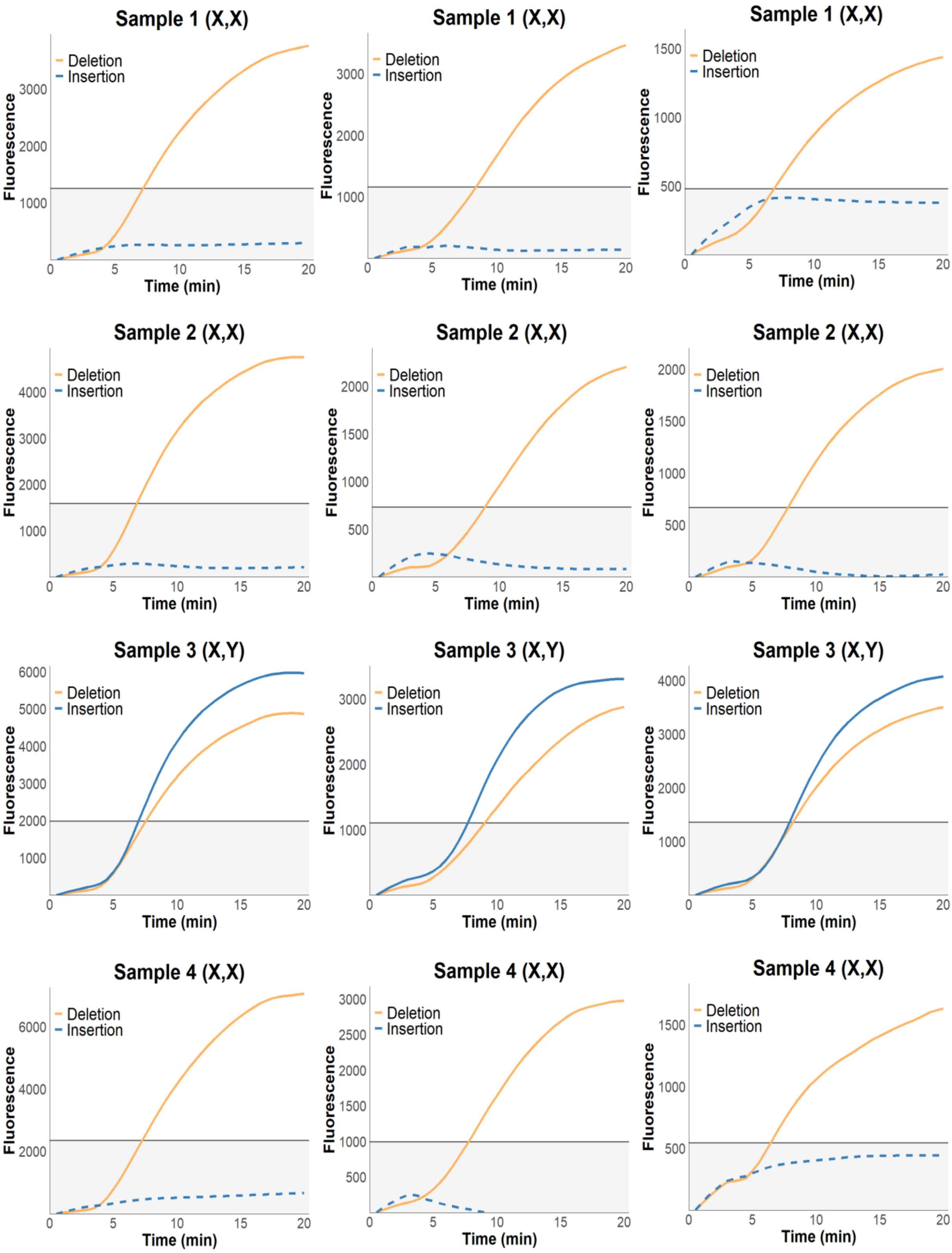

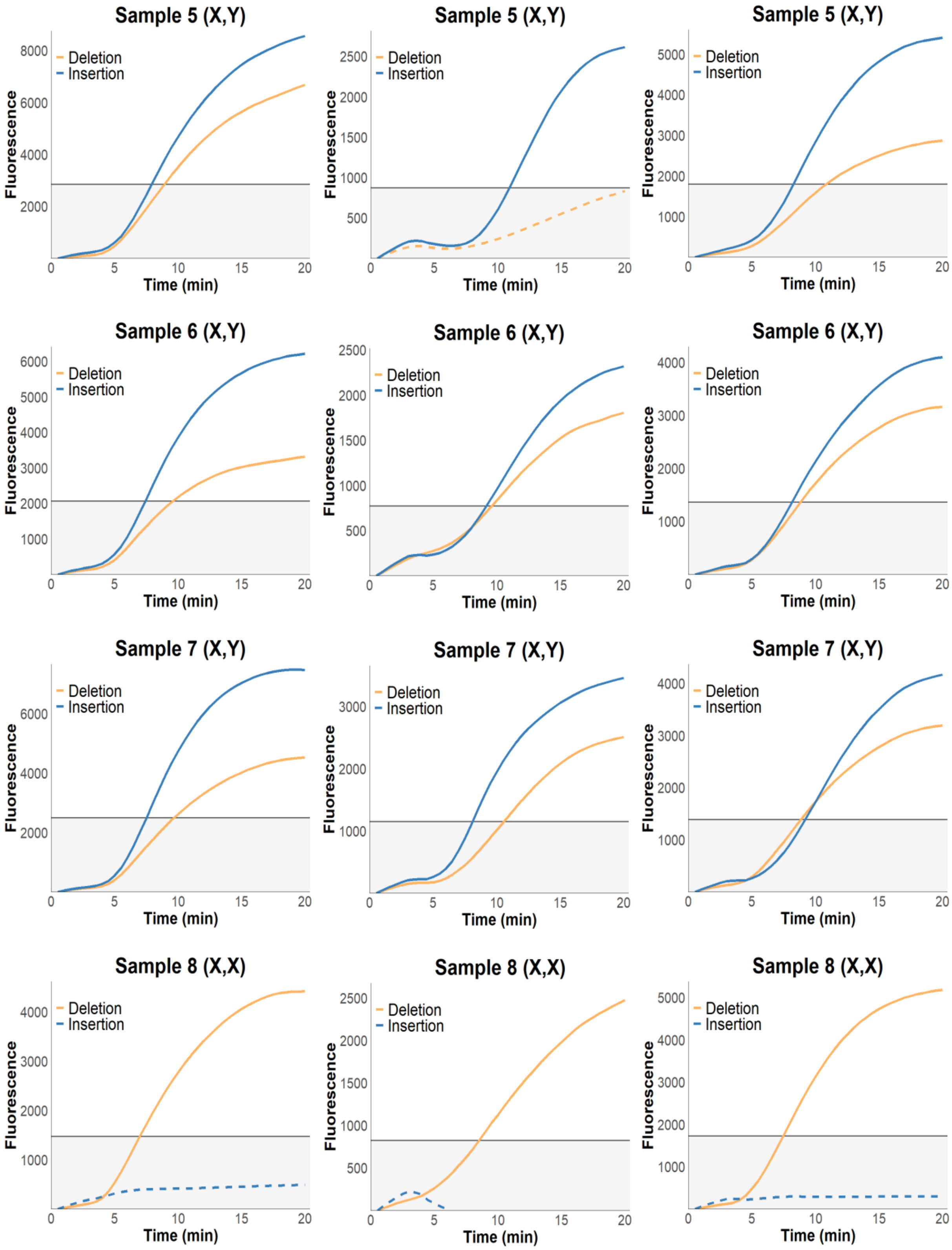

Sample 5 (X,Y)
Deletion
Insertion
Fluorescence
Time (min)
Sample 5 (X,Y)
Deletion
Insertion
Fluorescence
Time (min)
Sample 5 (X,Y)
Deletion
Insertion
Fluorescence
Time (min)
Sample 6 (X,Y)
Deletion
Insertion
Fluorescence
Time (min)
Sample 6 (X,Y)
Deletion
Insertion
Fluorescence
Time (min)
Sample 6 (X,Y)
Deletion
Insertion
Fluorescence
Time (min)
Sample 7 (X,Y)
Deletion
Insertion
Fluorescence
Time (min)
Sample 7 (X,Y)
Deletion
Insertion
Fluorescence
Time (min)
Sample 7 (X,Y)
Deletion
Insertion
Fluorescence
Time (min)
Sample 8 (X,X)
Deletion
Insertion
Fluorescence
Time (min)
Sample 8 (X,X)
Deletion
Insertion
Fluorescence
Time (min)
Sample 8 (X,X)
Deletion
Insertion
Fluorescence
Time (min)

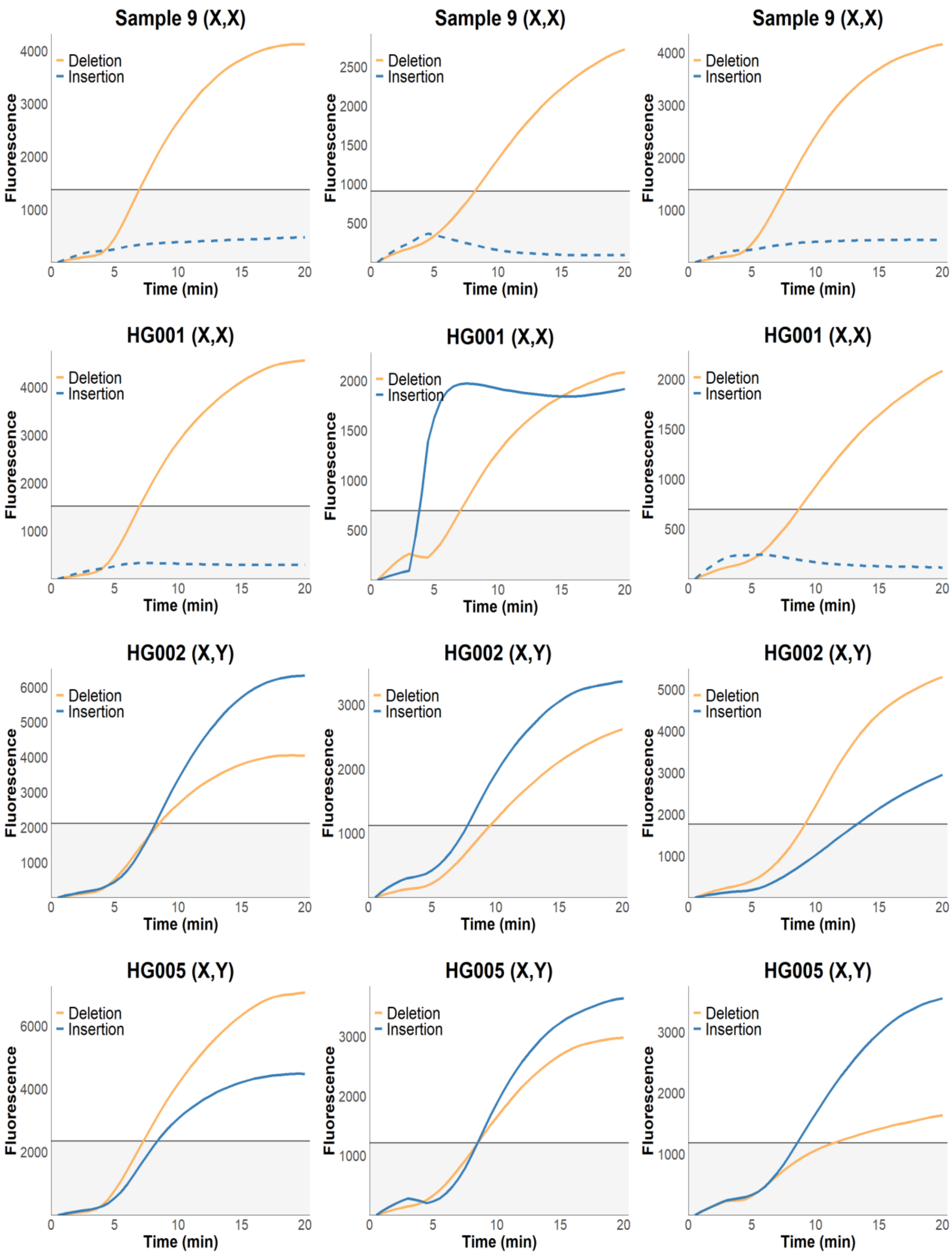
Sample 9 (X,X)
Sample 9 (X,X)
Sample 9 (X,X)
HG001 (X,X)
HG001 (X,X)
HG001 (X,X)
HG002 (X,Y)
HG002 (X,Y)
HG002 (X,Y)
HG005 (X,Y)
HG005 (X,Y)
HG005 (X,Y)
Deletion
Insertion
Fluorescence
Time (min)

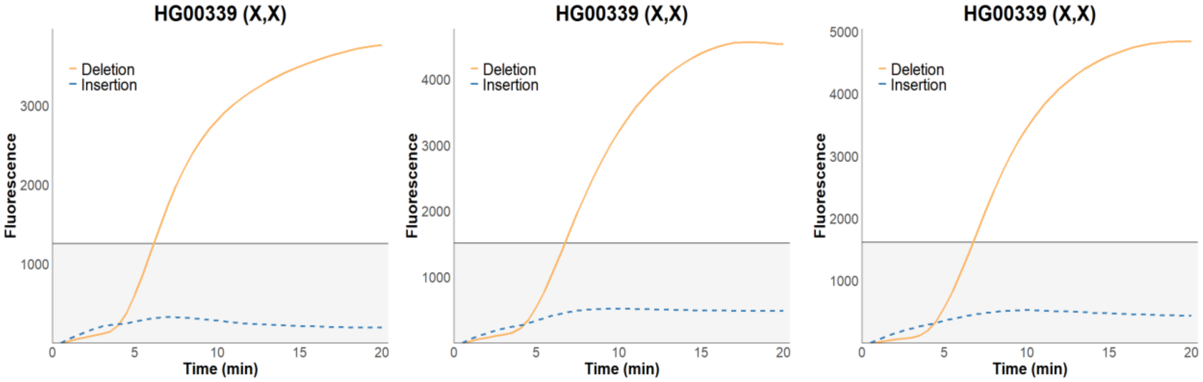
HG00339 (X,X)
Deletion
Insertion
Fluorescence
3000
2000
1000
0
5
10
15
20
Time (min)
HG00339 (X,X)
Deletion
Insertion
Fluorescence
4000
3000
2000
1000
0
5
10
15
20
Time (min)
HG00339 (X,X)
Deletion
Insertion
Fluorescence
5000
4000
3000
2000
1000
0
5
10
15
20
Time (min)

<u>Figure S5:</u> Real-time RPA graphs of locus ID01 obtained for all 12 samples in triplicate. Sample number and true genotype are indicated above each graph. Curves of alleles called as present are shown as full lines, while curves of not called alleles are shown as dashed lines. The horizontal grey line represents the genotyping threshold to call heterozygous samples at 1/3 of the endpoint value of the major curve.

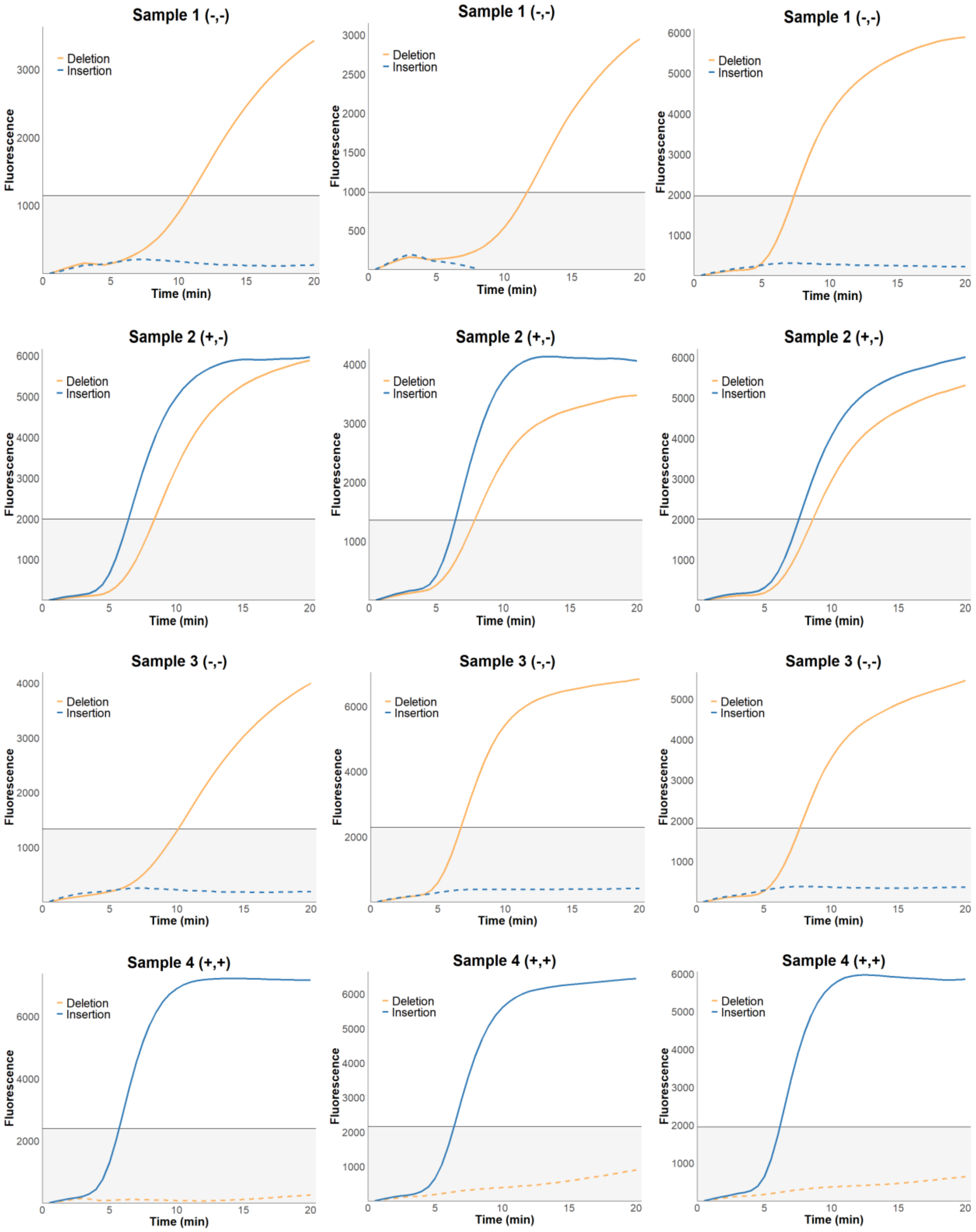

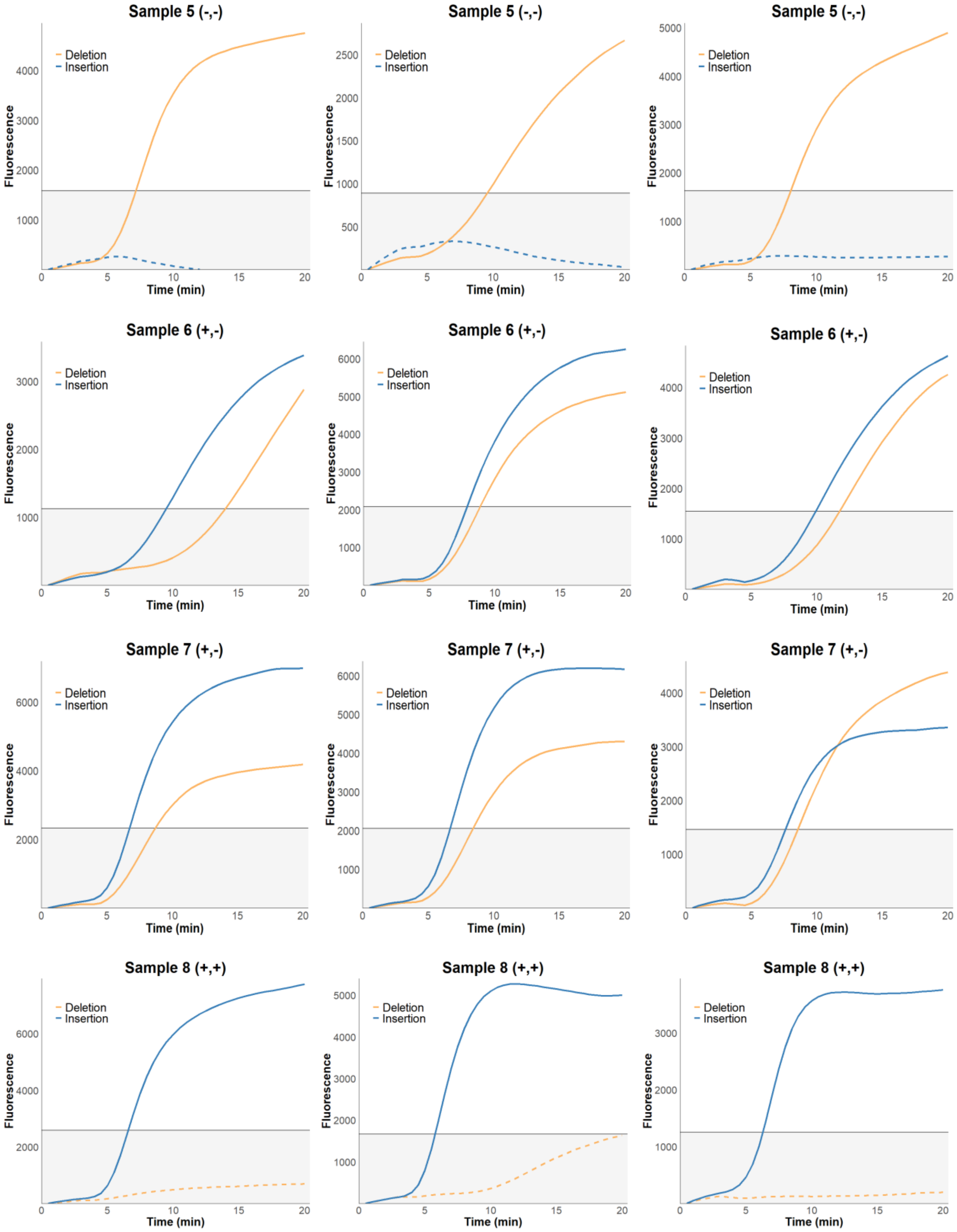
Sample 5 (-,-)
Sample 5 (-,-)
Sample 5 (-,-)
Sample 6 (+,-)
Sample 6 (+,-)
Sample 6 (+,-)
Sample 7 (+,-)
Sample 7 (+,-)
Sample 7 (+,-)
Sample 8 (+,+)
Sample 8 (+,+)
Sample 8 (+,+)
Deletion
Insertion
Fluorescence
Time (min)

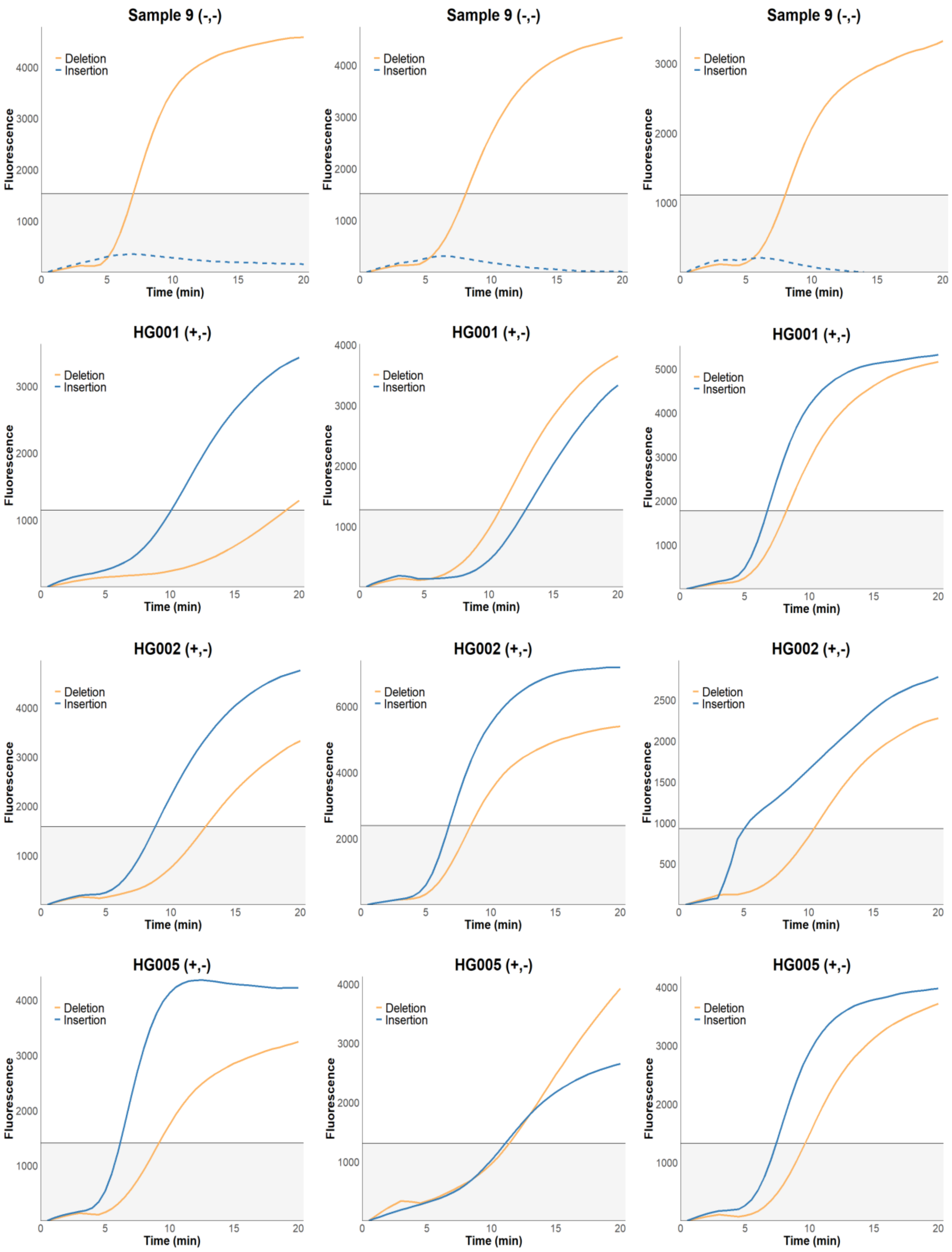
Sample 9 (-,-)
Deletion
Insertion
Fluorescence
Time (min)
Sample 9 (-,-)
Deletion
Insertion
Fluorescence
Time (min)
Sample 9 (-,-)
Deletion
Insertion
Fluorescence
Time (min)
HG001 (+,-)
Deletion
Insertion
Fluorescence
Time (min)
HG001 (+,-)
Deletion
Insertion
Fluorescence
Time (min)
HG001 (+,-)
Deletion
Insertion
Fluorescence
Time (min)
HG002 (+,-)
Deletion
Insertion
Fluorescence
Time (min)
HG002 (+,-)
Deletion
Insertion
Fluorescence
Time (min)
HG002 (+,-)
Deletion
Insertion
Fluorescence
Time (min)
HG005 (+,-)
Deletion
Insertion
Fluorescence
Time (min)
HG005 (+,-)
Deletion
Insertion
Fluorescence
Time (min)
HG005 (+,-)
Deletion
Insertion
Fluorescence
Time (min)

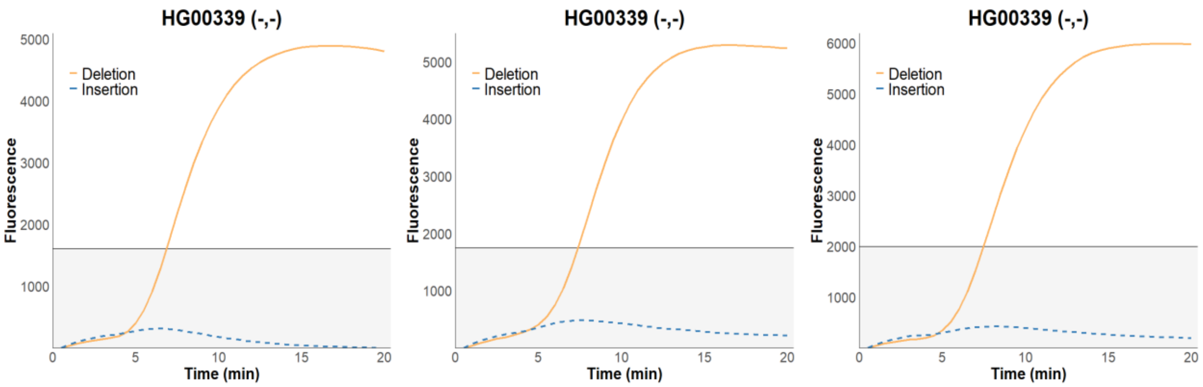
HG00339 (-,-)
Deletion
Insertion
Fluorescence
Time (min)
HG00339 (-,-)
Deletion
Insertion
Fluorescence
Time (min)
HG00339 (-,-)
Deletion
Insertion
Fluorescence
Time (min)

Figure S6: Real-time RPA graphs of locus ID02 obtained for all 12 samples in triplicate. Sample number and true genotype are indicated above each graph. Curves of alleles called as present are shown as full lines, while curves of not called alleles are shown as dashed lines. The horizontal grey line represents the genotyping threshold to call heterozygous samples at 1/3 of the endpoint value of the major curve.

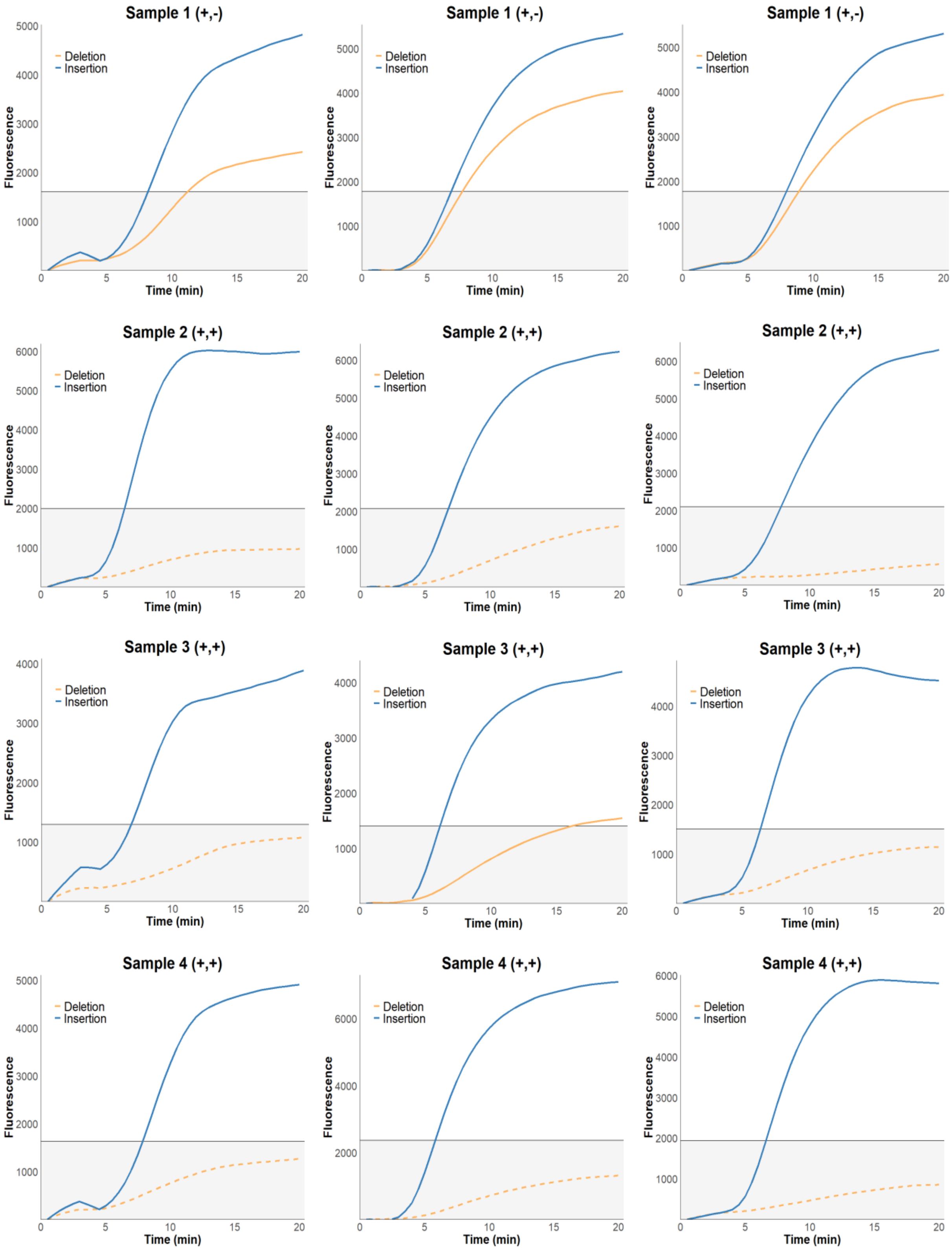

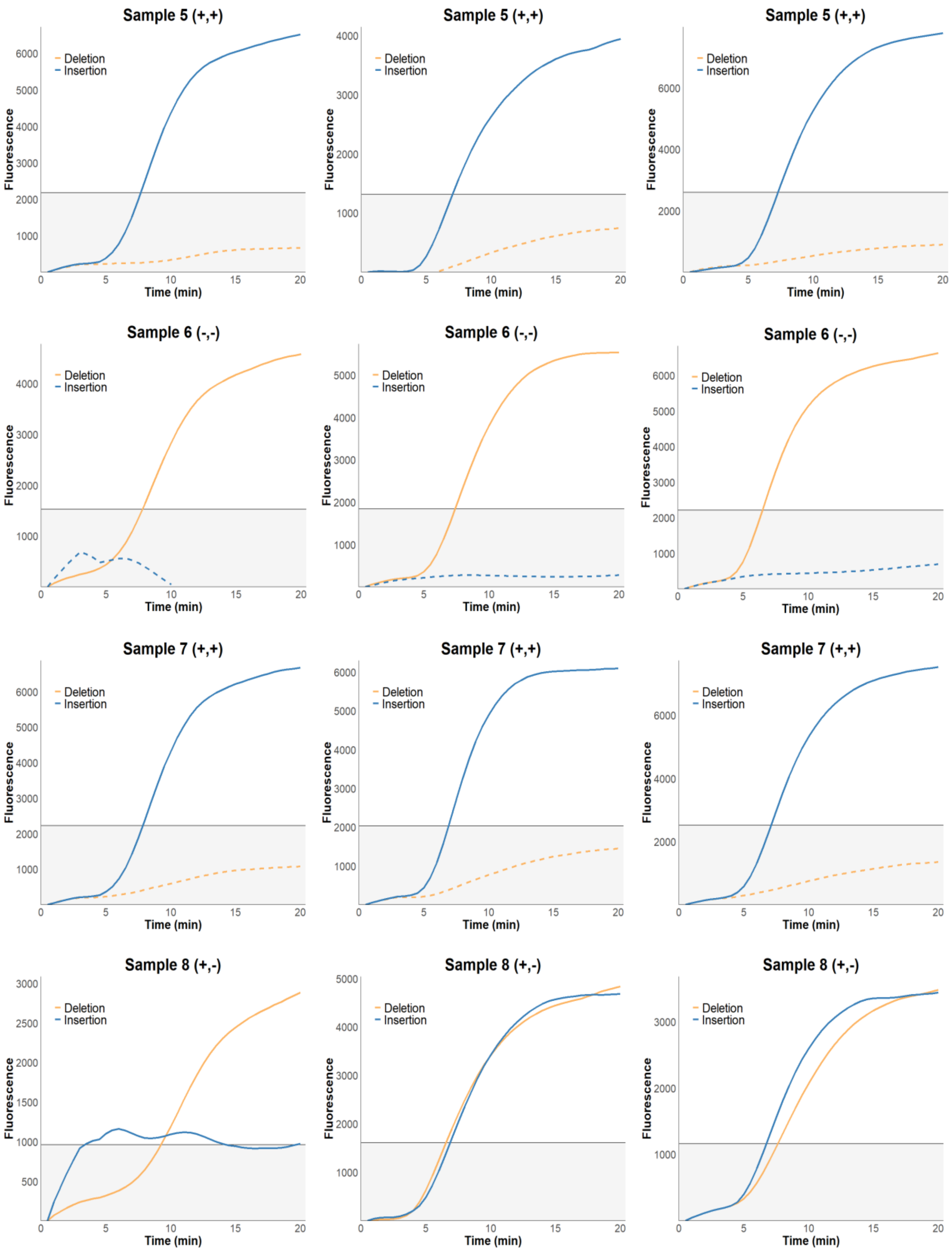

Sample 5 (+,+)
Sample 5 (+,+)
Sample 5 (+,+)
Sample 6 (-,-)
Sample 6 (-,-)
Sample 6 (-,-)
Sample 7 (+,+)
Sample 7 (+,+)
Sample 7 (+,+)
Sample 8 (+,-)
Sample 8 (+,-)
Sample 8 (+,-)
Deletion
Insertion
Fluorescence
Time (min)

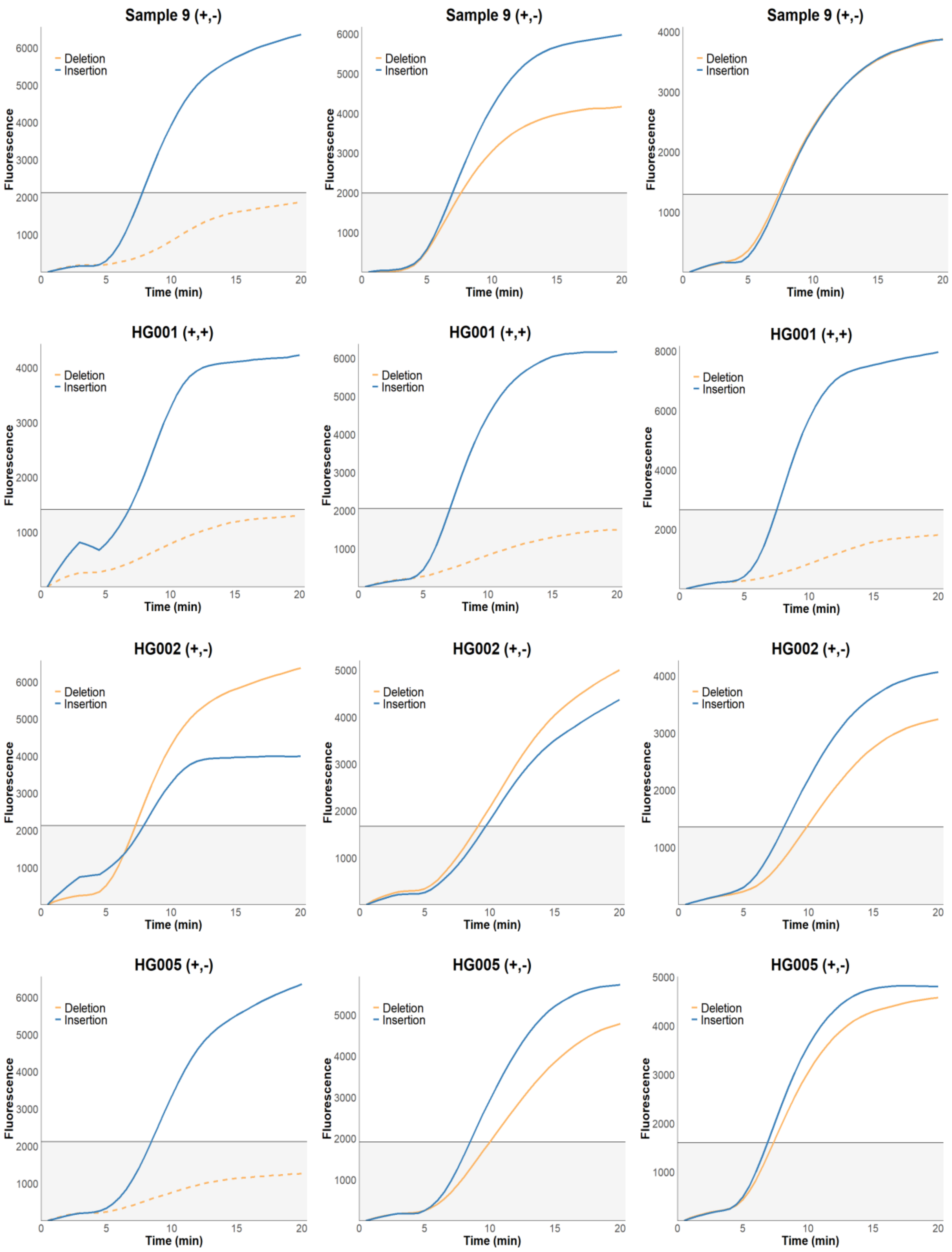
Sample 9 (+,-)
Sample 9 (+,-)
Sample 9 (+,-)
HG001 (+,+)
HG001 (+,+)
HG001 (+,+)
HG002 (+,-)
HG002 (+,-)
HG002 (+,-)
HG005 (+,-)
HG005 (+,-)
HG005 (+,-)
Deletion
Insertion
Fluorescence
Time (min)

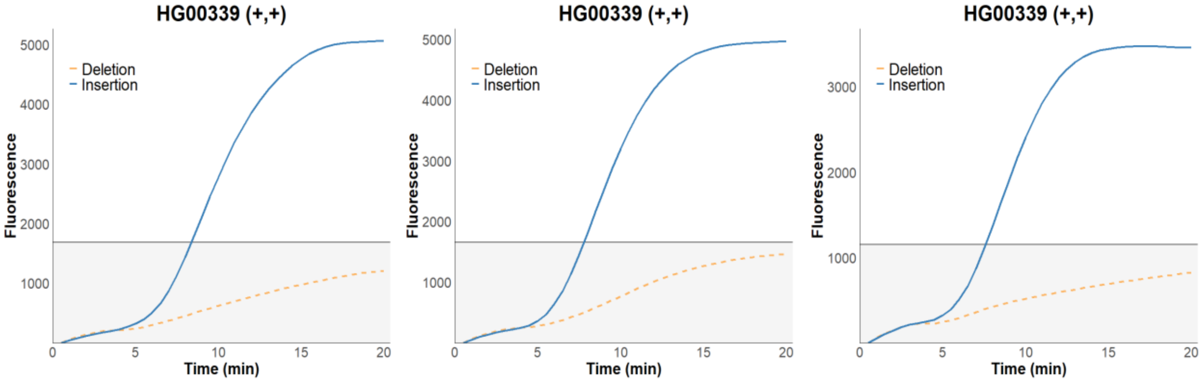

HG00339 (+,+)
Deletion
Insertion
Fluorescence
Time (min)
HG00339 (+,+)
Deletion
Insertion
Fluorescence
Time (min)
HG00339 (+,+)
Deletion
Insertion
Fluorescence
Time (min)

<u>Figure S7:</u> Real-time RPA graphs of locus ID03 obtained for all 12 samples in triplicate. Sample number and true genotype are indicated above each graph. Curves of alleles called as present are shown as full lines, while curves of not called alleles are shown as dashed lines. The horizontal grey line represents the genotyping threshold to call heterozygous samples at 1/3 of the endpoint value of the major curve.

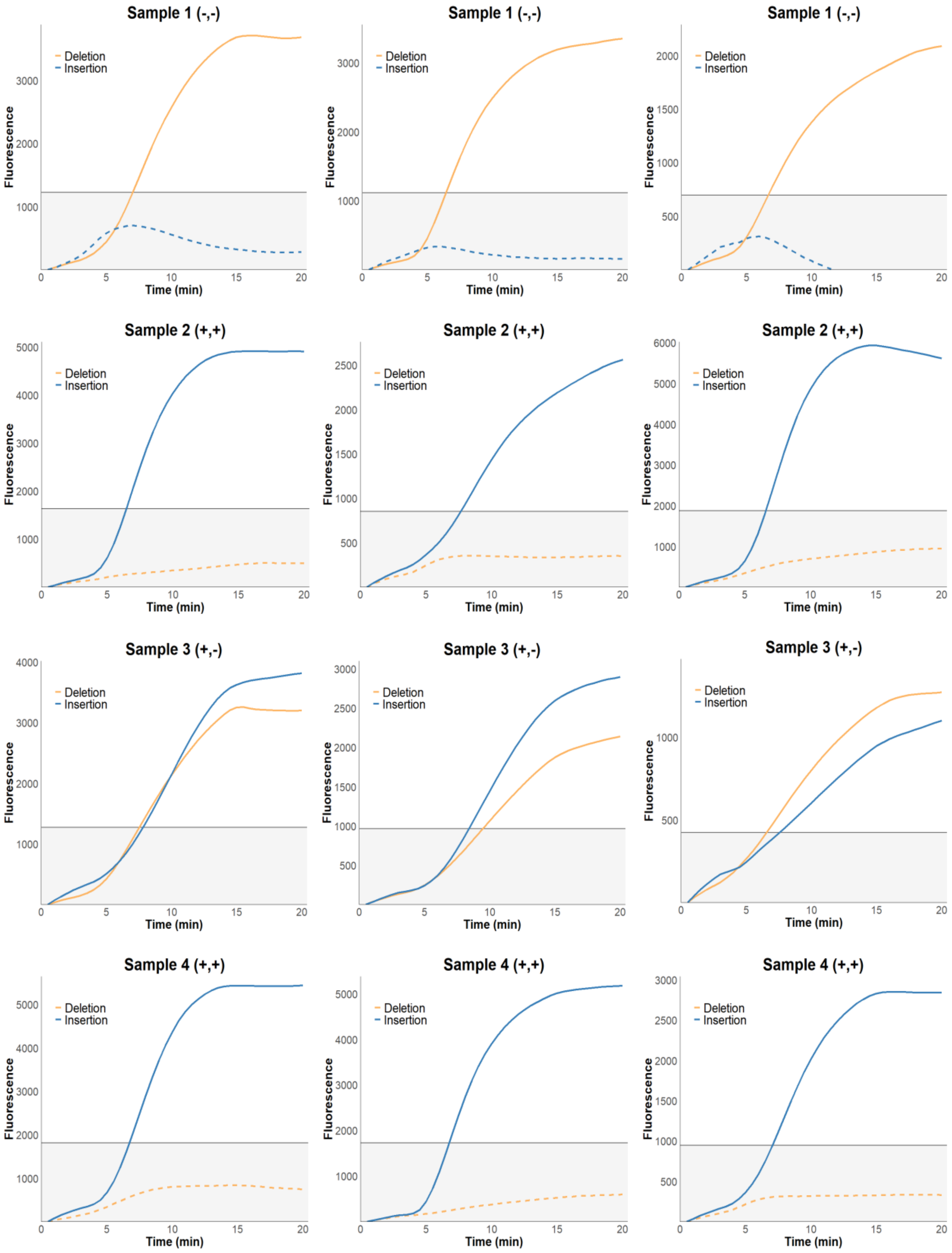

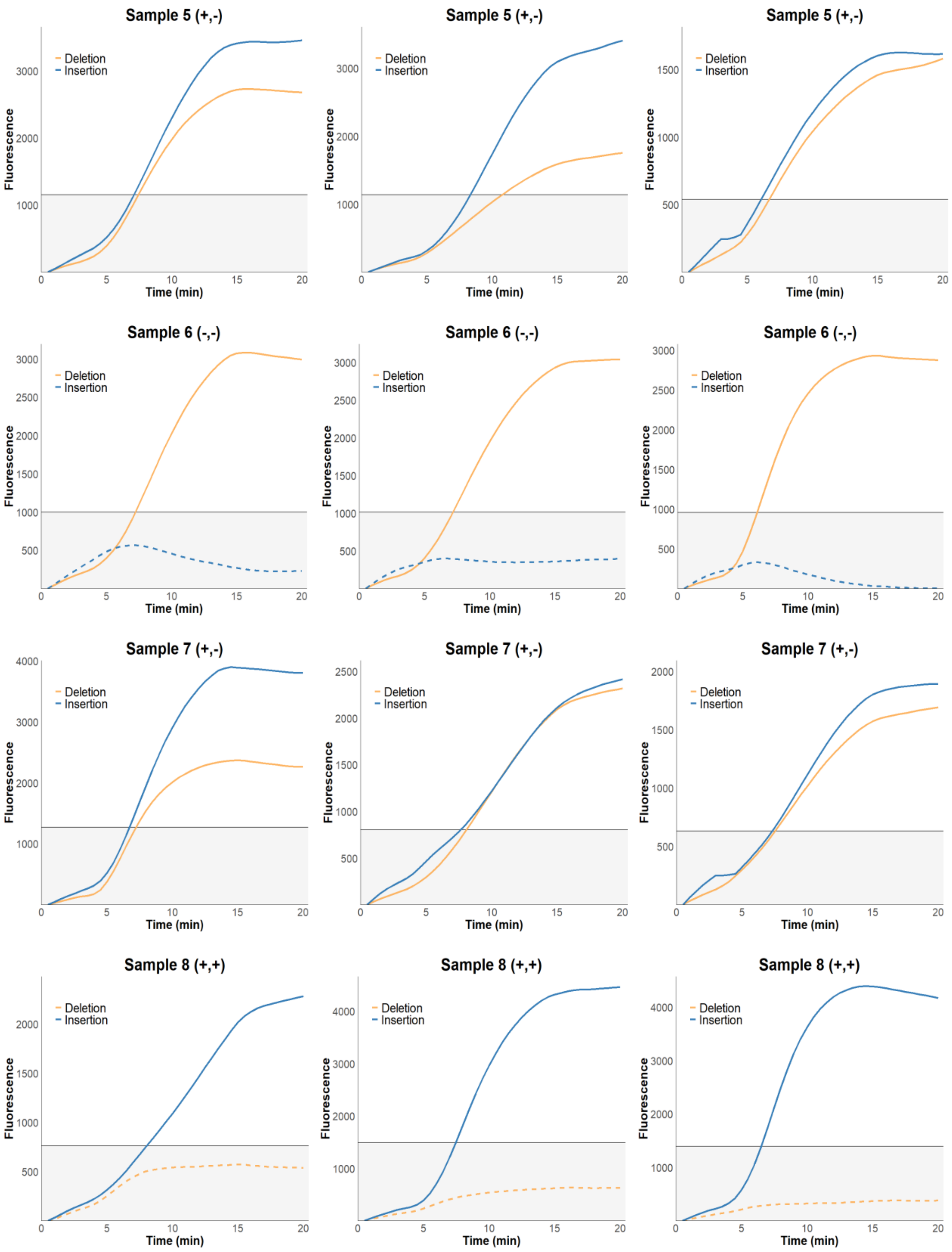
Sample 5 (+,-)
Sample 5 (+,-)
Sample 5 (+,-)
Sample 6 (-,-)
Sample 6 (-,-)
Sample 6 (-,-)
Sample 7 (+,-)
Sample 7 (+,-)
Sample 7 (+,-)
Sample 8 (+,+)
Sample 8 (+,+)
Sample 8 (+,+)
Deletion
Insertion
Fluorescence
Time (min)

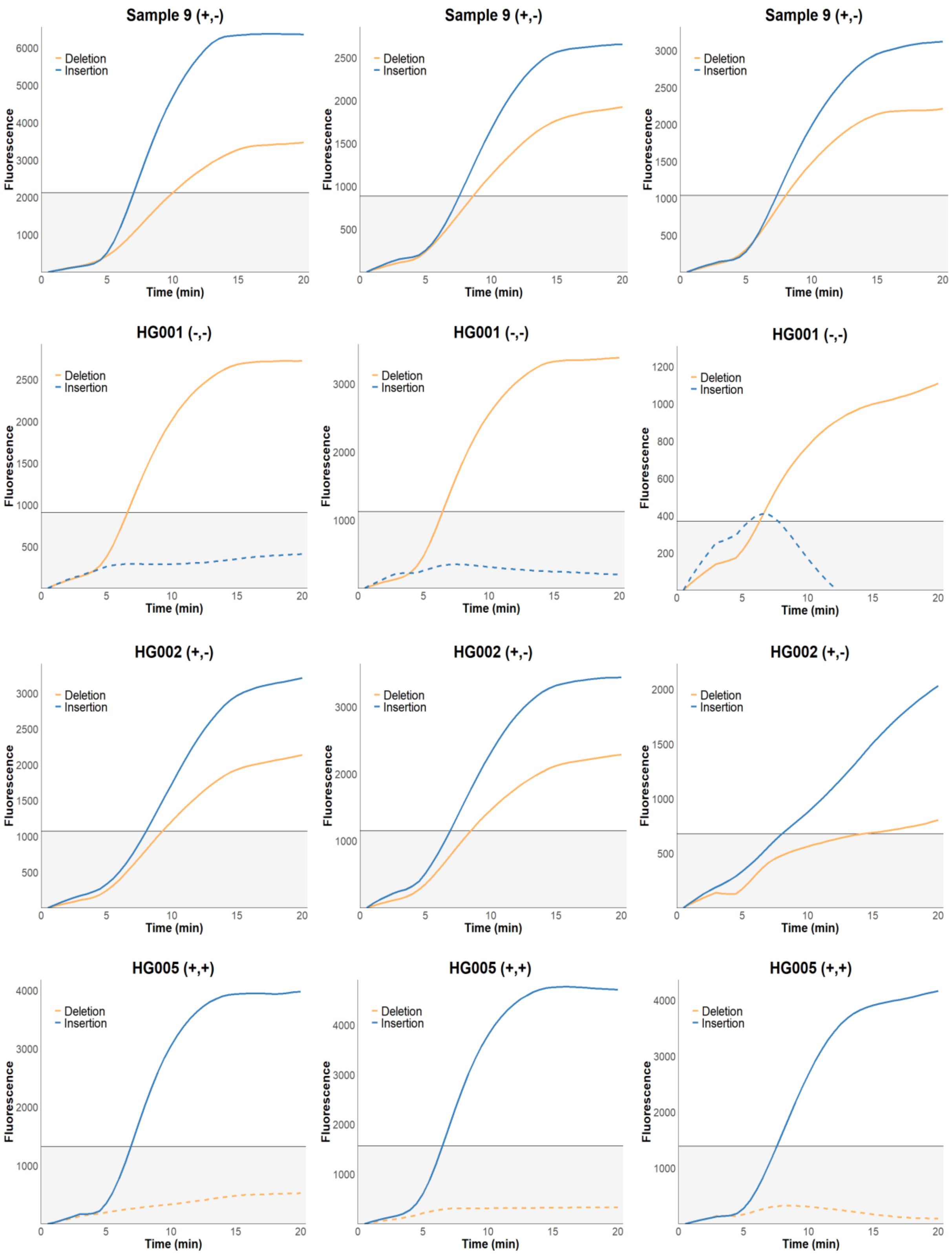

Sample 9 (+,-)
Deletion
Insertion
Fluorescence
Time (min)
Sample 9 (+,-)
Deletion
Insertion
Fluorescence
Time (min)
Sample 9 (+,-)
Deletion
Insertion
Fluorescence
Time (min)
HG001 (-,-)
Deletion
Insertion
Fluorescence
Time (min)
HG001 (-,-)
Deletion
Insertion
Fluorescence
Time (min)
HG001 (-,-)
Deletion
Insertion
Fluorescence
Time (min)
HG002 (+,-)
Deletion
Insertion
Fluorescence
Time (min)
HG002 (+,-)
Deletion
Insertion
Fluorescence
Time (min)
HG002 (+,-)
Deletion
Insertion
Fluorescence
Time (min)
HG005 (+,+)
Deletion
Insertion
Fluorescence
Time (min)
HG005 (+,+)
Deletion
Insertion
Fluorescence
Time (min)
HG005 (+,+)
Deletion
Insertion
Fluorescence
Time (min)

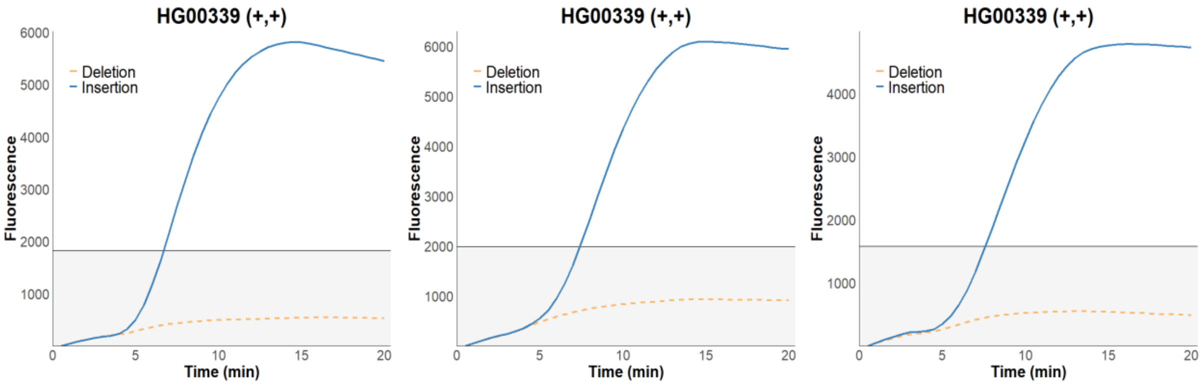
HG00339 (+,+)
Deletion
Insertion
Fluorescence
Time (min)
HG00339 (+,+)
Deletion
Insertion
Fluorescence
Time (min)
HG00339 (+,+)
Deletion
Insertion
Fluorescence
Time (min)

<u>Figure S8:</u> Real-time RPA graphs of locus ID04 obtained for all 12 samples in triplicate. Sample number and true genotype are indicated above each graph. Curves of alleles called as present are shown as full lines, while curves of not called alleles are shown as dashed lines. The horizontal grey line represents the genotyping threshold to call heterozygous samples at 1/3 of the endpoint value of the major curve.

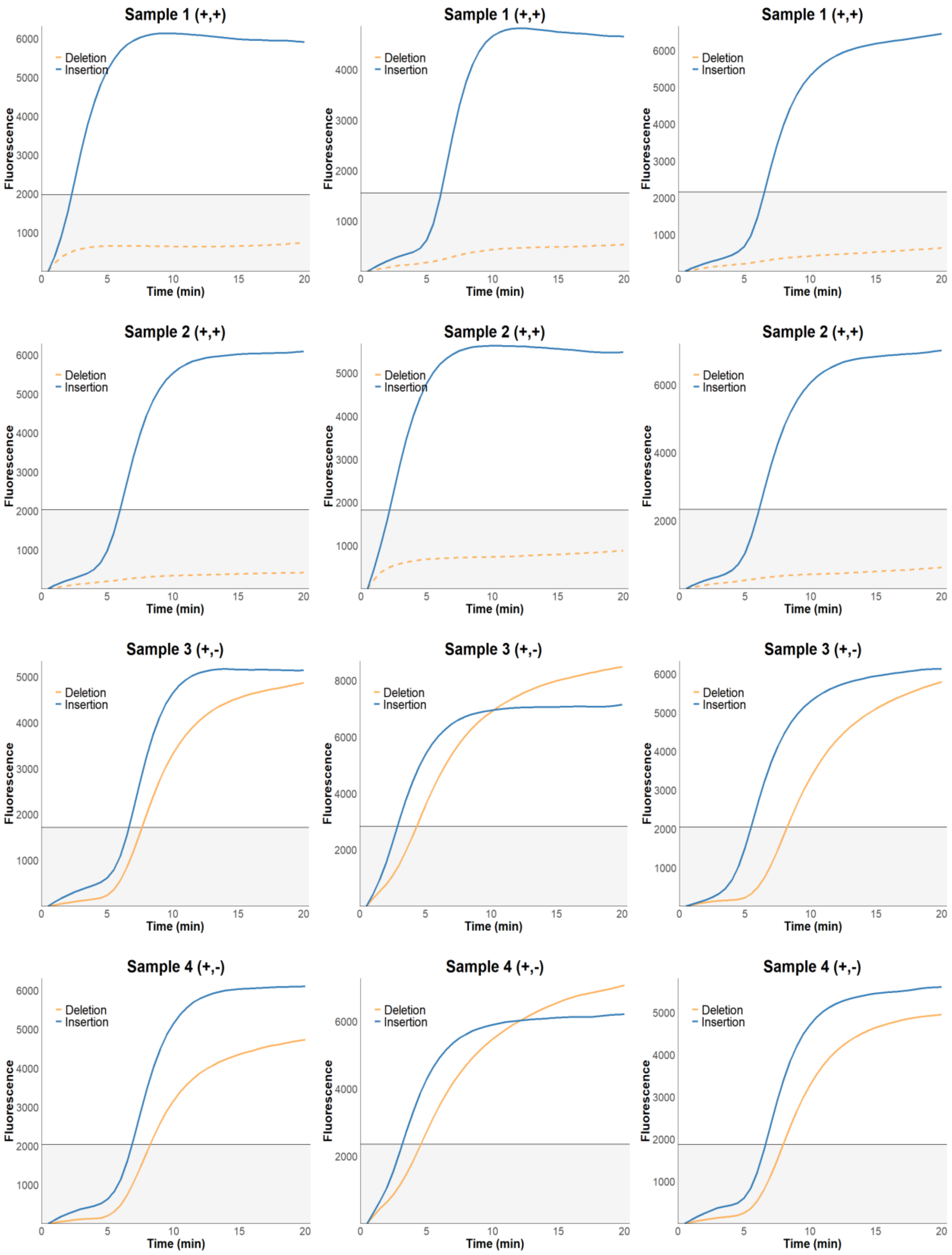

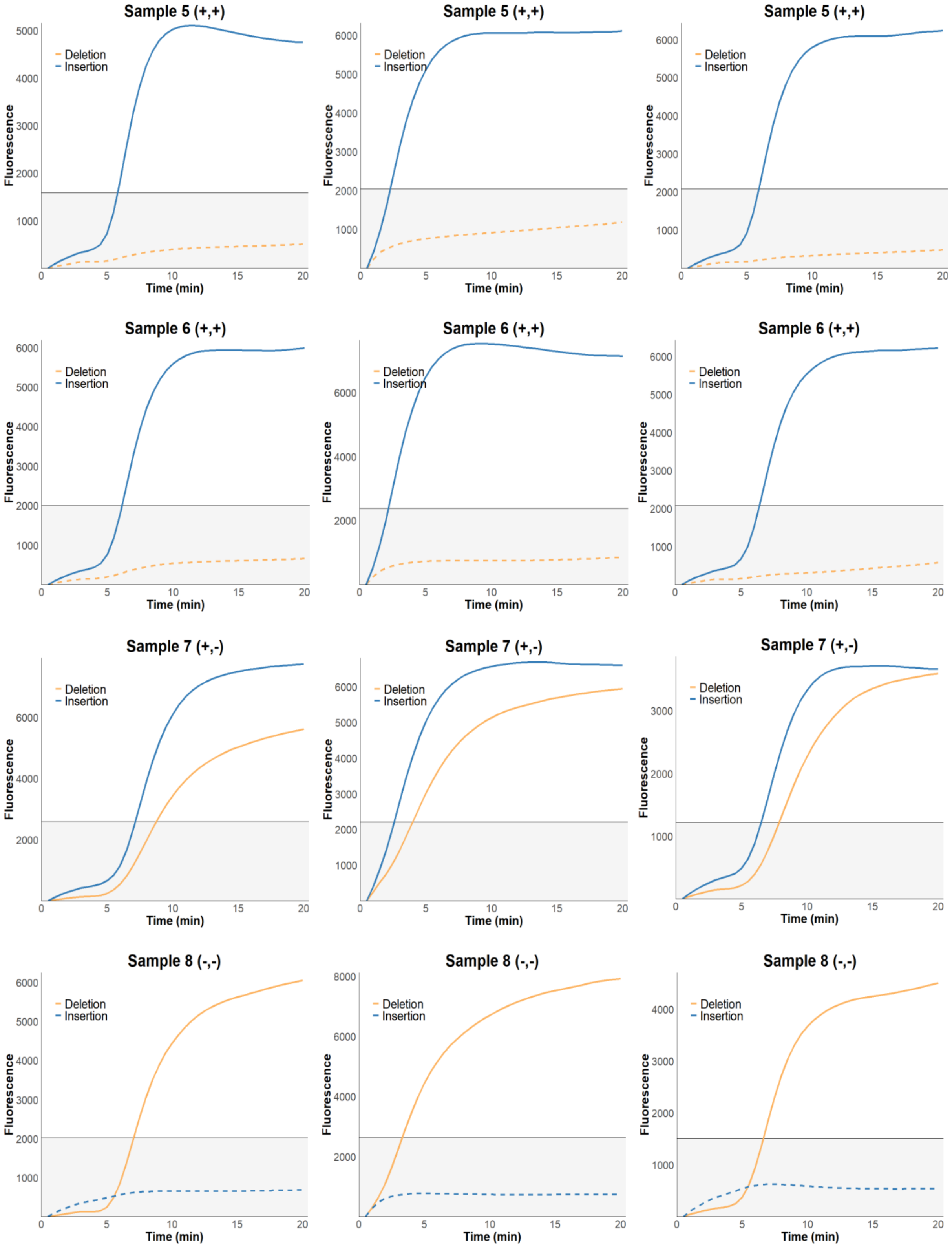
Sample 5 (+,+)
Sample 5 (+,+)
Sample 5 (+,+)
Sample 6 (+,+)
Sample 6 (+,+)
Sample 6 (+,+)
Sample 7 (+,-)
Sample 7 (+,-)
Sample 7 (+,-)
Sample 8 (-,-)
Sample 8 (-,-)
Sample 8 (-,-)
Deletion
Insertion
Fluorescence
Time (min)

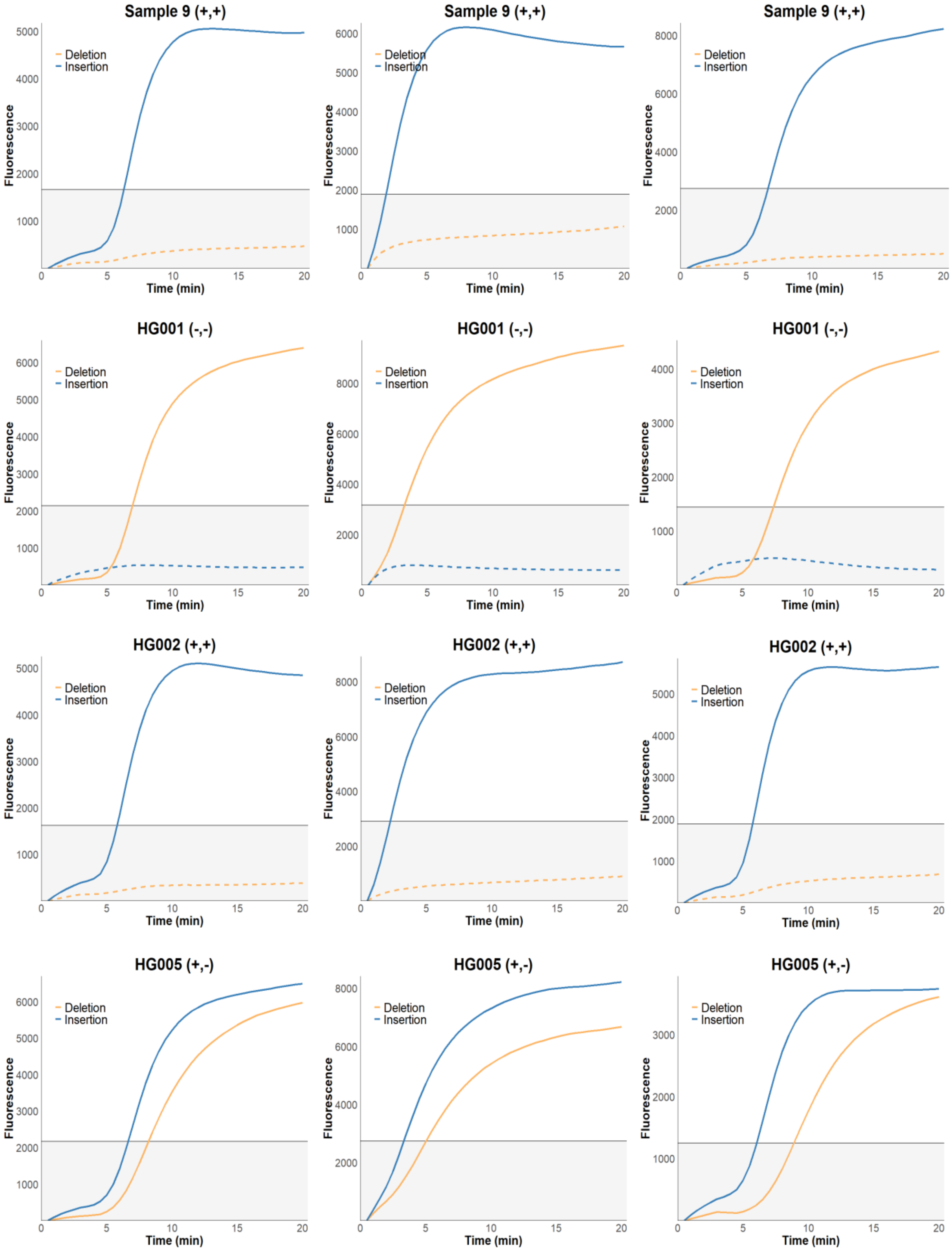
Sample 9 (+,+)
Deletion
Insertion
Fluorescence
Time (min)
Sample 9 (+,+)
Deletion
Insertion
Fluorescence
Time (min)
Sample 9 (+,+)
Deletion
Insertion
Fluorescence
Time (min)
HG001 (-,-)
Deletion
Insertion
Fluorescence
Time (min)
HG001 (-,-)
Deletion
Insertion
Fluorescence
Time (min)
HG001 (-,-)
Deletion
Insertion
Fluorescence
Time (min)
HG002 (+,+)
Deletion
Insertion
Fluorescence
Time (min)
HG002 (+,+)
Deletion
Insertion
Fluorescence
Time (min)
HG002 (+,+)
Deletion
Insertion
Fluorescence
Time (min)
HG005 (+,-)
Deletion
Insertion
Fluorescence
Time (min)
HG005 (+,-)
Deletion
Insertion
Fluorescence
Time (min)
HG005 (+,-)
Deletion
Insertion
Fluorescence
Time (min)

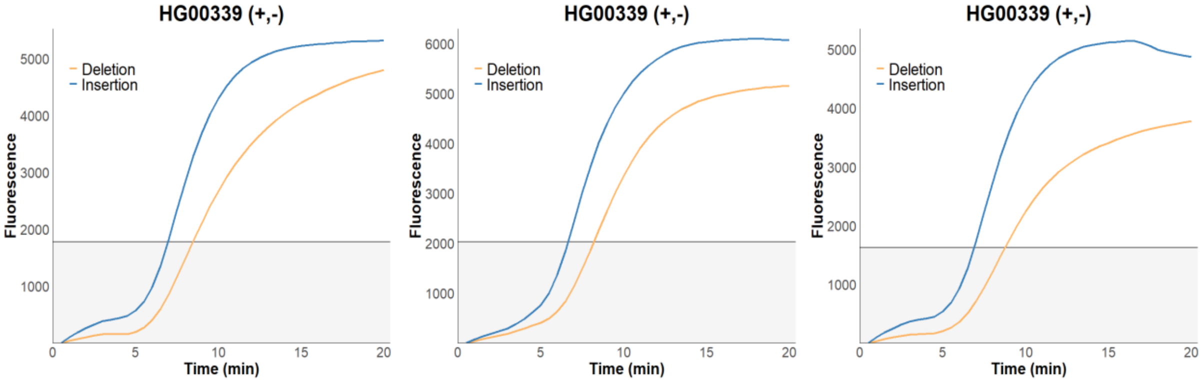
HG00339 (+,-)
Deletion
Insertion
Fluorescence
Time (min)
HG00339 (+,-)
Deletion
Insertion
Fluorescence
Time (min)
HG00339 (+,-)
Deletion
Insertion
Fluorescence
Time (min)

Figure S9: Real-time RPA graphs of locus ID05 obtained for all 12 samples in triplicate. Sample number and true genotype are indicated above each graph. Curves of alleles called as present are shown as full lines, while curves of not called alleles are shown as dashed lines. The horizontal grey line represents the genotyping threshold to call heterozygous samples at 1/3 of the endpoint value of the major curve.

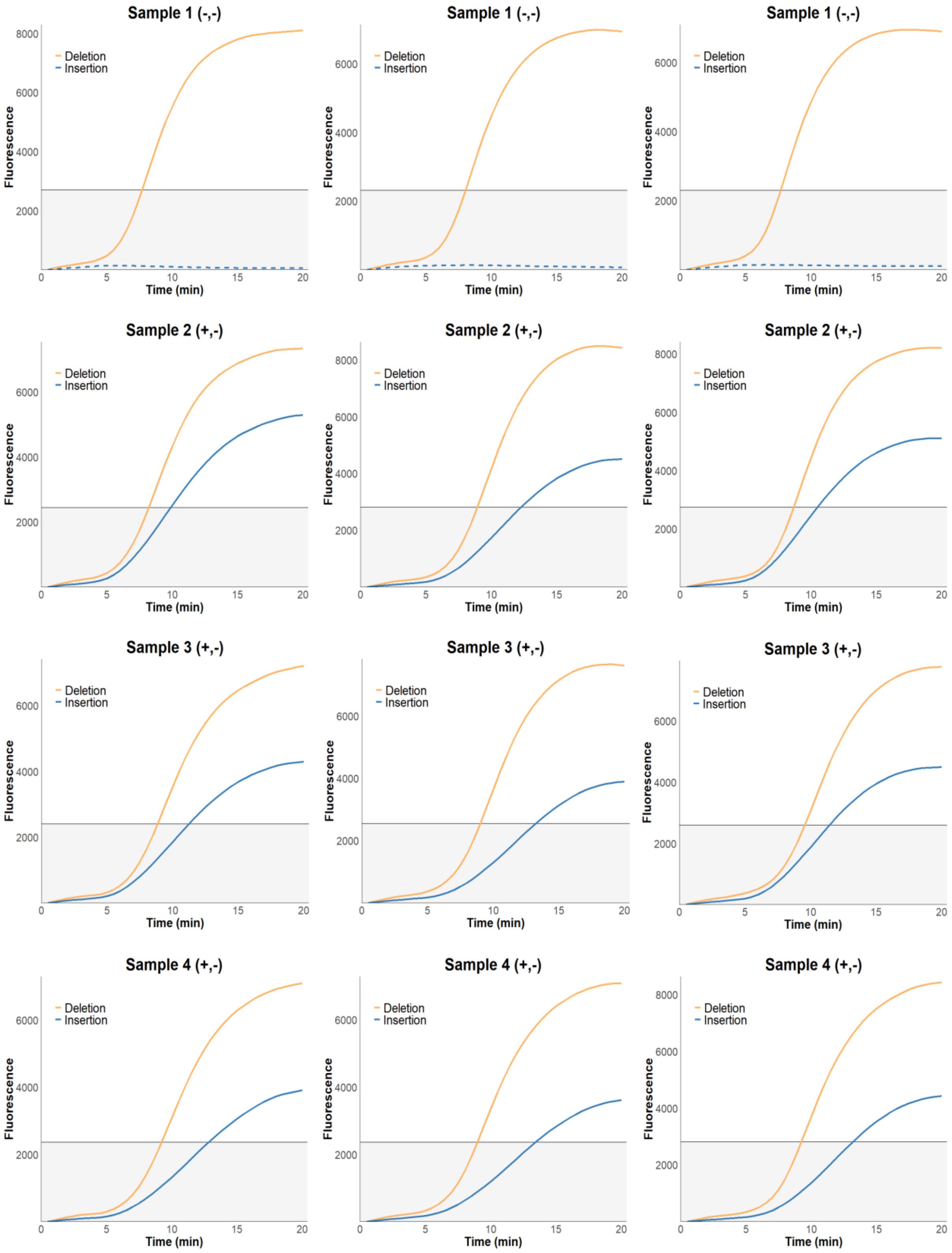

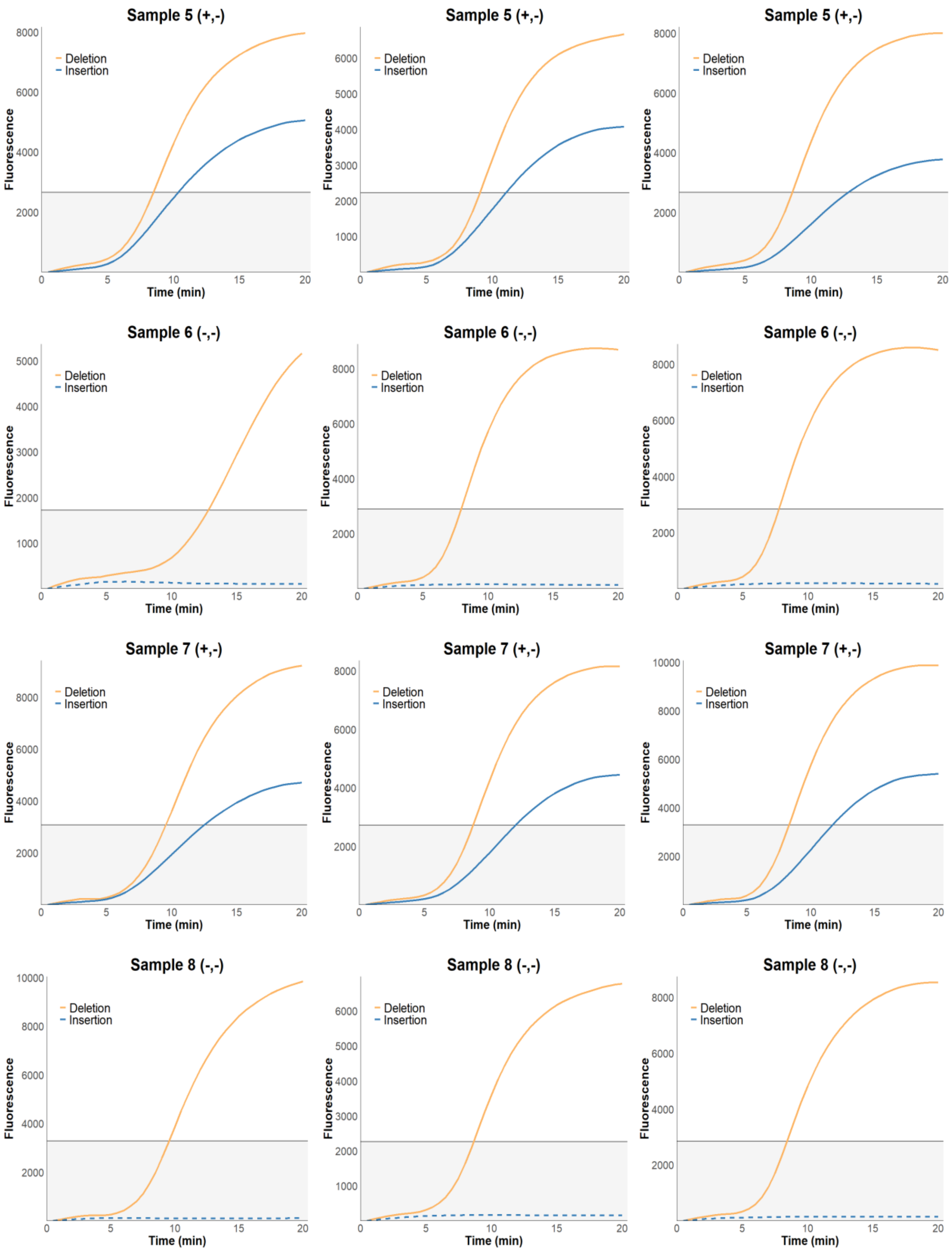
Sample 5 (+,-)
Deletion
Insertion
Fluorescence
Time (min)
Sample 5 (+,-)
Deletion
Insertion
Fluorescence
Time (min)
Sample 5 (+,-)
Deletion
Insertion
Fluorescence
Time (min)
Sample 6 (-,-)
Deletion
Insertion
Fluorescence
Time (min)
Sample 6 (-,-)
Deletion
Insertion
Fluorescence
Time (min)
Sample 6 (-,-)
Deletion
Insertion
Fluorescence
Time (min)
Sample 7 (+,-)
Deletion
Insertion
Fluorescence
Time (min)
Sample 7 (+,-)
Deletion
Insertion
Fluorescence
Time (min)
Sample 7 (+,-)
Deletion
Insertion
Fluorescence
Time (min)
Sample 8 (-,-)
Deletion
Insertion
Fluorescence
Time (min)
Sample 8 (-,-)
Deletion
Insertion
Fluorescence
Time (min)
Sample 8 (-,-)
Deletion
Insertion
Fluorescence
Time (min)

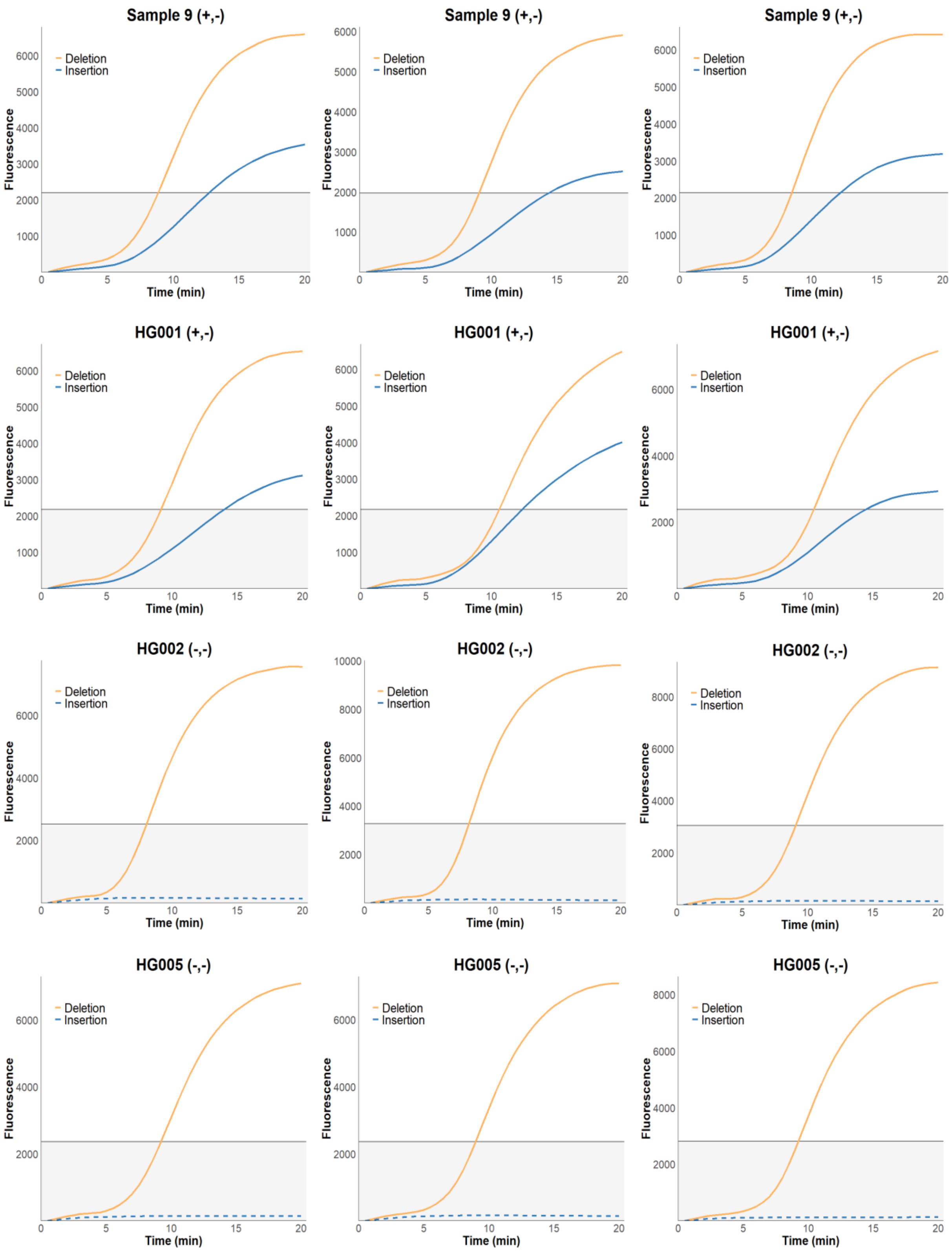
Sample 9 (+,-)
Sample 9 (+,-)
Sample 9 (+,-)
HG001 (+,-)
HG001 (+,-)
HG001 (+,-)
HG002 (-,-)
HG002 (-,-)
HG002 (-,-)
HG005 (-,-)
HG005 (-,-)
HG005 (-,-)
Deletion
Insertion
Fluorescence
Time (min)
0
5
10
15
20
1000
2000
3000
4000
5000
6000
8000
10000

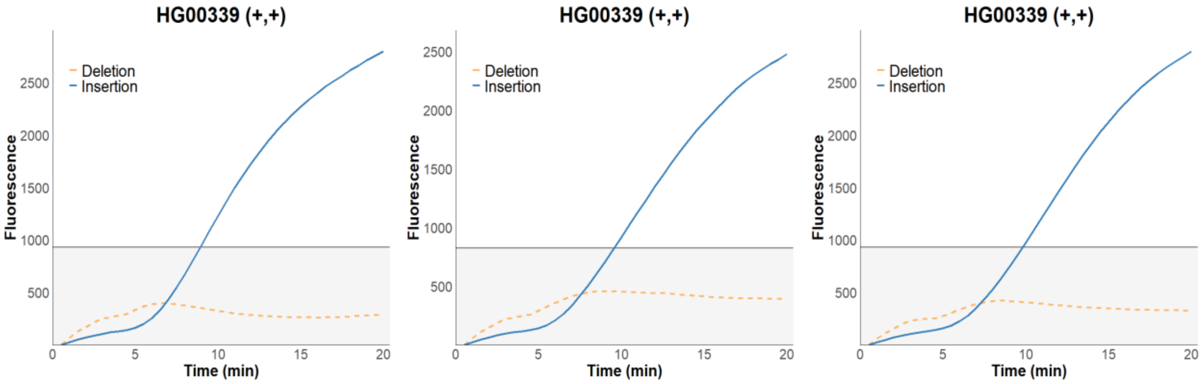

HG00339 (+,+)
HG00339 (+,+)
HG00339 (+,+)
Deletion
Insertion
Fluorescence
Time (min)

Figure S10: Real-time RPA graphs of locus ID06 obtained for all 12 samples in triplicate. Sample number and true genotype are indicated above each graph. Curves of alleles called as present are shown as full lines, while curves of not called alleles are shown as dashed lines. The horizontal grey line represents the genotyping threshold to call heterozygous samples at 1/3 of the endpoint value of the major curve.

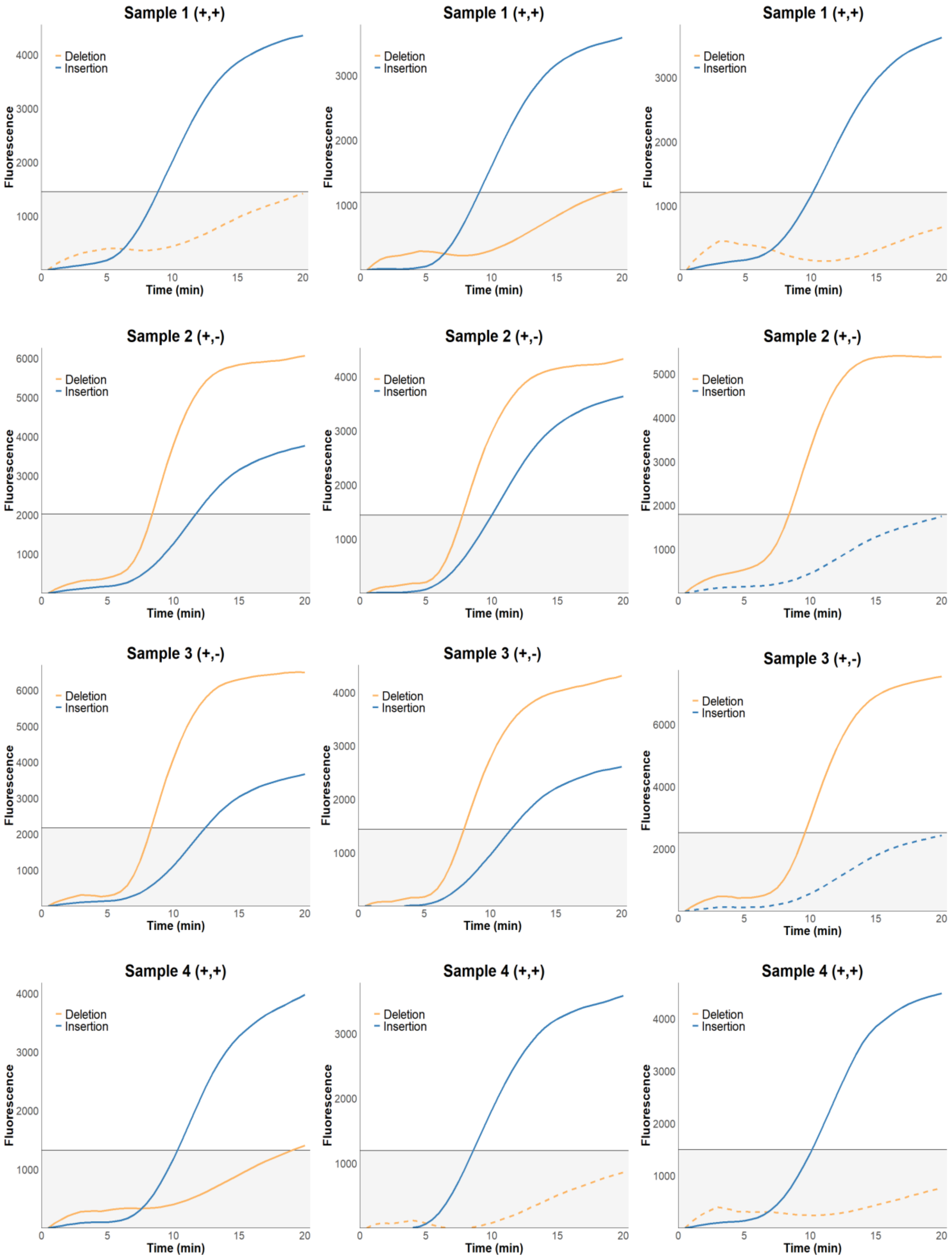

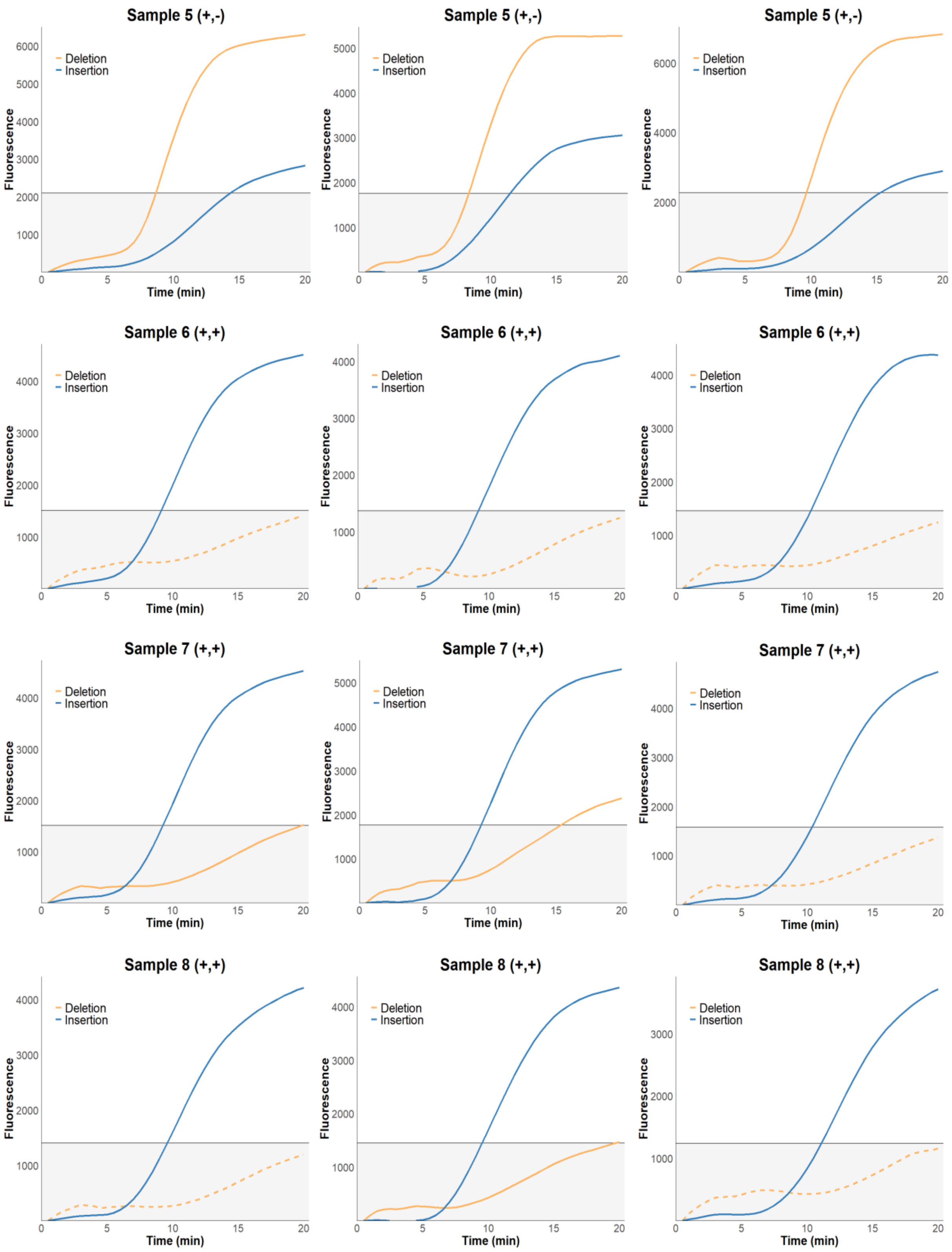
Sample 5 (+,-)
Deletion
Insertion
Fluorescence
Time (min)
Sample 5 (+,-)
Deletion
Insertion
Fluorescence
Time (min)
Sample 5 (+,-)
Deletion
Insertion
Fluorescence
Time (min)
Sample 6 (+,+)
Deletion
Insertion
Fluorescence
Time (min)
Sample 6 (+,+)
Deletion
Insertion
Fluorescence
Time (min)
Sample 6 (+,+)
Deletion
Insertion
Fluorescence
Time (min)
Sample 7 (+,+)
Deletion
Insertion
Fluorescence
Time (min)
Sample 7 (+,+)
Deletion
Insertion
Fluorescence
Time (min)
Sample 7 (+,+)
Deletion
Insertion
Fluorescence
Time (min)
Sample 8 (+,+)
Deletion
Insertion
Fluorescence
Time (min)
Sample 8 (+,+)
Deletion
Insertion
Fluorescence
Time (min)
Sample 8 (+,+)
Deletion
Insertion
Fluorescence
Time (min)

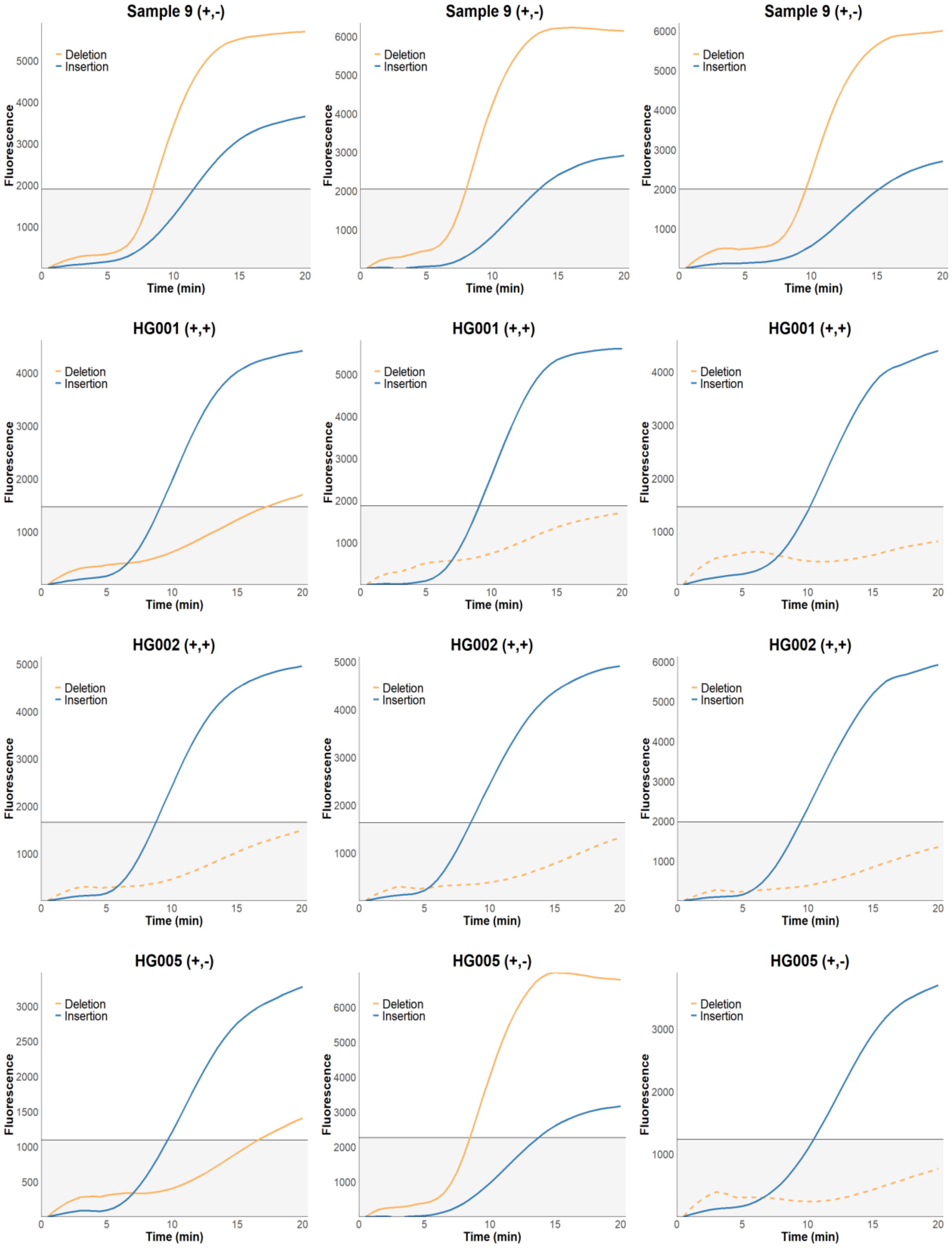
Sample 9 (+,-)
Sample 9 (+,-)
Sample 9 (+,-)
HG001 (+,+)
HG001 (+,+)
HG001 (+,+)
HG002 (+,+)
HG002 (+,+)
HG002 (+,+)
HG005 (+,-)
HG005 (+,-)
HG005 (+,-)
Deletion
Insertion
Fluorescence
Time (min)

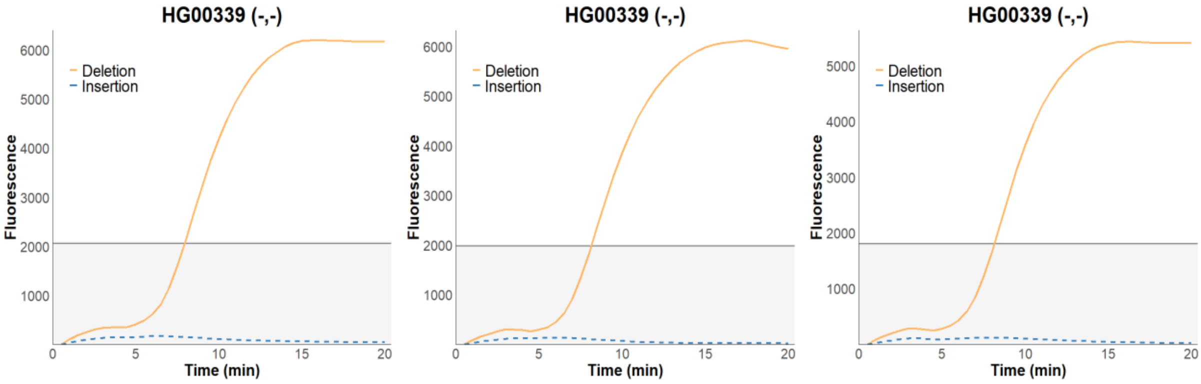
HG00339 (-,-)
Deletion
Insertion
Fluorescence
Time (min)
HG00339 (-,-)
Deletion
Insertion
Fluorescence
Time (min)
HG00339 (-,-)
Deletion
Insertion
Fluorescence
Time (min)

Figure S11: Real-time RPA graphs of locus ID07 obtained for all 12 samples in triplicate. Sample number and true genotype are indicated above each graph. Curves of alleles called as present are shown as full lines, while curves of not called alleles are shown as dashed lines. The horizontal grey line represents the genotyping threshold to call heterozygous samples at 1/3 of the endpoint value of the major curve.

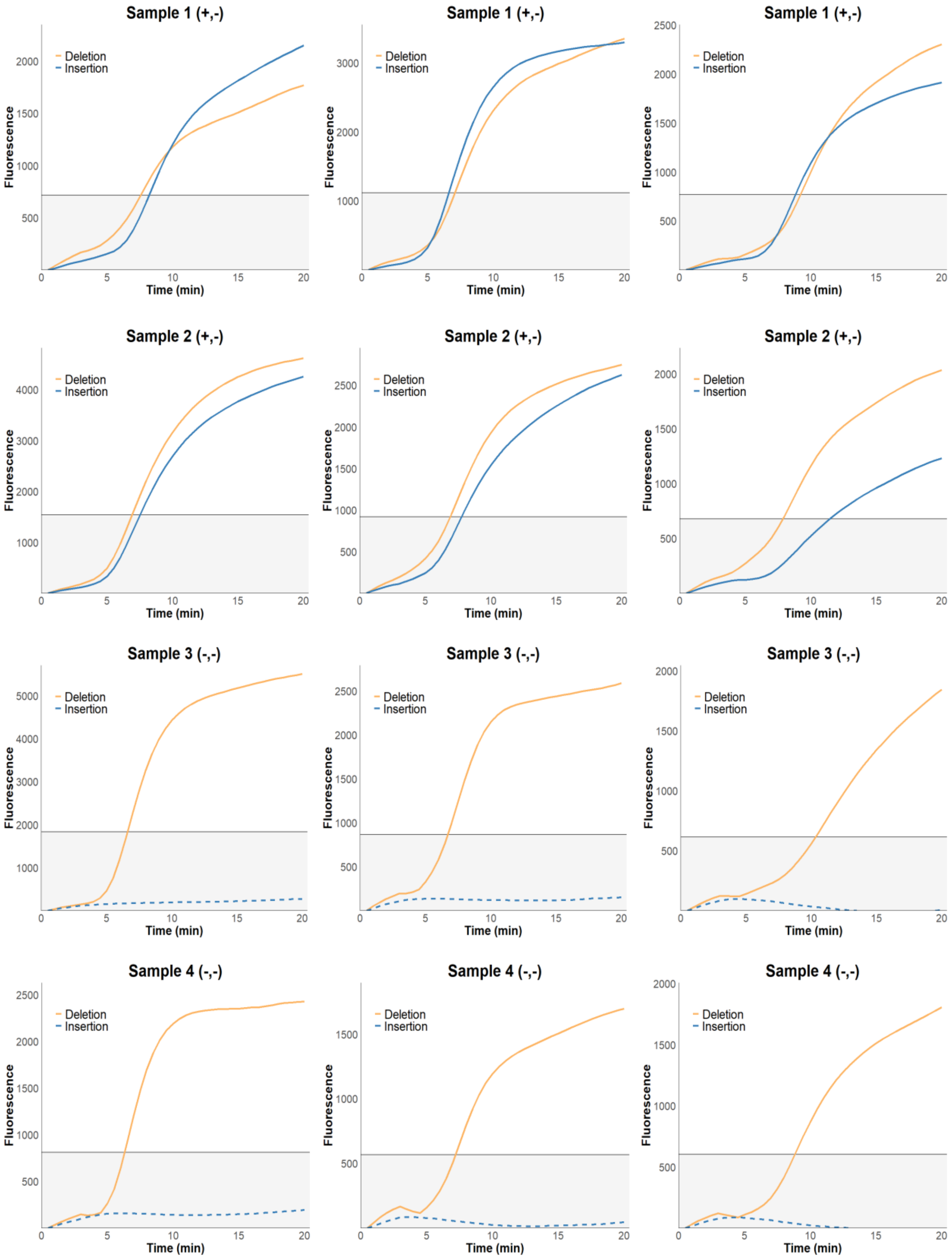

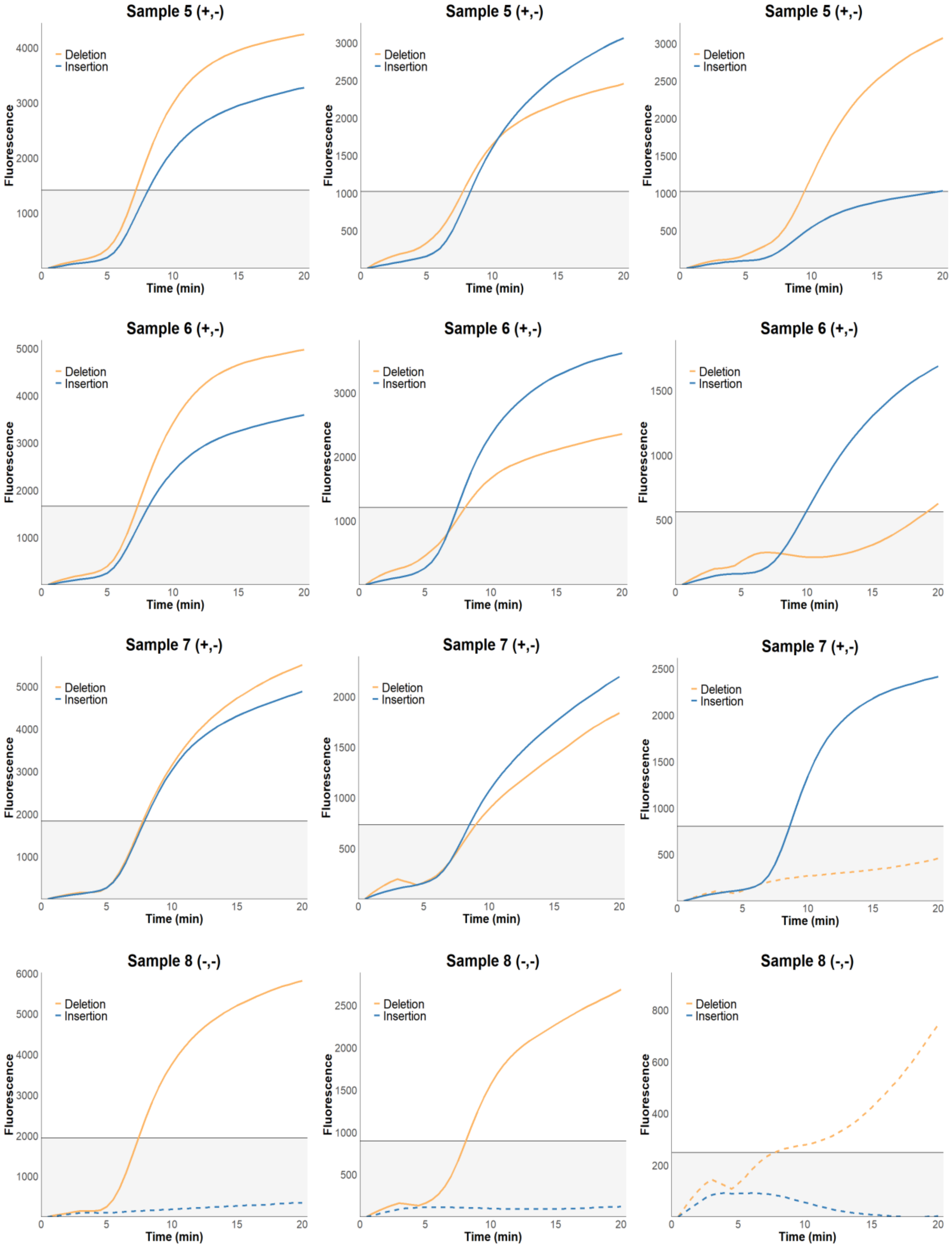
Sample 5 (+,-)
Sample 5 (+,-)
Sample 5 (+,-)
Sample 6 (+,-)
Sample 6 (+,-)
Sample 6 (+,-)
Sample 7 (+,-)
Sample 7 (+,-)
Sample 7 (+,-)
Sample 8 (-,-)
Sample 8 (-,-)
Sample 8 (-,-)
Deletion
Insertion
Fluorescence
Time (min)

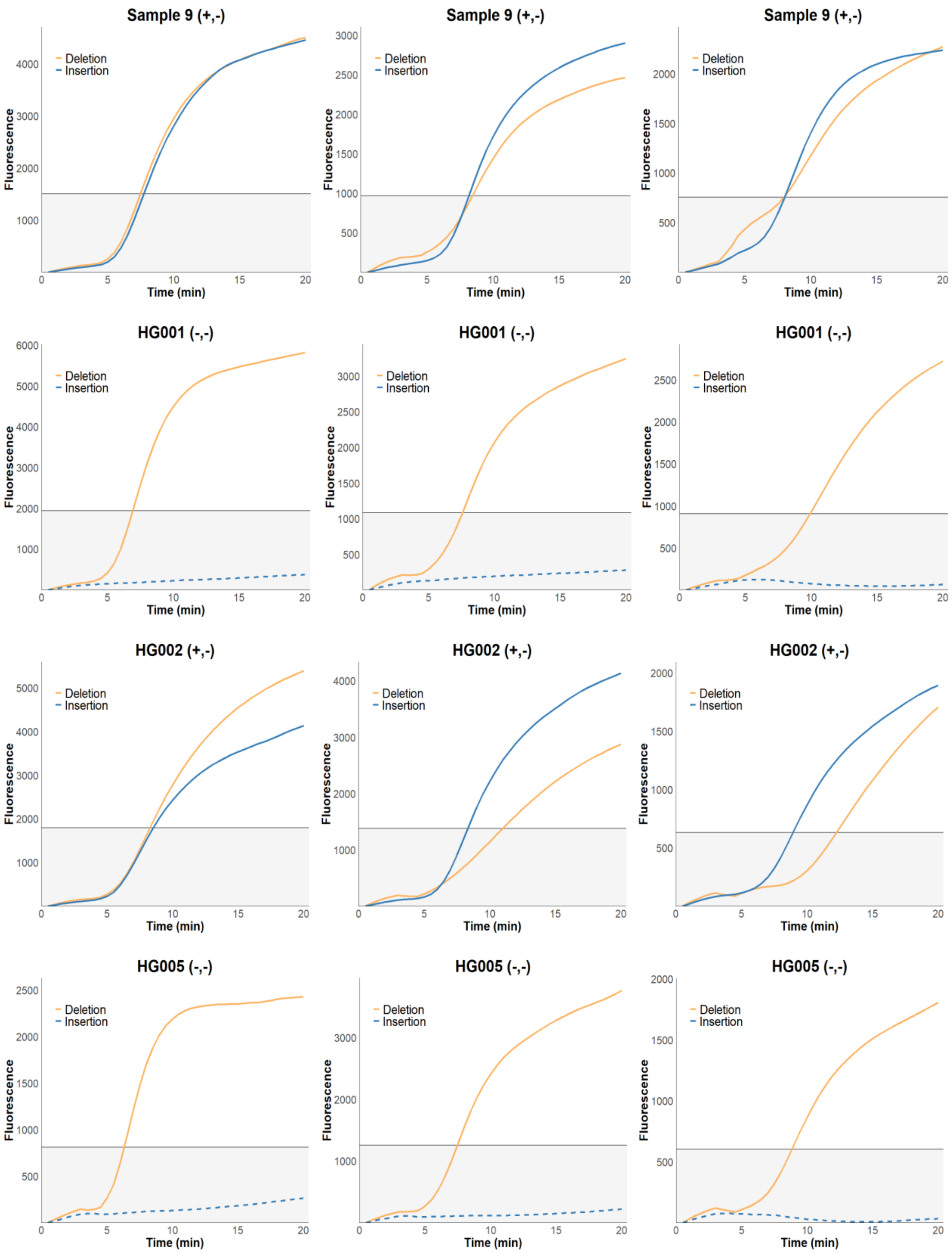
Sample 9 (+,-)
Deletion
Insertion
Fluorescence
Time (min)
Sample 9 (+,-)
Deletion
Insertion
Fluorescence
Time (min)
Sample 9 (+,-)
Deletion
Insertion
Fluorescence
Time (min)
HG001 (-,-)
Deletion
Insertion
Fluorescence
Time (min)
HG001 (-,-)
Deletion
Insertion
Fluorescence
Time (min)
HG001 (-,-)
Deletion
Insertion
Fluorescence
Time (min)
HG002 (+,-)
Deletion
Insertion
Fluorescence
Time (min)
HG002 (+,-)
Deletion
Insertion
Fluorescence
Time (min)
HG002 (+,-)
Deletion
Insertion
Fluorescence
Time (min)
HG005 (-,-)
Deletion
Insertion
Fluorescence
Time (min)
HG005 (-,-)
Deletion
Insertion
Fluorescence
Time (min)
HG005 (-,-)
Deletion
Insertion
Fluorescence
Time (min)

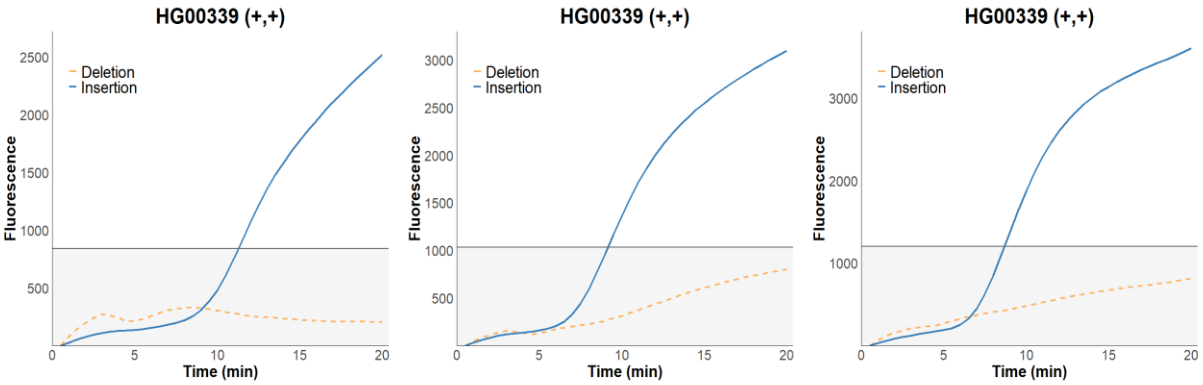
HG00339 (+,+)
Deletion
Insertion
Fluorescence
Time (min)
HG00339 (+,+)
Deletion
Insertion
Fluorescence
Time (min)
HG00339 (+,+)
Deletion
Insertion
Fluorescence
Time (min)

Figure S12: Primer and probe concentration optimization for locus ID02. Real-time RPA graphs obtained for sample HG001 and HG002 are given for 1 ng DNA input. Sample name, true genotype and input levels are indicated above each graph. Curves of alleles that are called as present are represented as full lines, while curves of not-called alleles are represented as dashed lines.

HG001 (+,+)
Primer concentration

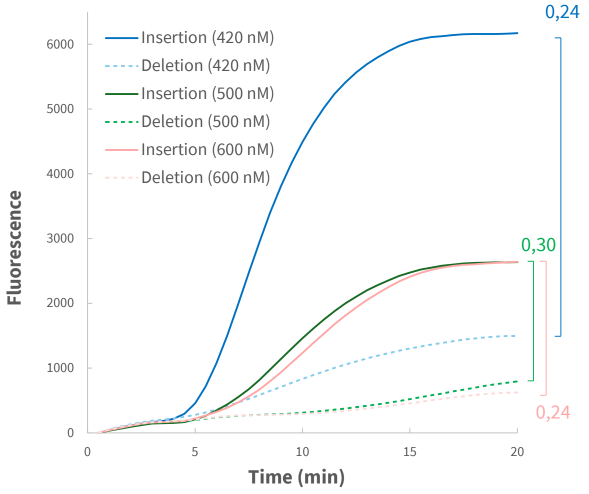

HG002 (+,-)
Primer concentration

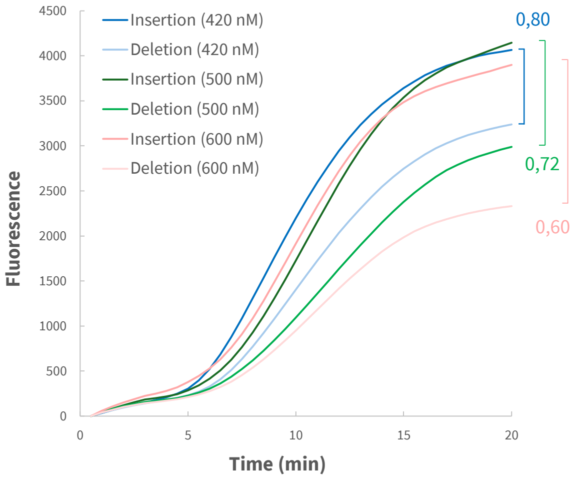

HG001 (+,+)
Probe concentration

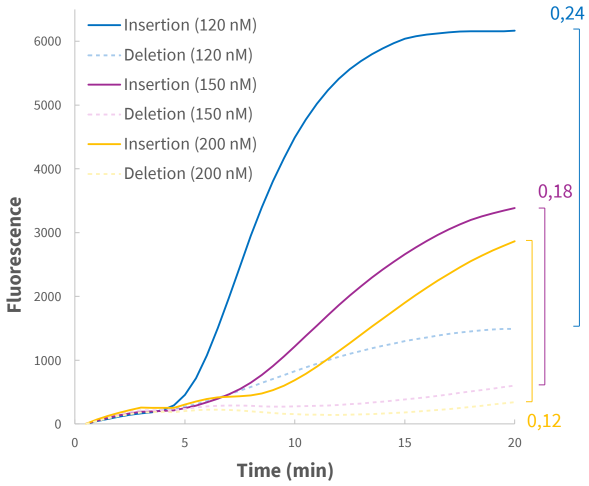

HG002 (+,-)
Probe concentration

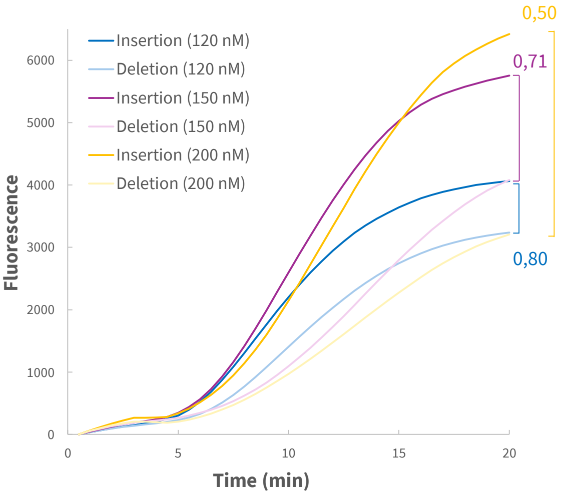

Figure S13: Temperature optimization for locus ID02. Real-time RPA graphs obtained for sample HG001 and HG002 are given for 1 ng DNA input. Sample name, true genotype and input levels are indicated above each graph. Curves of alleles that are called as present are represented as full lines, while curves of not-called alleles are represented as dashed lines. The horizontal grey line represents the heterozygosity threshold at 1/3 of the endpoint value of the major curve.

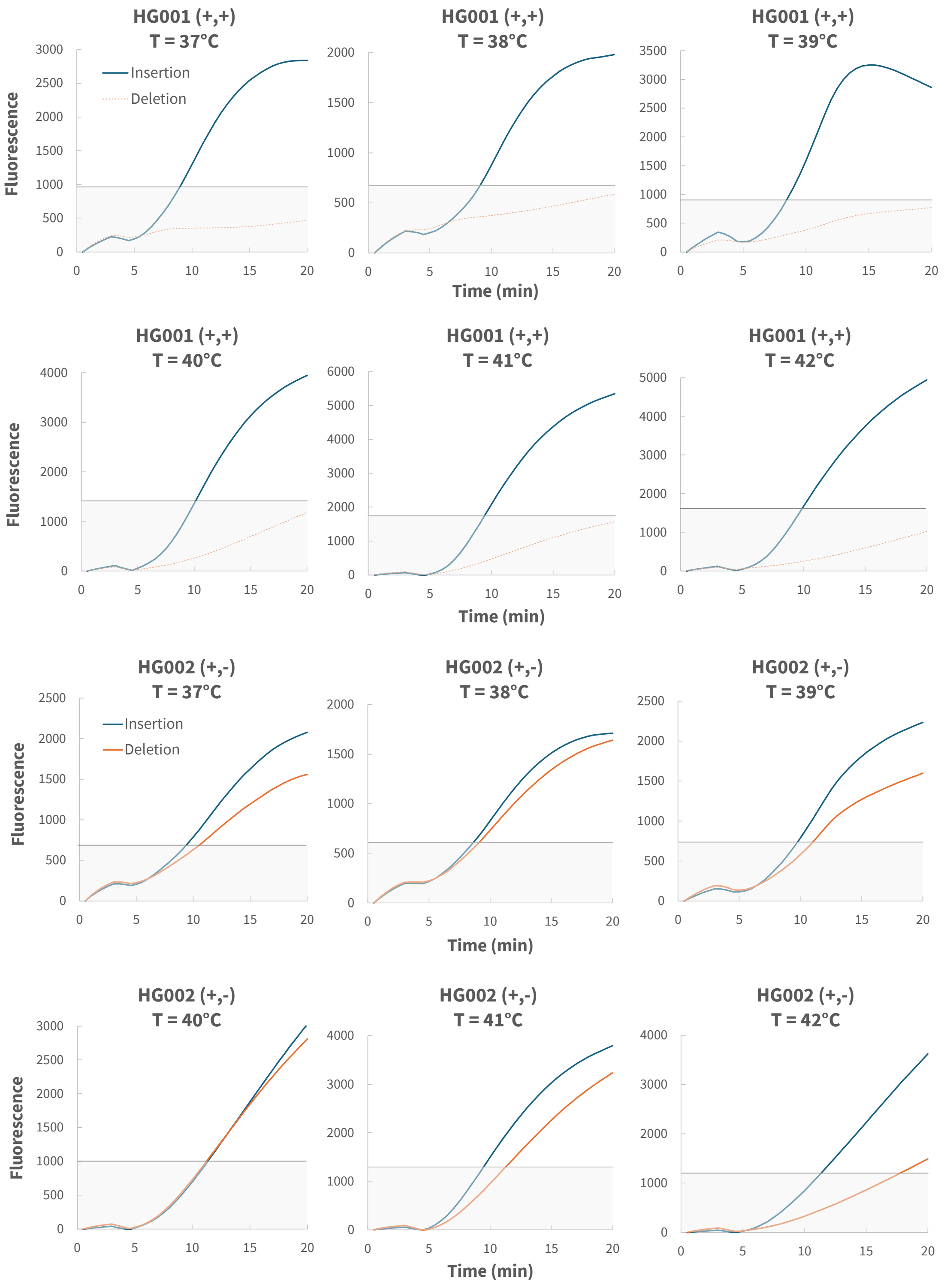

Figure S14: Sensitivity assessment for locus Amelogenin. Real-time RPA graphs obtained for sample 1,2 and 6 are given for decreasing DNA inputs (500 – 31 pg). Sample name, true genotype and input levels are indicated above each graph. Curves of alleles that are called as present are represented as full lines, while curves of not-called alleles are represented as dashed lines. The horizontal grey line displays the 1000 unit threshold.

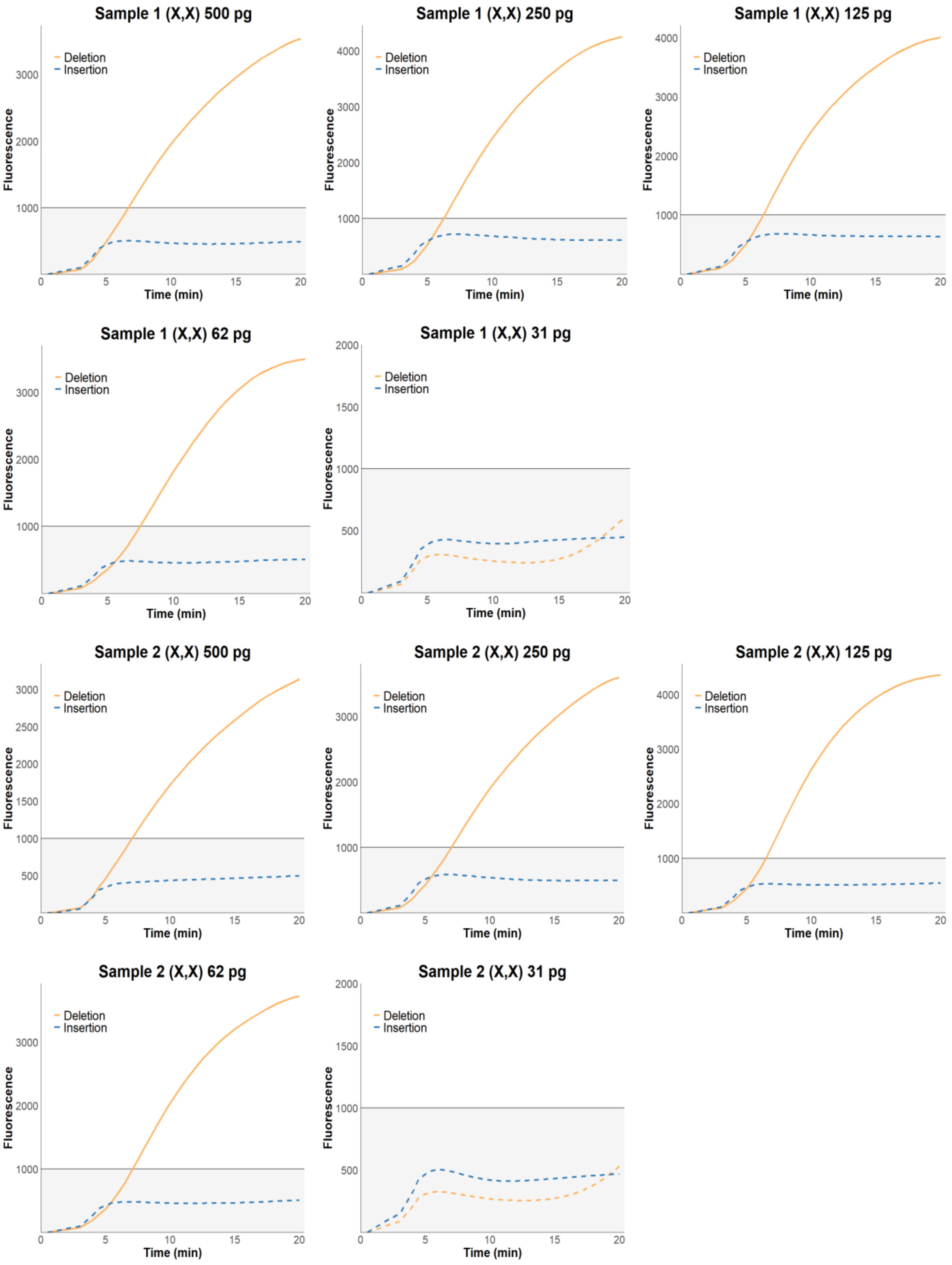

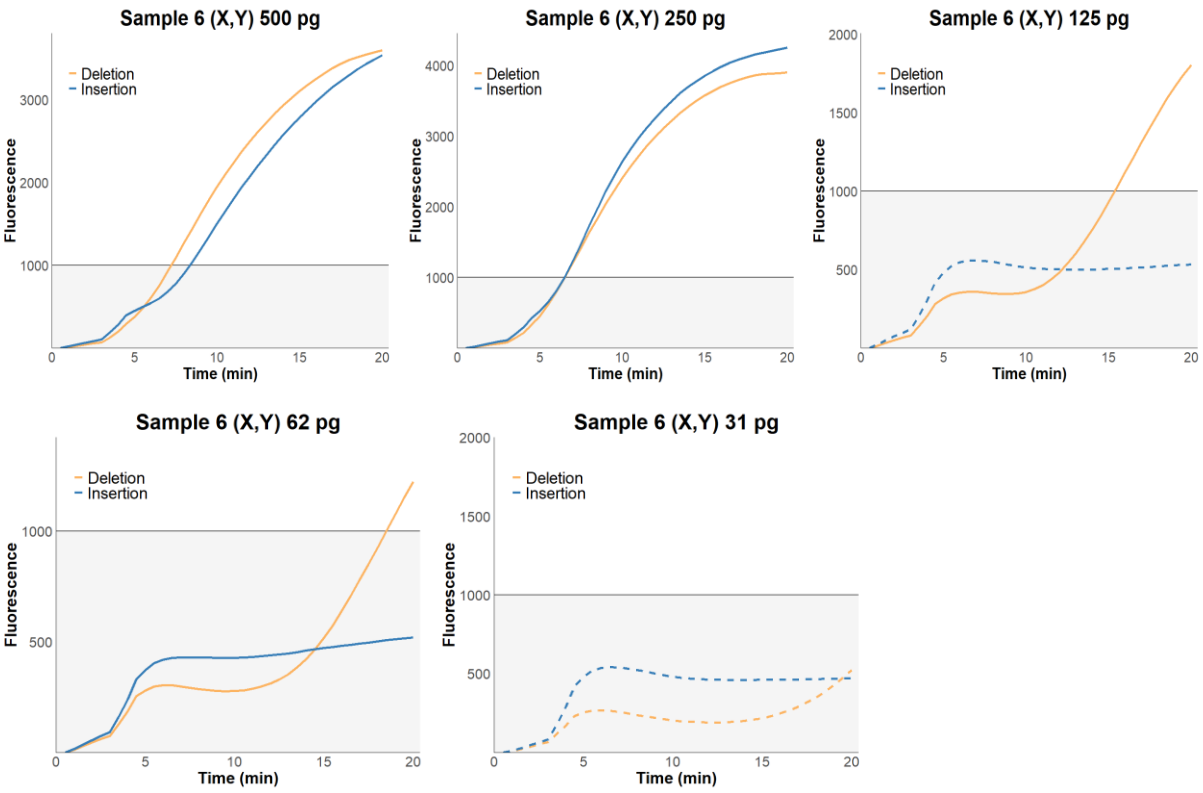
Sample 6 (X,Y) 500 pg
Sample 6 (X,Y) 250 pg
Sample 6 (X,Y) 125 pg
Sample 6 (X,Y) 62 pg
Sample 6 (X,Y) 31 pg
Deletion
Insertion
Fluorescence
Time (min)

Figure S15: Sensitivity assessment for locus ID01. Real-time RPA graphs obtained for sample 1,2 and 6 are given for decreasing DNA inputs (500 – 31 pg). Sample name, true genotype and input levels are indicated above each graph. Curves of alleles that are called as present are represented as full lines, while curves of not-called alleles are represented as dashed lines. The horizontal grey line displays the 1000 unit threshold.

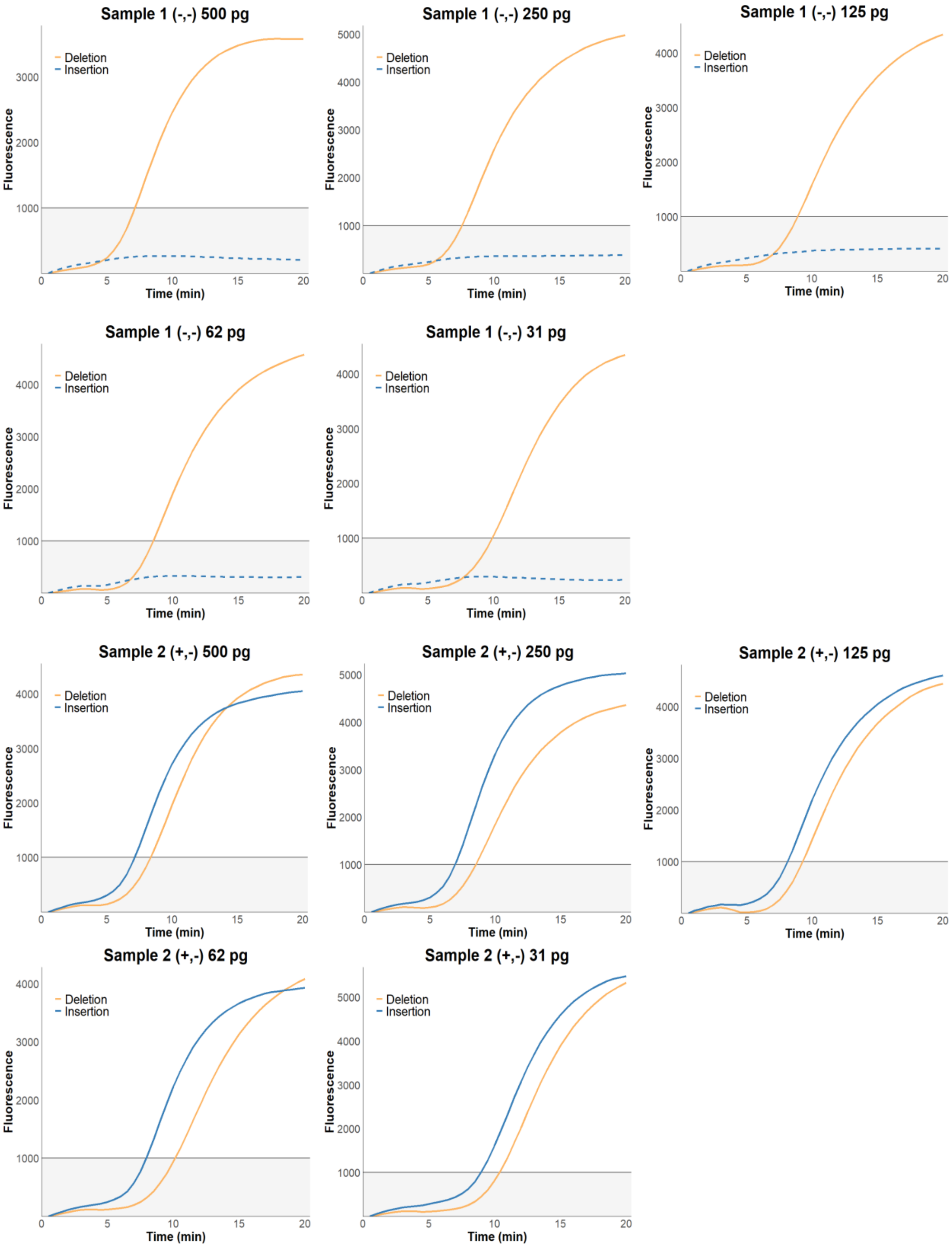

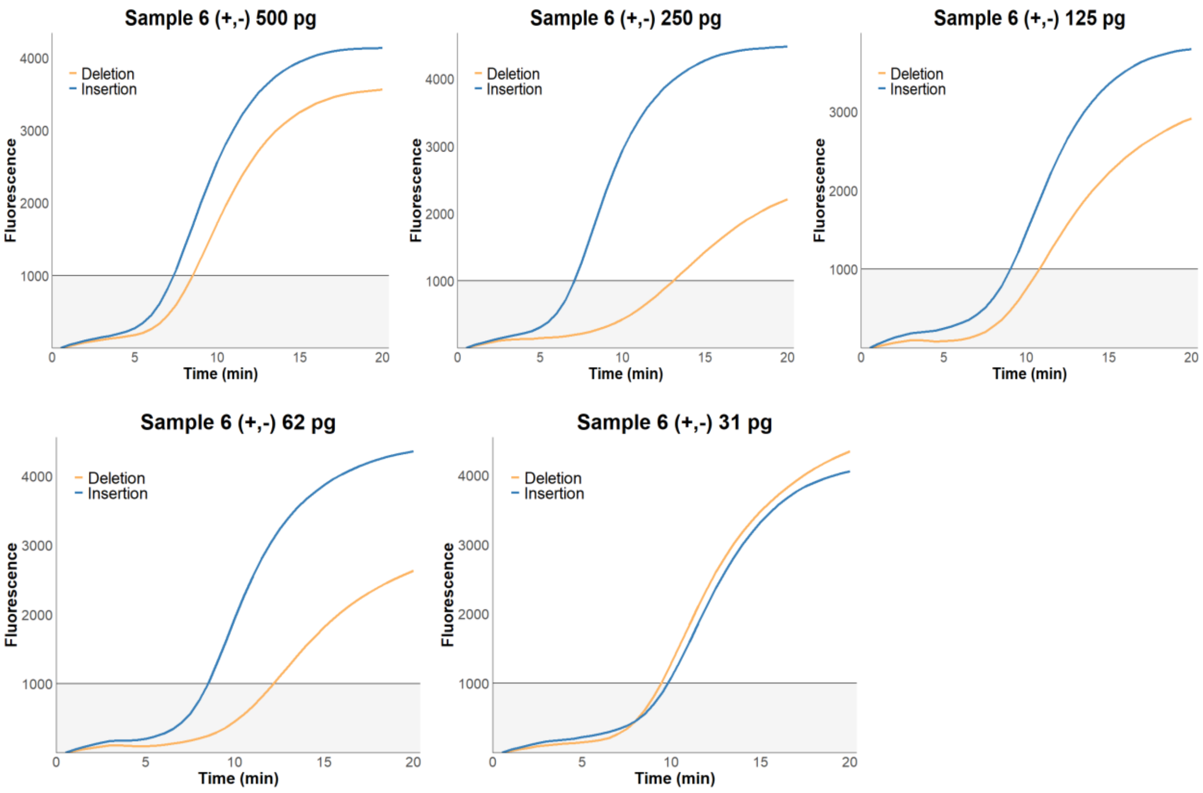
Sample 6 (+,-) 500 pg
Sample 6 (+,-) 250 pg
Sample 6 (+,-) 125 pg
Sample 6 (+,-) 62 pg
Sample 6 (+,-) 31 pg
Deletion
Insertion
Fluorescence
Time (min)

Figure S16: Sensitivity assessment for locus ID02. Real-time RPA graphs obtained for sample 1,2 and 6 are given for decreasing DNA inputs (500 – 31 pg). Sample name, true genotype and input levels are indicated above each graph. Curves of alleles that are called as present are represented as full lines, while curves of not-called alleles are represented as dashed lines. The horizontal grey line displays the 1000 unit threshold.

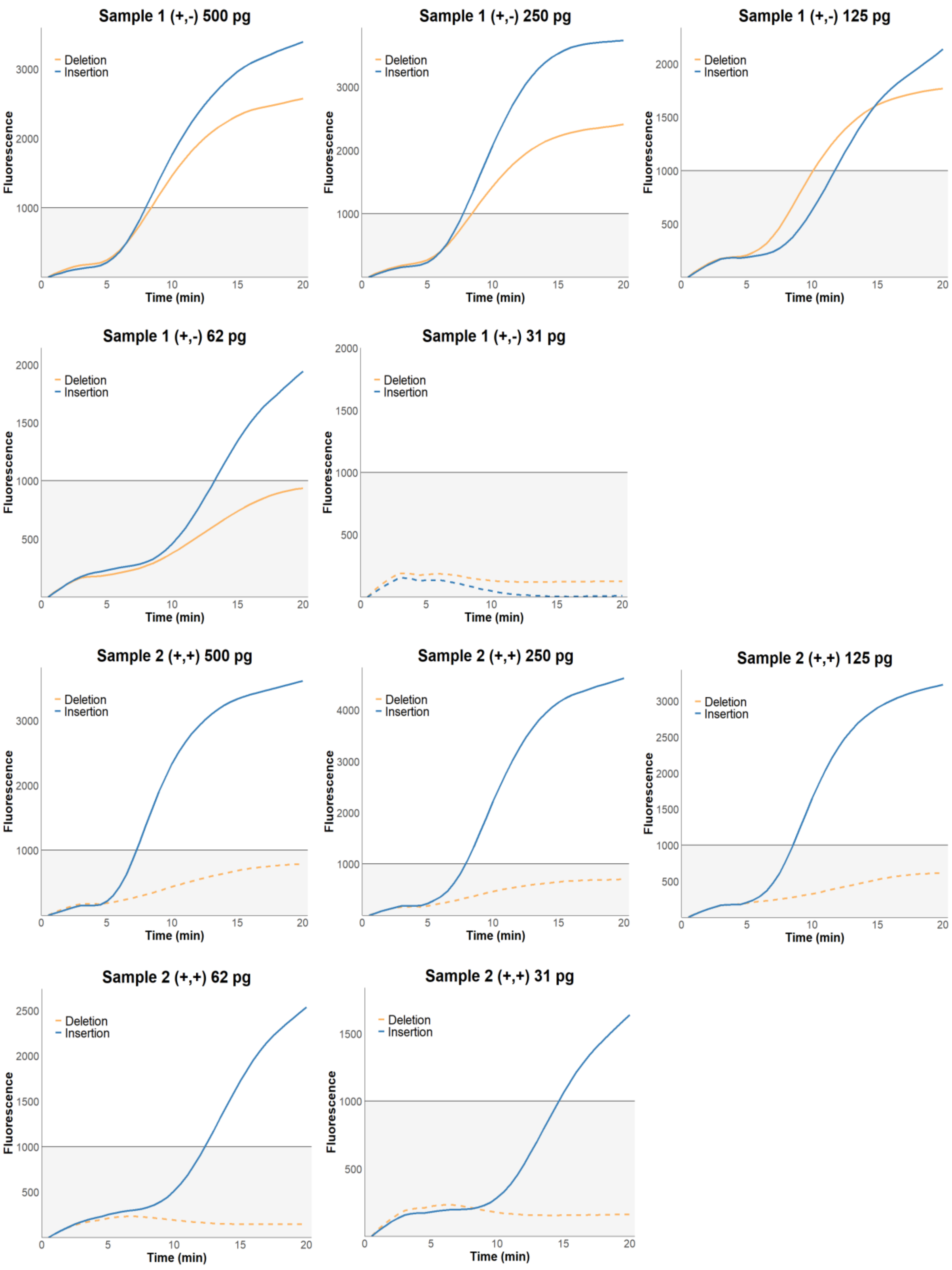

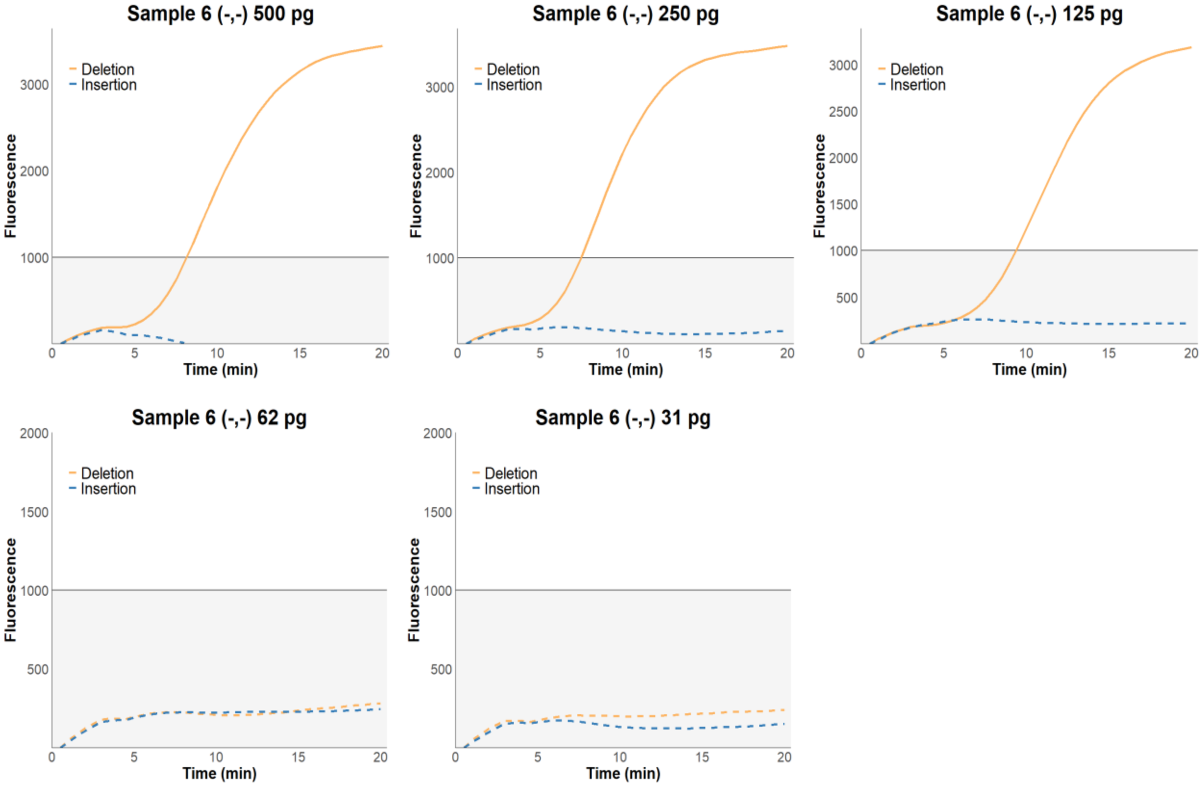
Sample 6 (-,-) 500 pg
Sample 6 (-,-) 250 pg
Sample 6 (-,-) 125 pg
Sample 6 (-,-) 62 pg
Sample 6 (-,-) 31 pg
Deletion
Insertion
Fluorescence
Time (min)
0
5
10
15
20
500
1000
1500
2000
2500
3000

<u>Figure S17:</u> Sensitivity assessment for locus ID03. Real-time RPA graphs obtained for sample 1,2 and 6 are given for decreasing DNA inputs (500 – 31 pg). Sample name, true genotype and input levels are indicated above each graph. Curves of alleles that are called as present are represented as full lines, while curves of not-called alleles are represented as dashed lines. The horizontal grey line displays the 1000 unit threshold.

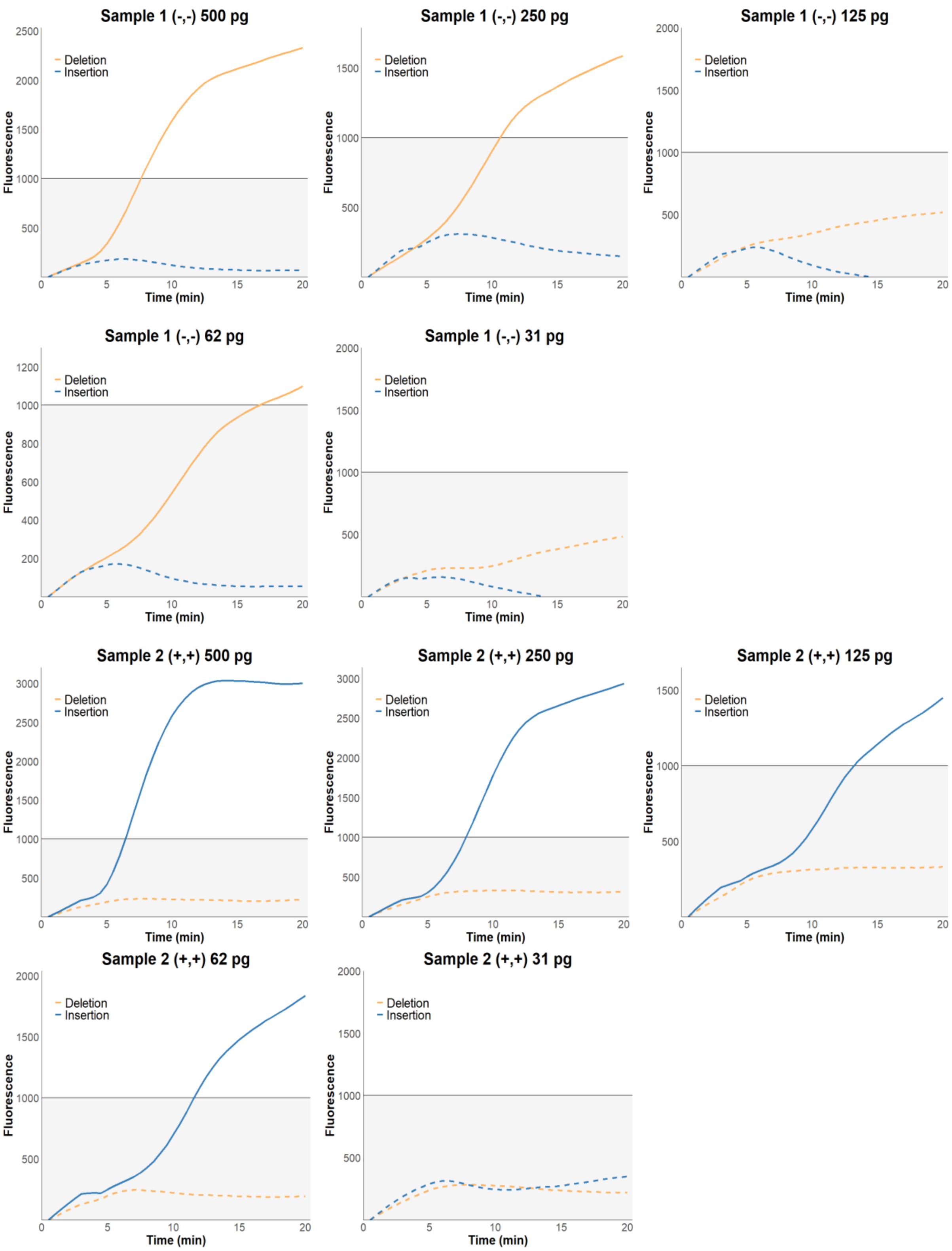

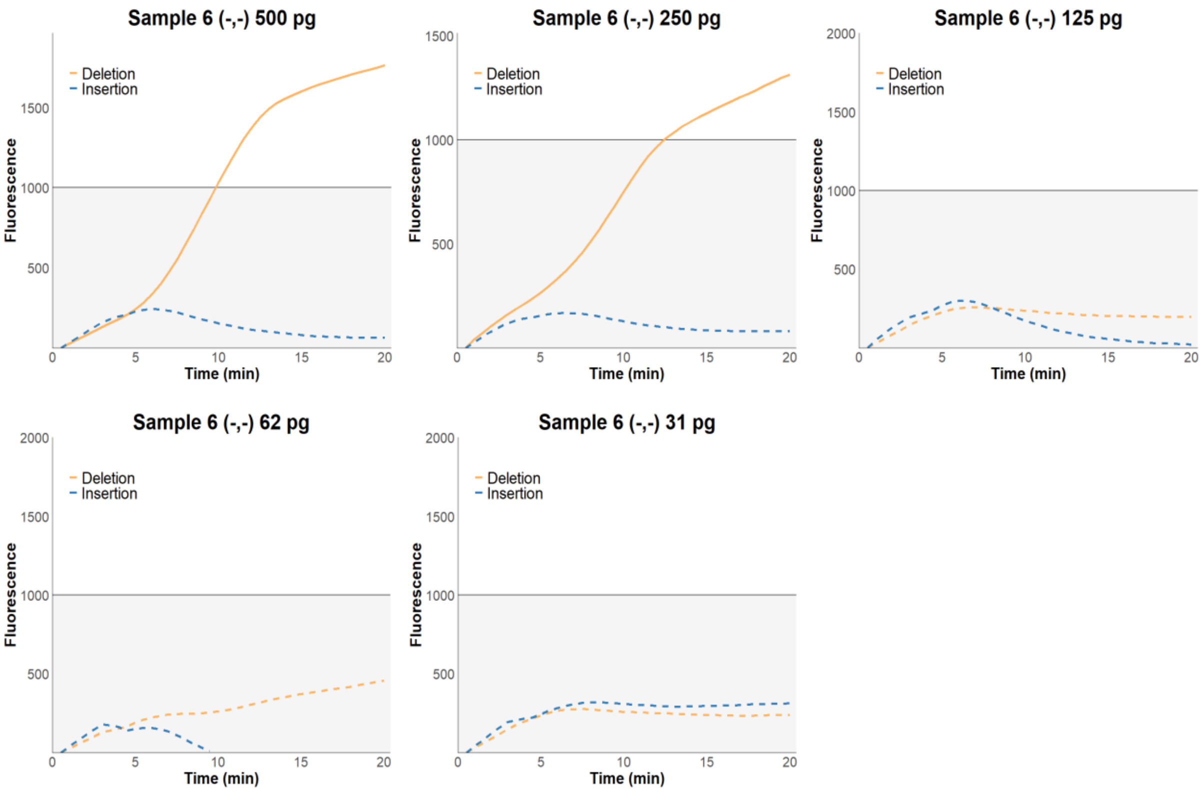
Sample 6 (-,-) 500 pg
Deletion
Insertion
Fluorescence
Time (min)
Sample 6 (-,-) 250 pg
Deletion
Insertion
Fluorescence
Time (min)
Sample 6 (-,-) 125 pg
Deletion
Insertion
Fluorescence
Time (min)
Sample 6 (-,-) 62 pg
Deletion
Insertion
Fluorescence
Time (min)
Sample 6 (-,-) 31 pg
Deletion
Insertion
Fluorescence
Time (min)

Figure S18: Sensitivity assessment for locus ID04. Real-time RPA graphs obtained for sample 1,2 and 6 are given for decreasing DNA inputs (500 – 31 pg). Sample name, true genotype and input levels are indicated above each graph. Curves of alleles that are called as present are represented as full lines, while curves of not-called alleles are represented as dashed lines. The horizontal grey line displays the 1000 unit threshold.

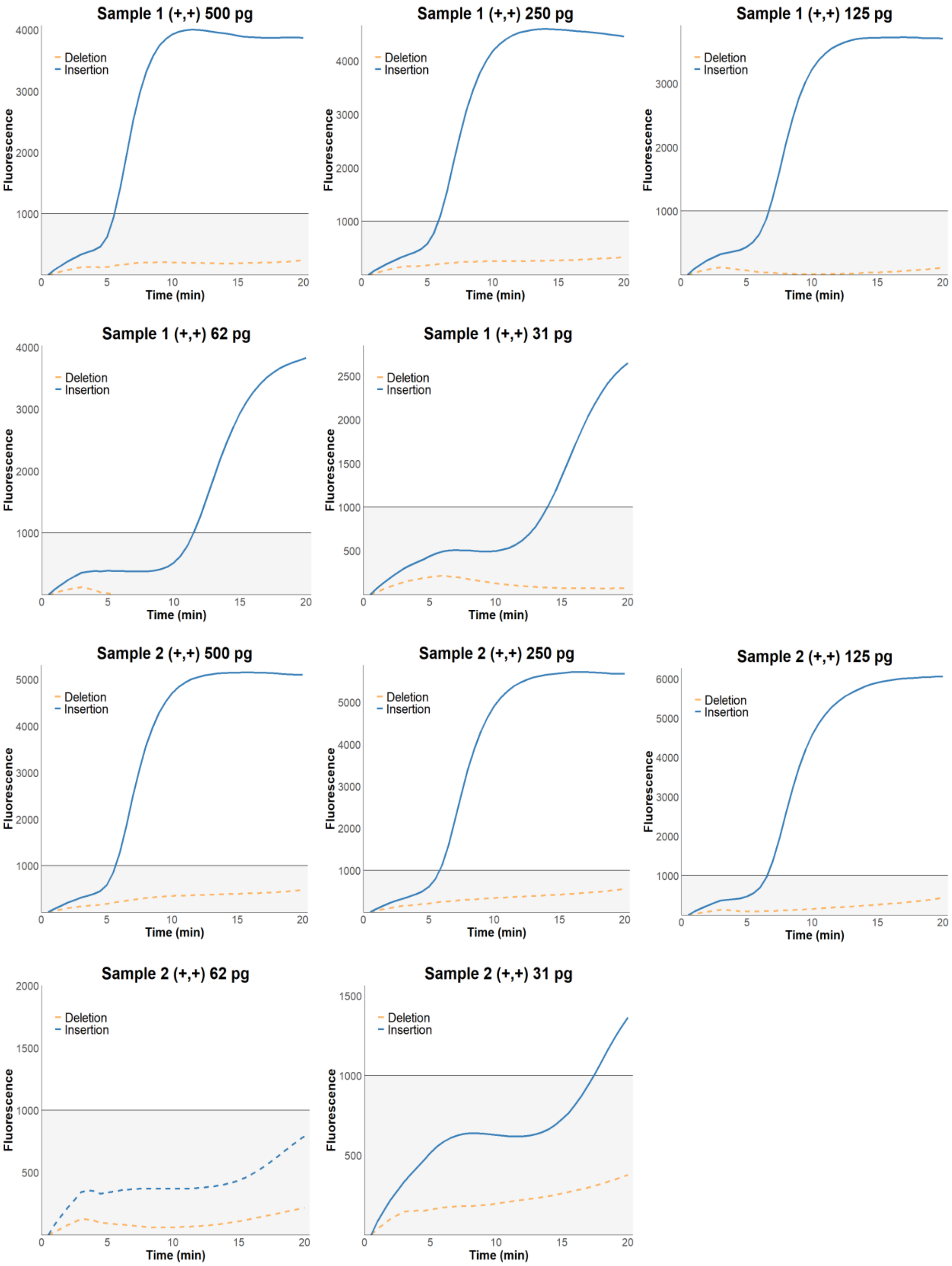

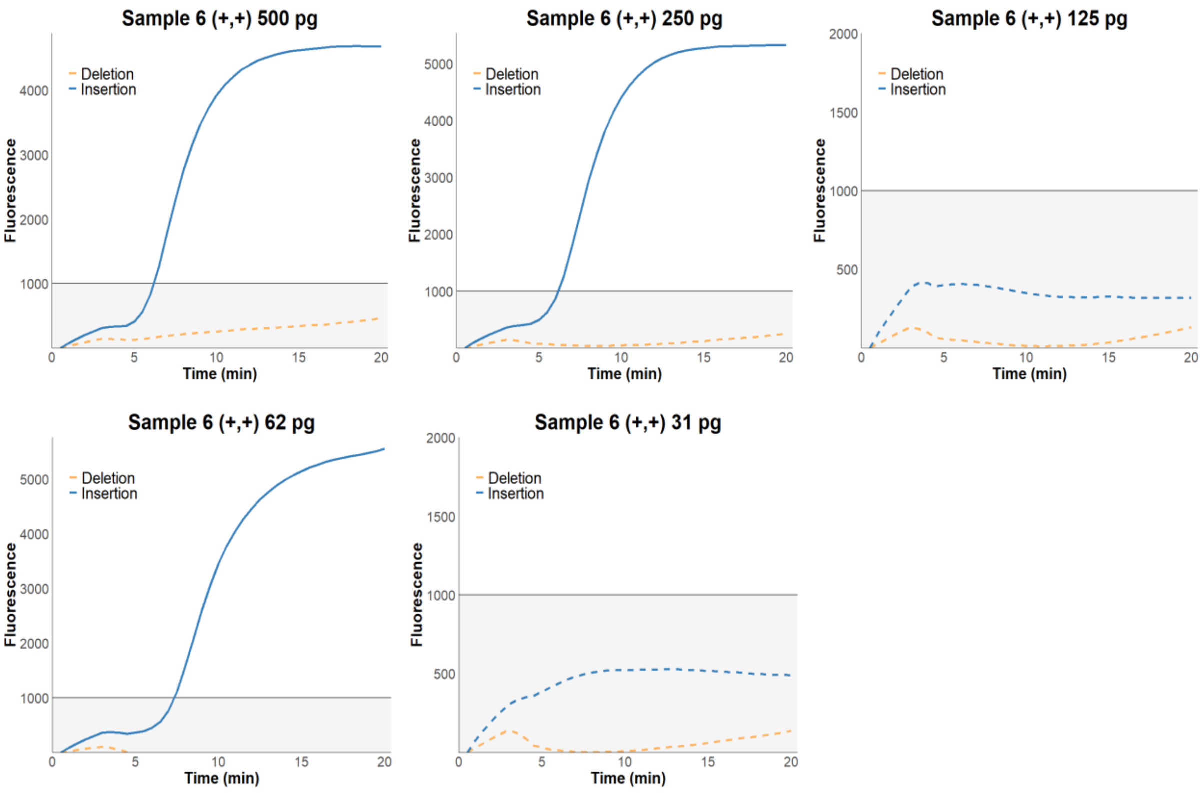
Sample 6 (+,+) 500 pg
Deletion
Insertion
Fluorescence
Time (min)
Sample 6 (+,+) 250 pg
Deletion
Insertion
Fluorescence
Time (min)
Sample 6 (+,+) 125 pg
Deletion
Insertion
Fluorescence
Time (min)
Sample 6 (+,+) 62 pg
Deletion
Insertion
Fluorescence
Time (min)
Sample 6 (+,+) 31 pg
Deletion
Insertion
Fluorescence
Time (min)

Figure S19: Sensitivity assessment for locus ID05. Real-time RPA graphs obtained for sample 1,2 and 6 are given for decreasing DNA inputs (500 – 31 pg). Sample name, true genotype and input levels are indicated above each graph. Curves of alleles that are called as present are represented as full lines, while curves of not-called alleles are represented as dashed lines. The horizontal grey line displays the 1000 unit threshold.

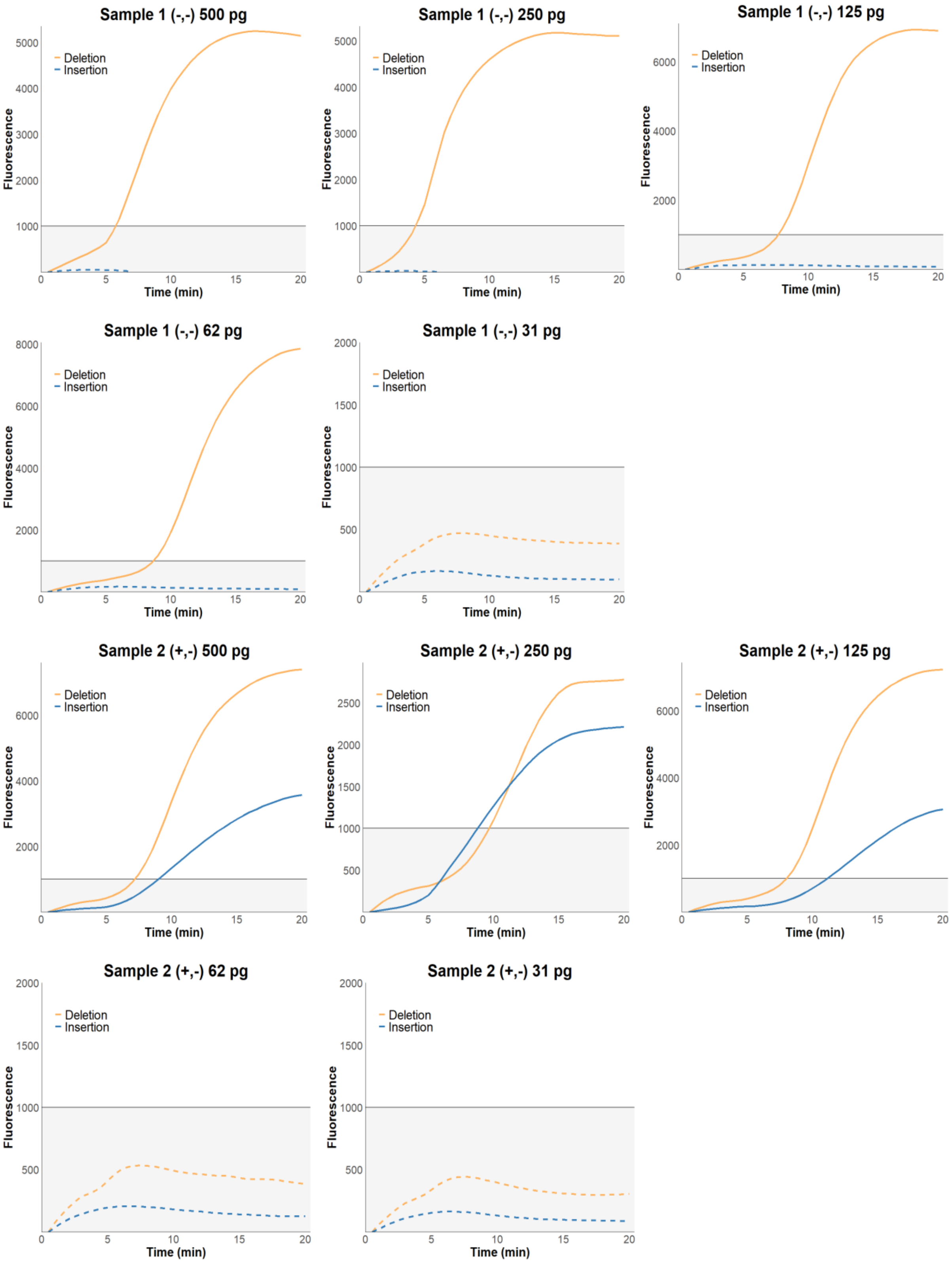

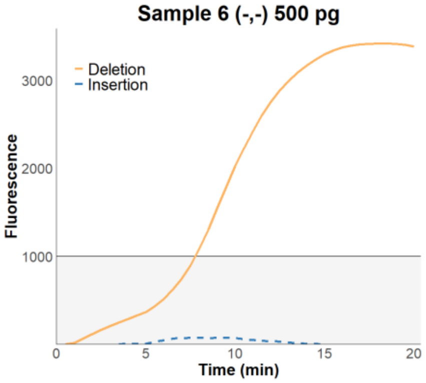
Sample 6 (-,-) 500 pg
Deletion
Insertion
Fluorescence
3000
2000
1000
0
5
10
15
20
Time (min)

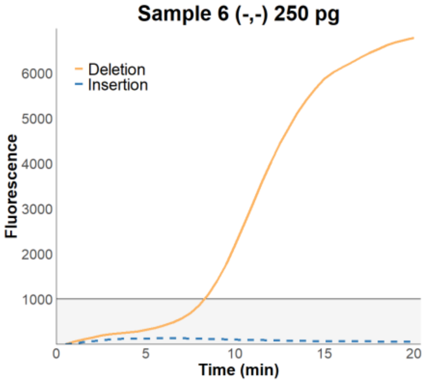
Sample 6 (-,-) 250 pg
Deletion
Insertion
Fluorescence
6000
5000
4000
3000
2000
1000
0
5
10
15
20
Time (min)

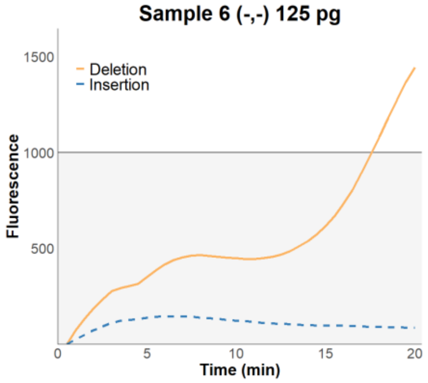
Sample 6 (-,-) 125 pg
Deletion
Insertion
Fluorescence
1500
1000
500
0
5
10
15
20
Time (min)

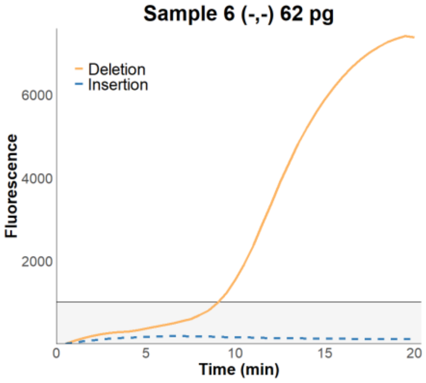
Sample 6 (-,-) 62 pg
Deletion
Insertion
Fluorescence
6000
4000
2000
0
5
10
15
20
Time (min)

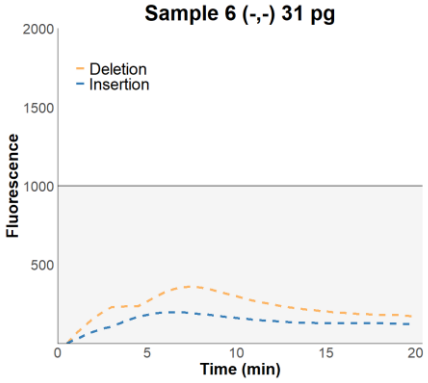
Sample 6 (-,-) 31 pg
Deletion
Insertion
Fluorescence
2000
1500
1000
500
0
5
10
15
20
Time (min)

<u>Figure S20:</u> Sensitivity assessment for locus ID06. Real-time RPA graphs obtained for sample 1,2 and 6 are given for decreasing DNA inputs (500 – 31 pg). Sample name, true genotype and input levels are indicated above each graph. Curves of alleles that are called as present are represented as full lines, while curves of not-called alleles are represented as dashed lines. The horizontal grey line displays the 1000 unit threshold.

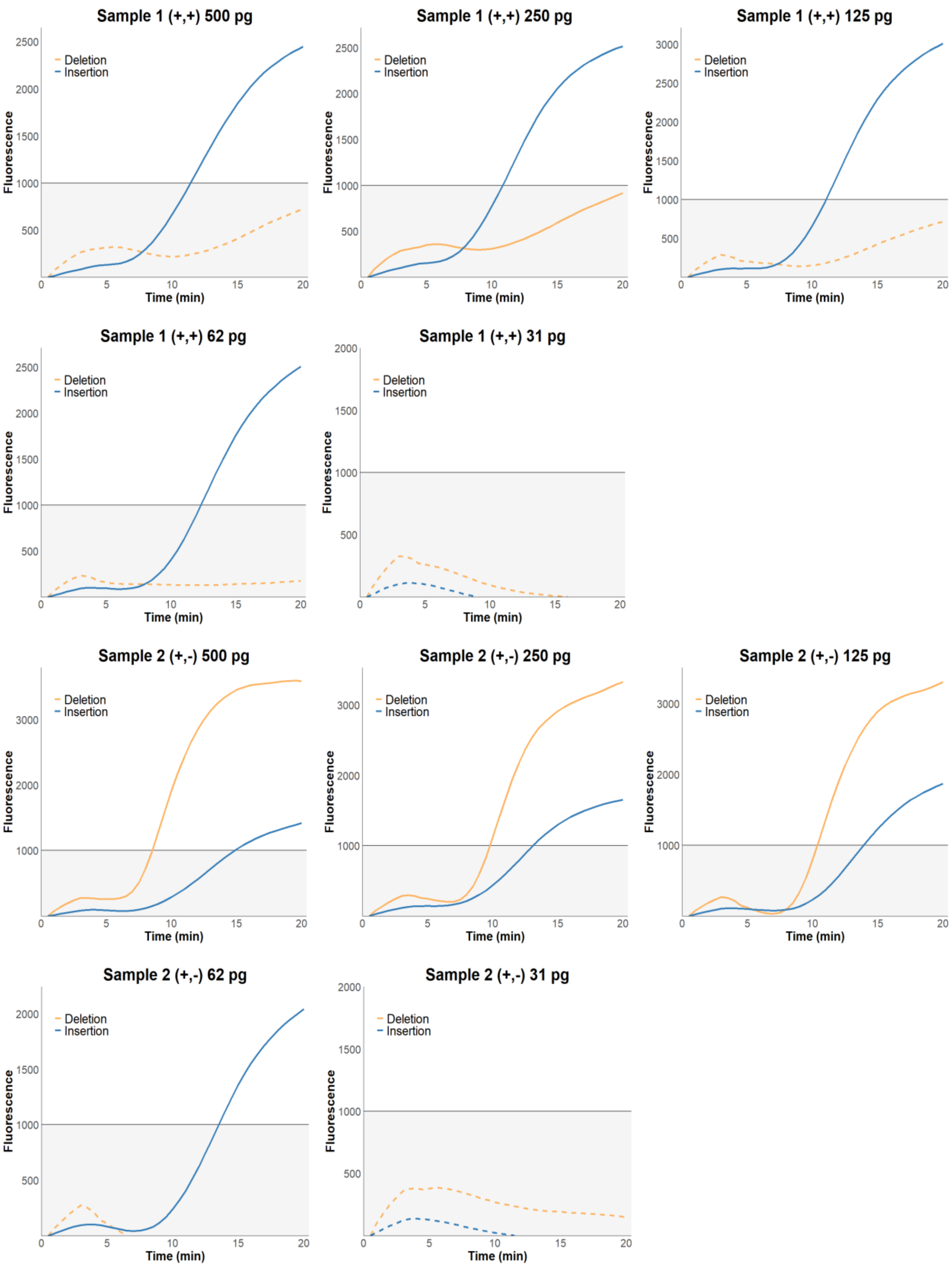

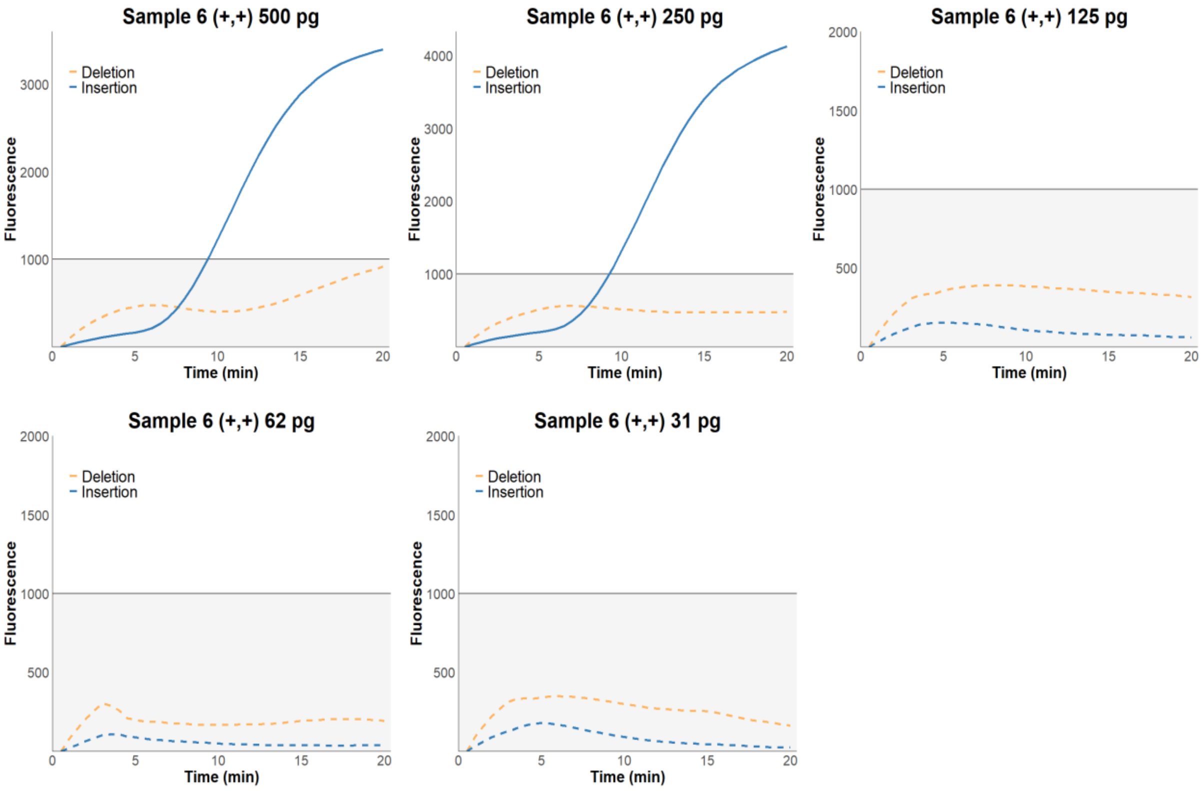
Sample 6 (+,+) 500 pg
Deletion
Insertion
Fluorescence
Time (min)
Sample 6 (+,+) 250 pg
Deletion
Insertion
Fluorescence
Time (min)
Sample 6 (+,+) 125 pg
Deletion
Insertion
Fluorescence
Time (min)
Sample 6 (+,+) 62 pg
Deletion
Insertion
Fluorescence
Time (min)
Sample 6 (+,+) 31 pg
Deletion
Insertion
Fluorescence
Time (min)

Figure S21: Sensitivity assessment for locus ID07. Real-time RPA graphs obtained for sample 1,2 and 6 are given for decreasing DNA inputs (500 – 31 pg). Sample name, true genotype and input levels are indicated above each graph. Curves of alleles that are called as present are represented as full lines, while curves of not-called alleles are represented as dashed lines. The horizontal grey line displays the 1000 unit threshold.

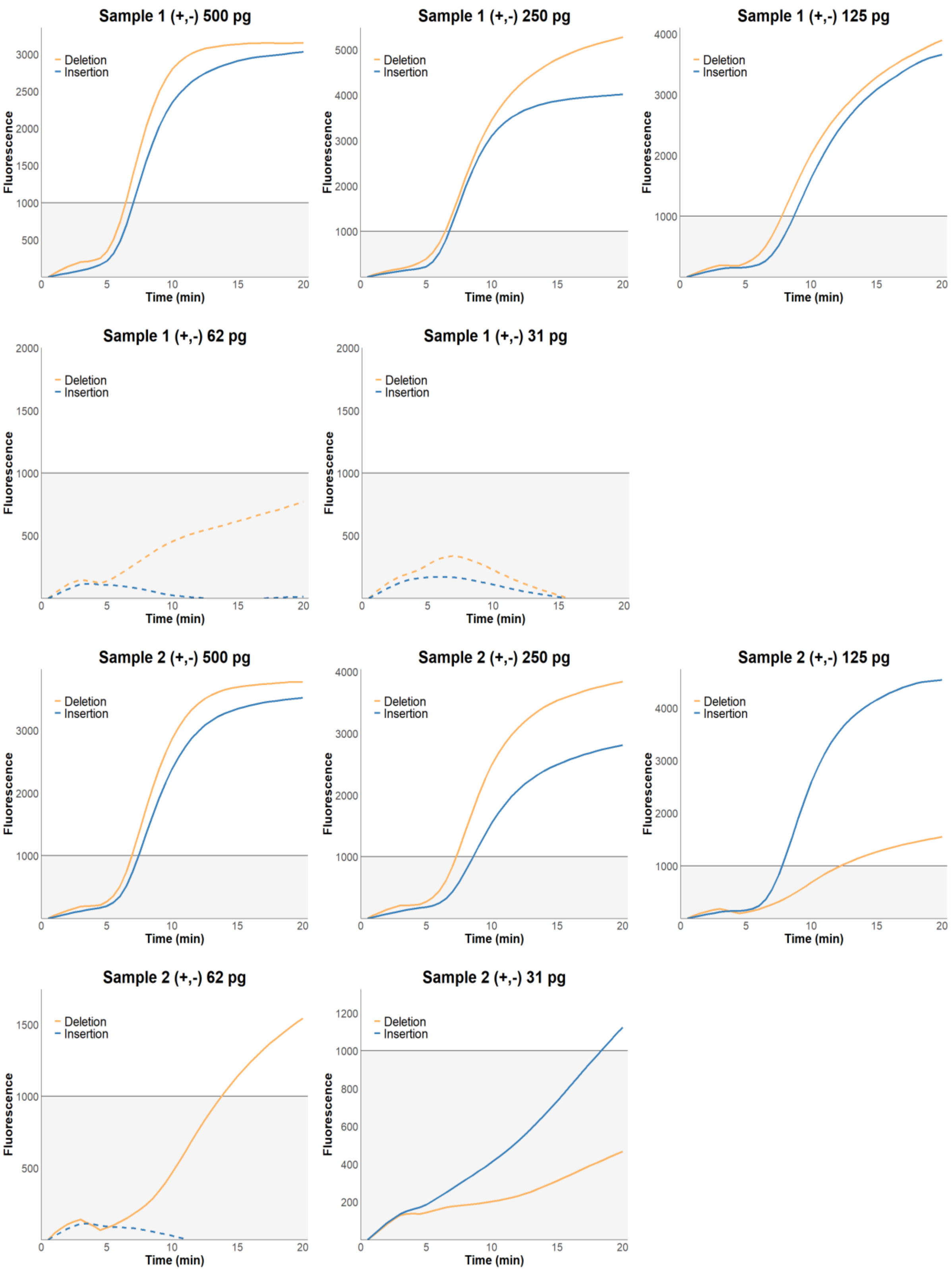

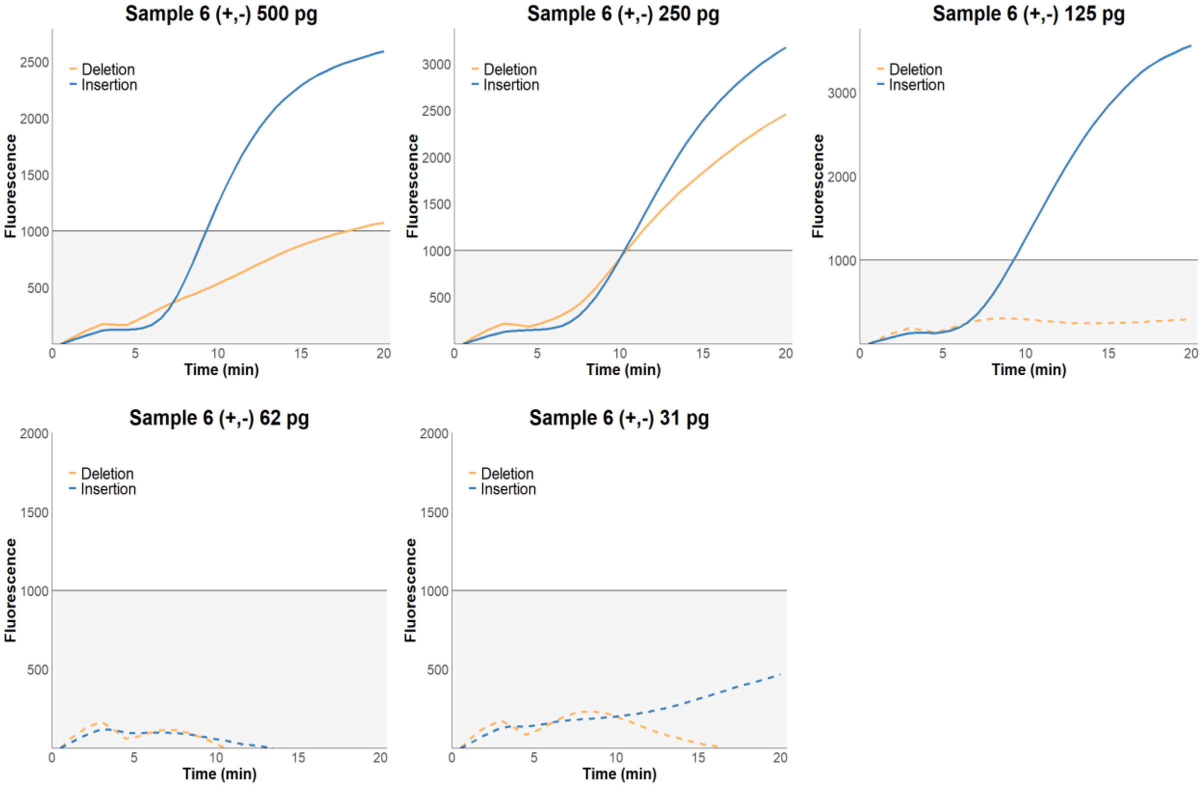
Sample 6 (+,-) 500 pg
Deletion
Insertion
Fluorescence
Time (min)
Sample 6 (+,-) 250 pg
Deletion
Insertion
Fluorescence
Time (min)
Sample 6 (+,-) 125 pg
Deletion
Insertion
Fluorescence
Time (min)
Sample 6 (+,-) 62 pg
Deletion
Insertion
Fluorescence
Time (min)
Sample 6 (+,-) 31 pg
Deletion
Insertion
Fluorescence
Time (min)
